# Comparative Proteomic Analysis in Scar-Free Skin Regeneration in *Acomys cahirinus* and Scarring *Mus musculus*

**DOI:** 10.1038/s41598-019-56823-y

**Published:** 2020-01-13

**Authors:** Jung Hae Yoon, Kun Cho, Timothy J. Garrett, Paul Finch, Malcolm Maden

**Affiliations:** 1 Department of Biology & UF Genetics Institute, 2033 Mowry Road, University of Florida, Gainesville, Florida 32610 USA; 2 Biomedical Omics Group, Korea Basic Science Institute, Ochang, 863-883 Republic of Korea; 3 Department of Pathology, Immunology, and Laboratory Medicine, University of Florida, Gainesville, Florida USA; 4 School of Biological Sciences, Royal Holloway, University of London, Egham, Surrey UK

**Keywords:** Protein-protein interaction networks, Non-model organisms

## Abstract

The spiny mouse, *Acomys cahirinus* displays a unique wound healing ability with regeneration of all skin components in a scar-free manner. To identify orchestrators of this regenerative response we have performed proteomic analyses of skin from *Acomys* and *Mus musculus* before and after wounding. Of the ~2000 proteins identified many are expressed at similar levels in *Acomys* and *Mus*, but there are significant differences. Following wounding in *Mus* the complement and coagulation cascades, PPAR signaling pathway and ECM-receptor interactions predominate. In *Acomys*, other pathways predominate including the Wnt, MAPK, the ribosome, proteasome, endocytosis and tight junction pathways. Notable among *Acomys* specific proteins are several ubiquitin-associated enzymes and kinases, whereas in *Mus* immuno-modulation proteins characteristic of inflammatory response are unique or more prominent. ECM proteins such as collagens are more highly expressed in *Mus*, but likely more important is the higher expression of matrix remodeling proteases in *Acomys*. Another distinctive difference between *Acomys* and *Mus* lies in the macrophage-produced arginase 1 is found in *Mus* whereas arginase 2 is found in *Acomys*. Thus, we have identified several avenues for experimental approaches whose aim is to reduce the fibrotic response that the typical mammal displays in response to wounding.

## Introduction

The cellular and molecular events of full thickness wound repair in mammalian skin occur in three overlapping phases namely inflammation, tissue formation and tissue remodeling, the typical outcome of which is scar tissue composed of non-physiologic dermal tissue masked by smooth, hairless epidermis^1^. However, there are several examples where wound repair involves complete regeneration rather than scarring, including adult fish^2^, Urodele skin^3,4^ and fetal mammalian skin up to the end of the second trimester^5–7^. Comparisons between fetal and adult mammalian wounds^5–7^ have led to the identification of distinct differences in fetal skin wounding including less robust immune responses, lower levels of inflammatory cytokines and growth factors such as Pdgfa and Tgfβ1^8,9^ and differences in matrix composition which may also be relevant to the successful outcomes of fish and Urodele skin regeneration.

Surprisingly, there are some adult mammals in which the skin can regenerate after injury such as punches through the ears of rabbits^10^, spiny mice, *Acomys*^11^, small ear wounds in MRL mice^12^ and large skin wound in young C57B/L mice^13^, suggesting that this may not be a property solely of lower vertebrates and fetuses. Several species of *Acomys* can not only regenerate all the components of the ear viz. cartilage, adipose tissue and hair in a scar-free manner^11,14,15^ but also all the components of skin after full thickness wounding or burn injury^11,16^. Comparisons between skin regeneration in *Acomys* and skin scarring after the same injury in *Mus* has revealed striking similarities between *Acomys* and fetal wound healing including absent or low levels of pro-inflammatory cytokines in *Acomys*, reduced levels of F4/80 macrophages and very different ECM components especially, excessive collagens in *Mus* but not in *Acomys*^17,18^. The intervention of macrophages is, however, necessary for regeneration even though they may not be present at the wound site^19^ and an acute inflammatory response with strong myeloperoxidase activity was exhibited in both *Acomys* and *Mus*, but with stronger ROS production in *Acomys*.

At present much of the information about differences between *Acomys* and *Mus* is derived from cellular and genetic analyses^11,14–19^ and the involvement of proteins is more by implication than by direct observation, so a more comprehensive proteomic study would be desirable. In the study presented here, we have qualitatively and quantitatively compared the proteomic profiles of untreated and wounded skin of *Mus* and *Acomys* to identify proteins that potentially favor scar-free healing. Among the ca. 2000 proteins we identified the majority were expressed at similar levels by *Acomys* and *Mus*. However distinct differences were found in the levels of ubiquitin-related enzymes, phosphorylation-associated proteins, proteases, immunomodulators and macrophage markers. We find that the enhanced degradation and synthesis of proteins is a major mechanism in *Acomys*, especially ubiquitination and phosphorylation which may play a critical role in regulating the signaling pathways employed in tissue repair. In addition, the disparate response in terms of macrophage profiles may generate different ECM microenvironments which are critical to the outcome of injury, namely fibrosis in *Mus* vs a regenerative response in *Acomys*.

## Results

### Comprehensive global proteome profiling of *Acomys* and *Mus* skin

To gain insight into the potential underlying molecular mechanisms, we performed shotgun proteomics by 1D gel separation / nano-LC-MS/MS on protein extracts from *Acomys* and *Mus* skin at days 0 (unwounded), 3, 5, 7 and 14 post-wounding. To acquire comprehensive proteomic profiles of the skin, a workflow was developed and the general scheme for sample preparation and analysis is given in Supplementary Fig. S1.

**Figure 1:**
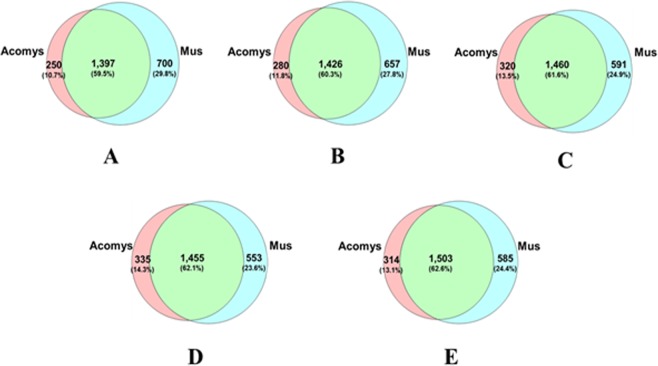
Venn diagrams of common and unique proteins identified between *Acomys* and *Mus* at day 0 (**A**), 3 (**B**), 5 (**C**), 7 (**D**) and 14 (**E**).

Protein identification was carried out by searching against the mouse database (UniprotKBMusmusculus) since our previous data showed that several protein sequences in *Acomys* were 96% homologous to those of *Mus*^18^ and an alignment of several proteins used for identification of macrophage subsets between *Acomys* and *Mus* revealed 80% to 100% nucleotide identity^19^. Here we have also compared the known *Acomys cahirinus* protein sequences with proteins from *Mus musculus* and shown they all have 85% +/− 2% sequence homology (Supplementary Table S1). Our recent comparative transcriptomic analysis of skin wound healing has demonstrated that the identification of 21663 orthologs between two species, confirming the close similarity of transcript levels^20^. As a result, we identified totals of 1647, 1706, 1780, 1790 and 1817 non-redundant proteins in *Acomys* at days 0, 3, 5, 7, 14, respectively. The corresponding numbers of proteins identified in *Mus* were 2097, 2083, 2051, 2008 and 2088. The total numbers of unique and common proteins at the different time points from both species is shown in Fig. 1. On average over the sample times the number of proteins identified that were unique to *Mus was* 26.1 ± 2.6%, unique to *Acomys* 12.7 ± 1.4% and common to both 61.2 ± 1.3%. Over all time points, 494 and 473 proteins were differentially present in *Acomys* or in *Mus*, respectively.

### Proteomic analysis of normal *Acomys* and *Mus* skin

**Figure 2:**
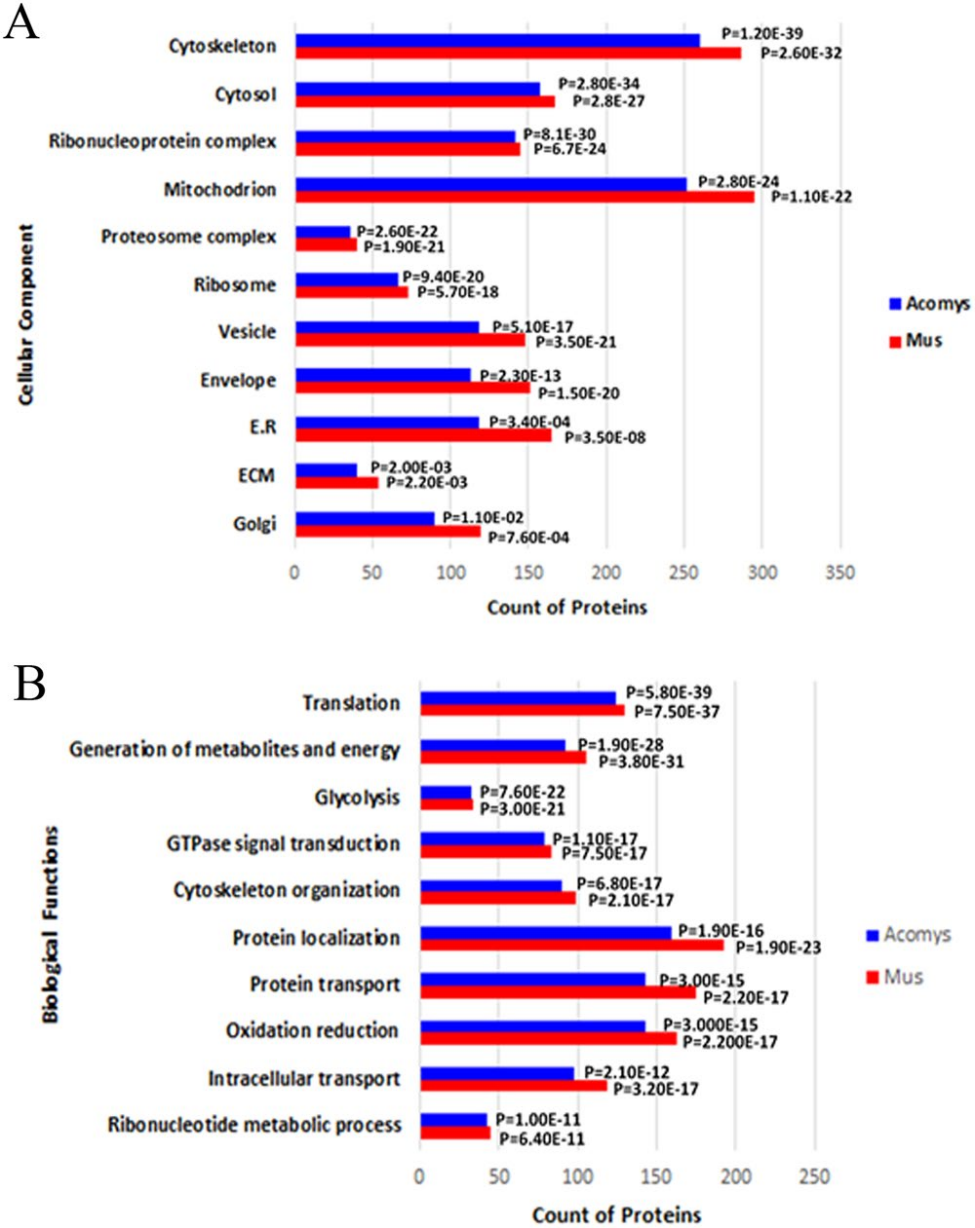
Gene ontology analyses of protein counts versus (**A**) cellular components (**B**) biological functions of identified proteins in *Acomys* and *Mus* at day 0.

To elucidate whether or not the protein profiles would reveal intrinsic biological differences between *Acomys* and *Mus* before wounding we performed Gene ontology (GO) enrichment analyses with total proteins detected from both species, according to their location in the cell components (Fig. 2A) and related biological functions (Fig. 2B) at day 0. The cellular locations of the identified proteins were highest for the cytoskeleton and mitochondrion but showed a similar distribution between the two species. Likewise, the biological functions of the identified proteins were highest for protein localization, protein transport and oxidation reduction, but showed a similar distribution between species.

A list of common and unique proteins is shown in Table S2 revealing that there were very similar protein profiles in *Acomys* and *Mus* with regard to the presence of the most abundantly reported mouse skin proteins such as keratins (see also Table 1 and Table S4), myosins, actins and heat-shock proteins. The collagens were generally present at higher levels in *Mus* skin (see also Table 1) as well as tenascin. However, the unique proteins identified in skin samples from each species at day 0 (see Table 1) showed distinct biological characteristics. *Acomys* specific proteins were involved in protein amino acid phosphorylation such as tyrosine protein kinases (BLK, CSK, FGR, FGFR1, FRK, MAP2K1) and serine/threonine protein kinases (CDKs, STK10, RPS6KA1) and cell division, whereas *Mus* specific proteins belong to immune defense and wound response processes including several complement components, proteases (kallikreins B, cathepsin H and L1) and protease inhibitors (Serpina1 and Serpina3 isomers, (see also Table S5). These unique proteins in each species might allow dramatically different functions that result in intrinsic biological differences after wounding.

**Table 1:** Proteins identified from *Acomys* and *Mus* associated with wound healing over 14 days.

Accession	Protein Description	Gene	Acomys					Mus				
			0 day	3 days	5 days	7 days	14days	0 day	3 days	5 days	7 days	14 days
			Quantitative Value (CV)									
**Ubiquitin/Proteasome**												
O88685	26S protease regulatory subunit 6A	Psmc3	1.55(20.4)	4.43(9.6)	4.19(5.9)	3.39(1.3)	4.32(6.1)	3.35(15.7)	3.04(4.2)	3.62(6.8)	3.05(5.5)	2.73(15.9)
Q6ZPJ3	E2/E3 hybrid ubiquitin-protein ligase UBE2O	Ube2o	1.04(19.2)	4.45(5.5)	4.02(7.8)	4.00(10.3)	3.14(13.6)	3.32(15.9)	2.04(8.9)	1.61(18.6)	1.35(14.8)	1.73(13.5)
Q3U319	E3 ubiquitin-protein ligase BRE1B	Rnf40	1.55(17.9)	2.23(22.4)	2.04(19.6)	3.02(13.9)	2.51(19.6)	3.77(18.8)	2.63(4.9)	3.13(9.1)	2.76(8.9)	3.17(13.1)
P46935	E3 ubiquitin-protein ligase NEDD4	Nedd4	3.34(21.8)	2.84(21.3)	4.82(97.8)	6.05(11.1)	6.51(14.5)	3.23(13.3)	3.08(4.2)	2.59(4.5)	2.93(17.1)	3.86(11.0)
P0CG49	Polyubiquitin-B	Ubb	3.16(5.1)	4.66(17.7)	5.28(5.0)	5.92(1.6)	5.14(19.4)	4.07(21.1)	3.61(4.5)	3.30(7.5)	2.33(13.5)	5.50(19.2)
P0CG50	Polyubiquitin-C	Ubc		5.26(3.7)	5.32(8.2)	5.93(1.7)	4.95(21.3)	5.47(21.3)	4.89(12.2)	4.67(6.7)	4.53(19.5)	6.66(9.5)
Q9Z2U1	Proteasome subunit alpha type-5	Psma5	2.05(19.1)	3.93(16.3)	4.47(3.4)	3.75(10.7)	4.10(19.0)	4.70(12.8)	5.42(7.3)	4.97(14.3)	4.98(5.0)	5.58(6.3)
Q6ZQ93	Ubiquitin carboxyl-terminal hydrolase 34	Usp34	0.94(16.4)	1.86(16.1)	1.69(17.7)	1.64(18.3)	1.62(12.3)					
Q6A4J8	Ubiquitin carboxyl-terminal hydrolase 7	Usp7	1.04(19.2)	1.42(21.1)	1.74(17.3)	1.83(16.4)	2.00(16.1)					
P62984	Ubiquitin-60S ribosomal protein L40	Uba52	2.23(8.3)	5.26(3.7)	5.32(5.8)	5.93(1.7)	5.28(18.9)	5.02(2.4)	5.38(10.3)	5.34(2.5)	4.84(1.9)	5.42(5.5)
P52482	Ubiquitin-conjugating enzyme E2 E1	Ube2e1	1.84(14.6)	3.34(15.6)	1.87(13.3)	1.79(2.5)	3.82(13.1)					
Q8K2Z8	Ubiquitin-conjugating enzyme E2 Q2	Ube2q2	2.45(19.5)	2.50(16.0)	2.51(17.7)	1.44(14.5)	3.51(4.3)					
Q02053	Ubiquitin-like modifier-activating enzyme 1	Uba1	4.53(14.8)	4.99(21.4)	5.41(2.4)	5.45(7.2)	5.34(18.0)	5.93(6.4)	5.56(13.1)	5.37(19.6)	5.57(15.4)	7.28(2.3)
**Ribosome**												
P62301	40S ribosomal protein S13	Rps13	1.55(17.9)	3.05(6.1)	4.13(13.6)	4.49(8.0)	3.92(8.0)	3.35(15.7)	3.04(4.2)	3.62(6.8)	3.05(5.5)	2.73(15.9)
P63276	40S ribosomal protein S17	Rps17	1.55(17.9)	1.86(16.1)	3.01(10.5)	4.20(10.4)	2.89(3.4)	3.32(15.9)	2.04(8.9)	1.61(18.6)	1.35(14.8)	1.73(13.5)
P62855	40S ribosomal protein S26	Rps26	0.94(16.4)	2.05(9.1)	3.82(20.1)	3.47(20.6)	3.89(2.6)	3.77(18.8)	2.63(4.9)	3.13(9.1)	2.76(8.9)	3.17(13.1)
P19253	60S ribosomal protein L13a	Rpl13a	1.05(21.3)	2.37(16.5)	4.90(17.2)	3.94(15.0)	3.47(13.0)	3.23(13.3)	3.08(4.2)	2.59(4.5)	2.93(17.1)	3.86(11.0)
P14115	60S ribosomal protein L27a	Rpl27a	1.55(17.9)	4.58(9.0)	4.65(18.4)	3.81(13.1)	4.21(13.0)	4.07(21.1)	3.61(4.5)	3.30(7.5)	2.33(13.5)	5.50(19.2)
Q9D8E6	60S ribosomal protein L4	Rpl4	3.00(19.7)	5.12(5.9)	5.01(19.6)	5.48(5.7)	7.04(1.1)	5.47(21.3)	4.89(12.2)	4.67(6.7)	4.53(19.5)	6.66(9.5)
P62983	Ubiquitin-40S ribosomal protein S27a	Rps27a	2.23(11.7)	4.66(17.7)	5.39(6.2)	5.93(1.7)	5.02(15.0)	4.70(12.8)	5.42(7.3)	4.97(14.3)	4.98(5.0)	5.58(6.3)
**Spliceosome**												
O08810	116 kDa U5 small nuclear ribonucleoprotein component	Eftud2	1.73(17.4)	2.05(9.1)	4.39(7.9)	4.36(12.3)	5.21(17.9)	3.78(13.0)	4.17(17.7)	3.80(2.4)	3.79(16.2)	5.66(8.6)
P17879	Heat shock 70 kDa protein 1B	Hspa1b	5.50(3.5)	4.83(20.6)	5.74(8.7)	5.88(2.0)	2.10(13.2)	4.77(16.5)	5.78(3.3)	5.40(3.1)	5.82(5.2)	7.16(0.2)
P49312	Heterogeneous nuclear ribonucleoprotein A1	Hnrnpa1	0.94(16.4)	2.43(15.4)	4.53(3.6)	5.03(11.9)	5.44(8.4)	4.22(13.9)	3.69(16.9)	3.22(15.5)	2.74(8.2)	5.24(17.2)
Q62093	Serine/arginine-rich splicing factor 2	Srsf2	1.66(17.2)	1.64(13.4)	2.82(6.8)	3.39(1.3)	4.00(9.5)	2.41(17.9)	1.79(21.5)	2.59(6.4)	2.87(14.1)	3.32(13.1)
P62317	Small nuclear ribonucleoprotein Sm D2	Snrpd2	1.27(15.7)	1.86(16.1)	3.66(14.8)	4.42(12.9)	2.89(3.4)	2.30(14.1)	2.54(20.6)	1.12(17.8)	1.62(18.5)	2.23(4.2)
Q9Z1N5	Spliceosome RNA helicase Ddx39b	Ddx39b	2.55(20.5)	4.36(16.9)	5.23(11.8)	5.54(6.6)	5.58(10.9)	4.40(5.0)	4.48(2.2)	4.36(7.6)	4.11(19.9)	5.67(1.8)
Q921M3	Splicing factor 3B subunit 3	Sf3b3	1.94(7.9)	2.05(9.1)	5.43(9.6)	5.39(2.8)	5.33(16.6)	4.01(19.9)	4.42(11.3)	4.03(15.4)	3.86(18.5)	5.33(3.7)
**Protein Processing in Endoplasmic Reticulum**												
P14211	Calreticulin	Calr	3.56(11.5)	2.55(15.2)	4.99(12.2)	5.29(18.8)	5.48(10.5)	4.57(11.8)	4.83(3.1)	5.21(12.4)	5.34(12.3)	5.81(9.7)
P35564	Calnexin	Canx	3.13(13.5)	4.73(18.0)	4.39(21.0)	4.79(11.6)	5.54(1.4)	5.16(8.8)	5.14(17.9)	5.14(6.2)	4.77(12.1)	6.06(9.0)
O54734	Dolichyl-diphosphooligosaccharide–protein glycosyltransferase 48 kDa subunit	Ddost	1.05(11.7)	2.05(12.9)	4.32(1.0)	3.84(14.5)	4.63(11.6)	3.95(12.4)	3.92(14.6)	4.08(13.1)	3.21(12.2)	5.27(4.7)
Q922R8	Protein disulfide-isomerase A6	Pdia6	2.55(15.4)	4.72(14.3)	5.36(6.5)	5.89(2.0)	5.85(3.4)	5.05(8.5)	5.29(9.3)	5.45(1.4)	4.96(15.4)	6.32(9.9)
Q91W90	Thioredoxin domain-containing protein 5	Txndc5	1.94(7.9)	3.74(13.4)	3.18(14.0)	3.10(10.9)	4.14(10.3)	2.76(21.7)	1.04(17.5)	3.41(15.3)	3.12(16.2)	5.45(4.1)
**Keratin**												
Q61765	Keratin, type I cuticular Ha1	Krt31	8.16(14.5)	7.11(15.9)	6.55(15.6)	7.48(14.4)	7.74(11.6)	6.16(12.8)	6.48(17.0)	6.23(9.5)	6.32(3.0)	7.77(8.5)
Q62168	Keratin, type I cuticular Ha2	Krt32	7.74(19.6)	7.04(16.0)	6.09(21.1)	7.13(9.1)	7.75(11.8)	5.84(12.2)	5.96(10.6)	6.08(6.3)	5.99(11.2)	6.57(2.0)
Q61897	Keratin, type I cuticular Ha3-II	Krt33b	7.93(13.7)	7.01(14.5)	6.55(15.6)	7.41(14.7)	7.73(11.5)	6.12(12.3)	6.46(16.4)	6.25(9.9)	6.30(4.9)	7.68(7.3)
Q497I4	Keratin, type I cuticular Ha5	Krt35	7.89(15.4)	7.00(14.2)	6.17(18.5)	7.31(12.2)	7.76(11.8)	6.00(11.0)	6.28(13.0)	6.25(8.0)	6.26(6.3)	7.15(4.8)
B1AQ75	Keratin, type I cuticular Ha6	Krt36	7.78(17.6)	6.86(14.4)	5.84(21.9)	7.13(9.6)	7.61(10.5)	5.80(12.9)	5.96(10.6)	6.07(6.1)	5.99(11.2)	6.64(1.3)
Q9QWL7	Keratin, type I cytoskeletal 17	Krt17	9.12(5.0)	9.20(12.2)	8.78(7.5)	9.50(1.9)	9.36(4.7)	8.93(3.7)	8.16(6.1)	8.57(1.8)	8.46(2.8)	9.04(1.4)
**Protein Phosphorylation**												
P11440	Cyclin-dependent kinase 1	Cdk1	1.27(15.7)	1.42(14.1)	2.37(11.6)	3.42(0.5)	1.94(20.2)					
P31938	Dual specificity mitogen-activated protein kinase kinase 1	Map2k1	1.95(14.2)	3.74(13.4)	4.23(4.5)	3.34(16.1)	3.26(19.0)					
Q01279	Epidermal growth factor receptor	Egfr		1.42(14.1)	1.89(11.5)	2.44(12.3)	1.60(19.6)	3.10(10.1)	5.82(4.3)	4.11(22.0)	4.57(19.5)	4.67(5.4)
P42567	Epidermal growth factor receptor substrate 15	Eps15		1.86(16.1)	1.74(11.5)	2.44(12.3)	1.50(11.5)	2.07(18.7)	1.40(14.3)			2.79(10.3)
P16092	Fibroblast growth factor receptor 1	Fgfr1	1.27(15.7)	1.42(14.1)	1.87(13.3)	2.92(4.7)	1.72(8.2)					
P18653	Ribosomal protein S6 kinase alpha-1	Rps6ka1	1.05(21.3)	1.42(14.1)	1.74(11.5)	1.83(10.9)	1.38(14.5)					
O55098	Serine/threonine-protein kinase 10	Stk10	1.16(14.0)	3.01(16.6)	1.74(11.5)	1.76(11.4)	1.62(12.3)					
P83741	Serine/threonine-protein kinase WNK1	Wnk1	1.04(9.6)	1.42(14.1)	1.89(11.5)	1.44(13.9)	1.60(19.6)		1.17(8.5)		2.35(12.8)	3.60(5.2)
P16277	Tyrosine-protein kinase Blk	Blk	1.27(15.7)	1.42(14.1)	1.87(13.3)	2.63(10.5)	1.72(8.2)					
P41241	Tyrosine-protein kinase CSK	Csk	2.63(15.2)	3.70(12.5)	3.02(17.5)	3.10(15.4)	3.14(13.6)					
P14234	Tyrosine-protein kinase Fgr	Fgr	1.27(15.7)	2.42(16.5)	1.87(13.3)	2.63(10.5)	1.72(8.2)					
Q922K9	Tyrosine-protein kinase FRK	Frk	2.63(15.2)	3.63(7.3)	3.18(19.8)	3.10(15.4)	2.80(14.8)					
**Protease**												
P10605	Cathepsin B	Ctsb	1.05(21.3)	2.83(21.0)	3.60(22.8)	2.79(12.7)	3.00(20.7)	5.16(16.1)	4.24(17.5)	5.24(14.7)	4.86(2.9)	5.15(15.0)
P18242	Cathepsin D	Ctsd	1.05(21.3)	2.14(13.1)	2.37(19.4)	3.59(4.8)	2.94(21.4)	4.01(17.3)	4.24(16.3)	3.63(18.1)	4.62(8.7)	4.79(1.1)
P28293	Cathepsin G	Ctsg		4.44(15.9)	3.02(17.5)	1.60(14.4)	2.10(18.7)					
P49935	Pro-cathepsin H	Ctsh						2.90(22.1)	2.04(8.9)	1.68(17.7)	1.35(14.8)	2.78(21.9)
P06797	Cathepsin L1	Ctsl						1.53(16.7)	1.17(8.5)	1.18(10.8)	1.46(11.4)	1.17(8.5)
Q9WUU7	Cathepsin Z	Ctsz	1.04(19.2)	2.37(16.5)	3.55(6.8)	3.64(5.5)	3.45(10.1)	3.15(12.1)	3.20(1.4)	3.80(12.1)	3.79(8.3)	3.15(12.3)
P26262	Plasma kallikrein	Klkb1						2.56(16.3)	6.19(3.3)	6.10(1.6)	6.96(2.4)	3.86(17.4)
P21812	Mast cell protease 4	Mcpt4	3.04(16.4)	2.42(12.4)	3.32(15.1)	3.34(15.0)	4.43(11.3)					
P41245	Matrix metalloproteinase-9	Mmp9							6.81(5.2)	6.26(12.2)	5.96(12.8)	2.97(16.8)
P21845	Tryptase beta-2	Tpsb2	1.04(9.6)	1.42(14.1)	1.42(14.1)	1.74(17.3)	1.62(18.5)					
**Protease Inhibitors**												
Q61247	Alpha-2-antiplasmin	Serpinf2		3.70(21.3)	3.66(1.2)	4.17(18.8)	2.27(9.7)	2.17(13.8)	6.12(1.4)	5.58(11.6)	6.70(2.5)	1.78(22.0)
Q61838	Alpha-2-macroglobulin	Pzp	2.63(19.0)	7.36(9.8)	6.91(3.3)	7.59(9.7)	6.27(8.2)	6.93(3.8)	10.39(3.6)	10.41(0.5)	10.75(2.5)	7.81(2.9)
Q6GQT1	Alpha-2-macroglobulin-P	A2mp	1.04(19.2)	7.32(12.8)	6.50(2.7)	7.79(12.6)	5.95(8.5)	2.71(12.4)	5.87(6.6)	5.99(1.6)	6.32(2.9)	3.83(15.3)
P32261	Antithrombin-III	Serpinc1	1.04(19.2)	6.00(12.1)	5.18(15.9)	5.69(14.1)	3.91(13.2)	2.38(19.6)	7.03(4.6)	6.21(6.2)	7.35(3.6)	4.17(16.3)
Q62426	Cystatin-B	Cstb						1.10(19.9)	0.91(10.9)	1.47(13.6)	1.58(12.7)	1.73(19.3)
P49182	Heparin cofactor 2	Serpind1	1.04(19.2)	2.05(12.9)	3.05(14.6)	3.29(22.8)	2.96(16.9)	2.27(13.1)	5.83(8.0)	5.42(8.2)	6.15(4.1)	3.44(2.1)
O08677	Kininogen-1	Kng1						2.65(18.7)	7.63(0.6)	6.83(6.3)	7.90(6.2)	3.70(14.3)
P12032	Metalloproteinase inhibitor 1	Timp1						0.58(8.6)	1.17(17.1)	2.71(14.8)	2.12(19.2)	
P97290	Plasma protease C1 inhibitor	Serping1	1.66(9.8)	6.04(9.8)	4.79(7.4)	5.51(4.7)	5.56(10.3)	3.27(20.7)	6.95(4.7)	5.96(5.6)	6.89(4.2)	4.34(20.7)
**ECM(Extra Cellular Matrix)**												
P11087	Collagen alpha-1(I) chain	Col1a1	2.71(22.8)	2.05(12.9)	5.08(20.0)	4.40(13.6)	5.02(17.5)	3.56(15.6)	4.11(11.1)	3.86(3.8)	3.90(6.6)	7.05(4.9)
P08121	Collagen alpha-1(III) chain	Col3a1	1.16(9.9)	1.42(14.1)	3.18(14.0)	4.63(5.9)	4.64(20.9)	2.65(18.4)	2.50(13.6)	2.80(3.2)	4.45(10.8)	5.63(13.8)
Q04857	Collagen alpha-1(VI) chain	Col6a1	2.34(31.1)	4.74(21.1)	6.26(12.8)	4.46(14.2)	4.68(8.4)	4.03(21.8)	2.82(20.9)	3.51(18.9)	3.14(6.2)	7.32(17.7)
Q60847	Collagen alpha-1(XII) chain	Col12a1	3.00(4.8)	2.33(4.0)	5.70(17.6)	5.41(0.0)	7.41(0.1)	2.54(20.4)	4.15(10.3)	7.13(20.8)	5.98(10.1)	9.29(10.2)
Q80X19	Collagen alpha-1(XIV) chain	Col14a1	4.04(10.0)	5.32(16.3)	7.21(11.4)	7.68(2.9)	8.23(9.5)	6.63(19.4)	6.94(19.1)	5.43(11.7)	7.01(2.8)	9.08(6.5)
O35206	Collagen alpha-1(XV) chain	Col15a1	1.55(20.4)	1.42(14.1)	2.68(16.4)	1.60(10.2)	1.72(5.8)	3.63(11.1)	1.04(17.5)	3.51(10.9)	2.49(7.0)	4.06(19.7)
Q07563	Collagen alpha-1(XVII) chain	Col17a1						0.17(17.6)	1.16(21.1)	0.89(11.2)	1.32(15.2)	1.39(14.4)
P39061	Collagen alpha-1(XVIII) chain	Col18a1	3.04(19.7)	(0.0)	2.74(18.3)	1.60(10.2)	2.62(19.1)	3.92(12.2)	1.40(14.3)	4.00(2.3)	2.33(22.1)	3.87(15.7)
Q01149	Collagen alpha-2(I) chain	Col1a2	4.29(8.0)	2.62(14.8)	4.04(0.5)	5.07(14.9)	6.36(8.8)	4.07(16.5)	4.70(4.5)	2.96(16.5)	4.14(16.5)	7.71(8.0)
Q02788	Collagen alpha-2(VI) chain	Col6a2	4.92(21.5)	3.74(13.4)	3.93(17.5)	4.29(19.3)	3.76(2.3)	2.75(20.8)	1.95(17.8)	1.01(11.6)	2.99(22.6)	6.62(21.0)
Q9D1D6	Collagen triple helix repeat-containing protein 1	Cthrc1	0.83(12.1)	1.64(18.3)	2.39(20.5)	3.09(7.4)	4.44(12.8)	2.14(18.7)	1.16(8.6)	0.89(16.9)	1.35(22.2)	3.47(6.1)
Q80YX1	Tenascin	Tnc		3.05(13.6)	6.86(18.4)	8.73(2.0)	8.74(4.1)	2.07(19.9)	5.80(24.3)	7.01(21.7)	7.43(5.4)	8.25(2.7)
**Complement and Coagulation Factors**												
P08607	C4b-binding protein	C4bp							4.63(4.2)	3.57(5.0)	4.49(6.3)	2.39(20.9)
O88947	Coagulation factor X	F10		3.28(13.5)	3.82(14.2)	1.60(14.4)	1.38(21.8)	1.56(19.2)	4.81(19.6)	2.47(19.5)	4.98(15.5)	1.23(7.5)
Q80YC5	Coagulation factor XII	F12							3.24(21.0)	2.30(10.8)	3.67(16.3)	1.28(12.1)
Q8CG14	Complement C1s-A subcomponent	C1s							3.55(16.7)	2.30(10.7)	4.50(11.4)	2.28(55.3)
P21180	Complement C2	C2	1.27(15.7)	2.14(18.6)	1.87(13.3)	2.59(17.7)	1.38(21.8)		5.10(17.9)	2.36(8.6)	4.19(5.9)	1.28(12.1)
P01027	Complement C3	C3	3.99(8.5)	8.52(11.7)	8.20(4.9)	8.61(8.1)	7.36(3.9)	7.57(2.4)	11.07(1.9)	10.64(1.7)	11.05(2.5)	8.22(2.2)
Q8K182	Complement component C8 alpha chain	C8a							6.39(5.5)	4.02(14.0)	3.14(6.2)	3.36(25.6)
Q8VCG4	Complement component C8 gamma chain	C8g						0.58(17.2)	5.10(20.0)	2.30(17.8)	4.34(13.4)	1.39(14.4)
P06683	Complement component C9	C9						0.38(14.8)	6.04(10.3)	4.76(5.5)	4.98(15.8)	3.78(16.2)
P03953	Complement factor D	Cfd						0.36(12.1)	1.69(19.5)	2.69(10.8)	2.86(15.5)	1.29(8.6)
Q61129	Complement factor I	Cfi	1.05(21.3)	4.20(16.7)	2.89(7.5)	4.12(14.9)	2.39(19.7)	0.17(11.8)	5.18(0.5)	4.88(10.6)	5.48(11.0)	1.28(8.6)
E9PV24	Fibrinogen alpha chain	Fga	3.05(7.3)	3.83(15.0)	4.48(3.4)	4.58(22.4)	5.16(12.8)	5.08(17.0)	6.54(0.6)	6.98(2.6)	7.86(5.8)	5.93(19.7)
Q8K0E8	Fibrinogen beta chain	Fgb	3.97(15.9)	7.28(19.4)	7.81(4.2)	8.34(11.7)	7.79(3.0)	5.91(16.2)	7.67(5.9)	8.12(7.8)	8.36(4.6)	6.59(2.5)
Q8VCM7	Fibrinogen gamma chain	Fgg	5.51(18.1)	6.21(19.9)	6.69(5.4)	7.48(11.6)	6.48(7.3)	5.97(17.4)	7.74(5.3)	8.04(9.0)	8.56(4.4)	6.43(5.3)
P20918	Plasminogen	Plg	4.40(18.4)	8.01(5.2)	7.53(11.6)	8.46(11.8)	6.76(15.4)	4.91(11.7)	9.34(2.1)	9.19(1.8)	9.65(2.0)	5.69(19.7)
P19221	Prothrombin	F2	2.73(20.8)	6.40(16.6)	6.08(11.8)	6.61(17.7)	5.26(7.3)	2.81(16.7)	7.39(8.9)	7.07(5.4)	8.15(3.3)	5.52(0.9)
**Immunomodulators**												
P08071	Lactotransferrin	Ltf	1.04(19.2)	7.58(3.5)	6.91(6.1)	7.57(9.7)	6.49(0.4)	3.39(16.9)	9.88(6.2)	9.70(6.4)	9.63(5.0)	5.94(21.5)
P11247	Myeloperoxidase	Mpo	1.04(19.2)	6.70(15.2)	6.48(9.4)	6.42(16.4)	5.04(9.7)	3.17(18.9)	7.62(5.8)	7.52(6.9)	7.69(7.2)	3.13(14.7)
P50543	Protein S100-A11	S100a11		2.33(17.4)	2.37(11.6)	3.26(6.4)	2.82(17.7)	3.73(18.2)	4.53(7.0)	5.78(6.1)	4.28(13.2)	4.39(14.5)
P14069	Protein S100-A6	S100a6						2.10(21.1)	1.54(17.5)	2.67(20.4)	2.87(18.2)	2.73(15.9)
P27005	Protein S100-A8	S100a8							6.08(13.7)	8.81(11.9)	4.57(12.4)	2.17(9.2)
P31725	Protein S100-A9	S100a9							7.76(10.2)	8.15(10.3)	7.48(13.0)	2.03(17.7)
**Macropage Markers**												
O08691	Arginase-2, mitochondrial	Arg2		1.83(22.2)	2.04(14.7)	2.10(17.3)	1.61(13.8)					
Q61176	Arginase-1	Arg1						1.38(21.1)	6.07(11.3)	5.63(9.2)	5.95(7.6)	4.52(16.7)
Q61830	Macrophage mannose receptor 1	Mrc1	1.55(20.4)	3.30(13.4)	5.25(7.5)	4.33(1.9)	4.38(18.7)	5.04(15.8)	5.33(16.9)	5.43(9.2)	5.05(17.2)	6.79(8.8)
Q64449	C-type mannose receptor 2	Mrc2	1.27(15.7)	1.42(14.1)	1.89(8.1)	3.31(15.3)	4.00(22.0)	2.00(18.4)	2.45(2.7)	3.33(3.5)	3.94(9.7)	4.65(16.8)
**Others**												
O70456	14-3-3 protein sigma	Sfn	5.69(5.2)	6.61(8.1)	7.13(2.9)	7.19(3.8)	6.73(4.8)	6.42(14.9)	5.09(15.1)	5.56(4.3)	6.18(2.0)	6.30(10.3)
P63101	14-3-3 protein zeta/delta	Ywhaz	5.88(5.0)	6.69(10.8)	7.29(6.1)	7.36(2.7)	6.80(2.8)	6.81(10.5)	5.82(18.7)	6.59(1.1)	6.72(5.4)	6.62(9.9)
P62737	Actin, aortic smooth muscle	Acta2	10.82(2.8)	10.79(18.5)	11.19(5.2)	10.56(1.2)	10.79(5.3)	10.22(10.5)	10.31(2.6)	9.92(1.4)	10.00(15.0)	9.37(2.3)
Q9WV32	Actin-related protein 2/3 complex subunit 1B	Arpc1b	2.16(7.5)	4.94(19.1)	4.85(9.5)	4.84(7.5)	3.97(14.4)	2.30(16.3)	4.42(1.4)	4.14(15.4)	4.55(8.9)	3.68(18.8)
Q9JM76	Actin-related protein 2/3 complex subunit 3	Arpc3	1.05(21.3)	5.18(9.9)	5.32(0.8)	4.56(7.8)	4.59(12.4)	3.73(14.9)	5.31(7.4)	4.36(14.0)	4.61(20.8)	3.61(18.5)
Q91V92	ATP-citrate synthase	Acly	4.82(19.4)	3.22(16.0)	5.52(12.2)	5.22(15.4)	5.30(19.6)	5.76(15.7)	4.05(13.3)	4.62(3.8)	4.34(22.9)	6.42(8.8)
P26231	Catenin alpha-1	Ctnna1	3.72(20.6)	1.64(13.4)	3.89(21.8)	4.51(17.4)	5.02(17.2)	5.33(11.6)	1.16(21.1)	3.96(12.3)	4.15(14.4)	6.23(5.1)
Q61301	Catenin alpha-2	Ctnna2	2.82(19.4)	5.08(20.6)	3.39(10.2)	3.73(16.4)	3.89(12.5)	3.59(21.5)	1.16(21.1)	1.80(22.7)	2.35(21.3)	4.32(14.6)
Q9CZ13	Cytochrome b-c1 complex subunit 1, mitochondrial	Uqcrc1	2.45(10.3)	3.80(10.0)	1.89(8.1)	2.10(14.3)	2.29(18.1)	4.51(22.4)	3.66(12.5)	4.31(8.9)	3.88(16.1)	4.37(9.4)
Q00612	Glucose-6-phosphate 1-dehydrogenase X	G6pdx	3.30(12.2)	5.91(18.2)	6.00(7.0)	5.46(19.7)	4.86(1.9)	3.51(21.6)	6.42(10.4)	5.75(17.6)	5.54(13.6)	3.78(16.5)
P63017	Heat shock cognate 71 kDa protein	Hspa8	7.55(13.6)	7.99(5.6)	8.01(8.0)	8.41(3.7)	8.41(3.9)	8.02(2.5)	7.85(6.4)	7.75(7.7)	7.40(6.1)	8.49(2.6)
P09055	Integrin beta-1	Itgb1	1.55(20.4)	3.96(5.5)	4.62(12.5)	4.93(11.1)	4.85(22.8)	4.31(3.9)	3.84(18.2)	4.15(22.9)	3.30(18.8)	4.98(17.5)
O70309	Integrin beta-5	Itgb5		2.05(12.9)	3.82(7.1)	3.26(6.4)	2.10(10.6)		3.42(18.1)	3.02(12.7)	3.05(17.7)	2.57(15.6)
Q91WD5	NADH dehydrogenase [ubiquinone] iron-sulfur protein 2, mitochondrial	Ndufs2	3.24(16.8)	2.14(13.1)	2.51(12.5)	2.10(16.0)	2.90(18.1)	2.74(14.4)	1.66(14.8)	1.01(16.4)	2.35(21.3)	3.44(7.9)
Q9DCT2	NADH dehydrogenase [ubiquinone] iron-sulfur protein 3, mitochondrial	Ndufs3	3.02(20.1)	3.74(16.0)	1.82(10.5)	1.60(10.2)	1.60(13.9)	3.62(17.9)	2.04(8.9)	1.30(18.9)	1.33(1.1)	2.68(12.8)
O35468	Protein Wnt-9b	Wnt9b	1.04(19.2)	1.86(16.1)	2.04(14.7)	2.83(14.1)						
P63001	Ras-related C3 botulinum toxin substrate 1	Rac1	2.34(22.0)	4.71(22.6)	5.40(7.5)	4.84(16.0)	3.77(17.2)	2.80(21.7)	4.62(0.3)	2.96(16.2)	3.87(19.1)	3.57(15.9)
Q8K2B3	Succinate dehydrogenase [ubiquinone] flavoprotein subunit, mitochondrial	Sdha	4.08(14.4)	3.30(18.9)	2.82(6.8)	3.42(0.4)	2.60(12.1)	5.05(16.5)	3.04(13.5)	2.59(6.4)	2.33(22.1)	4.29(21.2)
Q93092	Transaldolase	Taldo1	4.45(18.8)	6.53(14.4)	6.83(10.8)	6.33(12.3)	5.38(6.8)	4.77(5.2)	6.05(8.4)	5.62(5.1)	5.88(2.0)	5.54(3.5)
Q9QUI0	Transforming protein RhoA	Rhoa	1.55(14.4)	4.37(18.9)	5.20(9.1)	6.02(5.5)	3.77(13.2)	3.93(16.2)	4.50(0.1)	3.99(2.2)	3.96(15.6)	4.21(14.6)
Q9D4D4	Transketolase-like protein 2	Tktl2	2.04(14.7)	2.64(11.7)	2.89(15.4)	3.51(16.5)	1.82(16.2)	1.21(20.1)	3.13(17.7)	2.34(22.4)	2.74(8.2)	2.86(5.4)
P20152	Vimentin	Vim	7.84(13.6)	8.68(0.7)	9.37(2.1)	9.50(5.2)	10.30(6.3)	8.75(4.4)	8.37(9.9)	8.73(6.3)	8.33(8.4)	9.51(5.7)

^*^Quantitative value is log2(protein area/total protein area) ×10^6^.

### Global protein profiling between *Acomys* and *Mus* skin over 14 days after full thickness skin wounding

**Figure 3:**
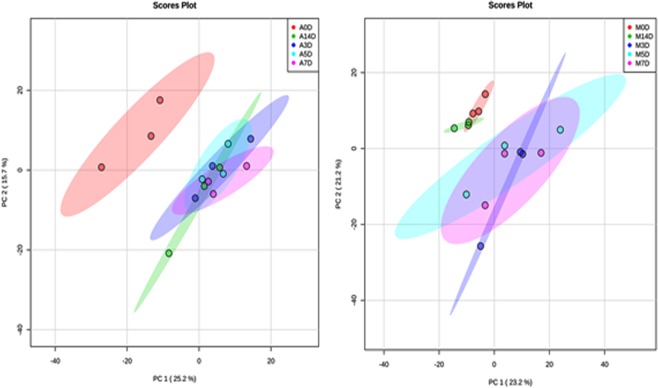
Principal components analysis (PCA) of the expression profiles of common proteins detected for skin samples from *Acomys* (left plot) and *Mus* (right plot) indicating red dots for day 0, dark blue dots for day 3, pale blue dots for day 5, pink dots for day 7 and green dots for day 14. Three replicates at day 0, 3, 5, 7, and 14 are delineated with the ellipses.

We first compared the protein expression profiles to assess the trends between the two species during wound healing by a principal component analysis (PCA) of the common proteins (a total of 1545). This showed differences in the overall expression profiles between *Acomys* and *Mus* through 14 days (Fig. 3). Interestingly, the protein expression levels in *Acomys* from 0 to 3 days were clearly separated but, at 5, 7 and 14 days were clustered closely. Correspondingly, *Mus* at 0 day was well separated, at 3, 5, and 7 days clustering was apparent but not at 14 days which unexpectedly showed a return to the day 0 profile. This suggests that by day 14 the *Mus* skin has effectively completed its scarring process, but the *Acomys* skin is still in the process of new protein production and regeneration. To assess the statistical significance associated with biological variation from three biological replicates at the different time points, the coefficient of variations (CV) were determined and presented in the supplemental Fig. S3. This showed the high reproducibility across all samples.

**Figure 4:**
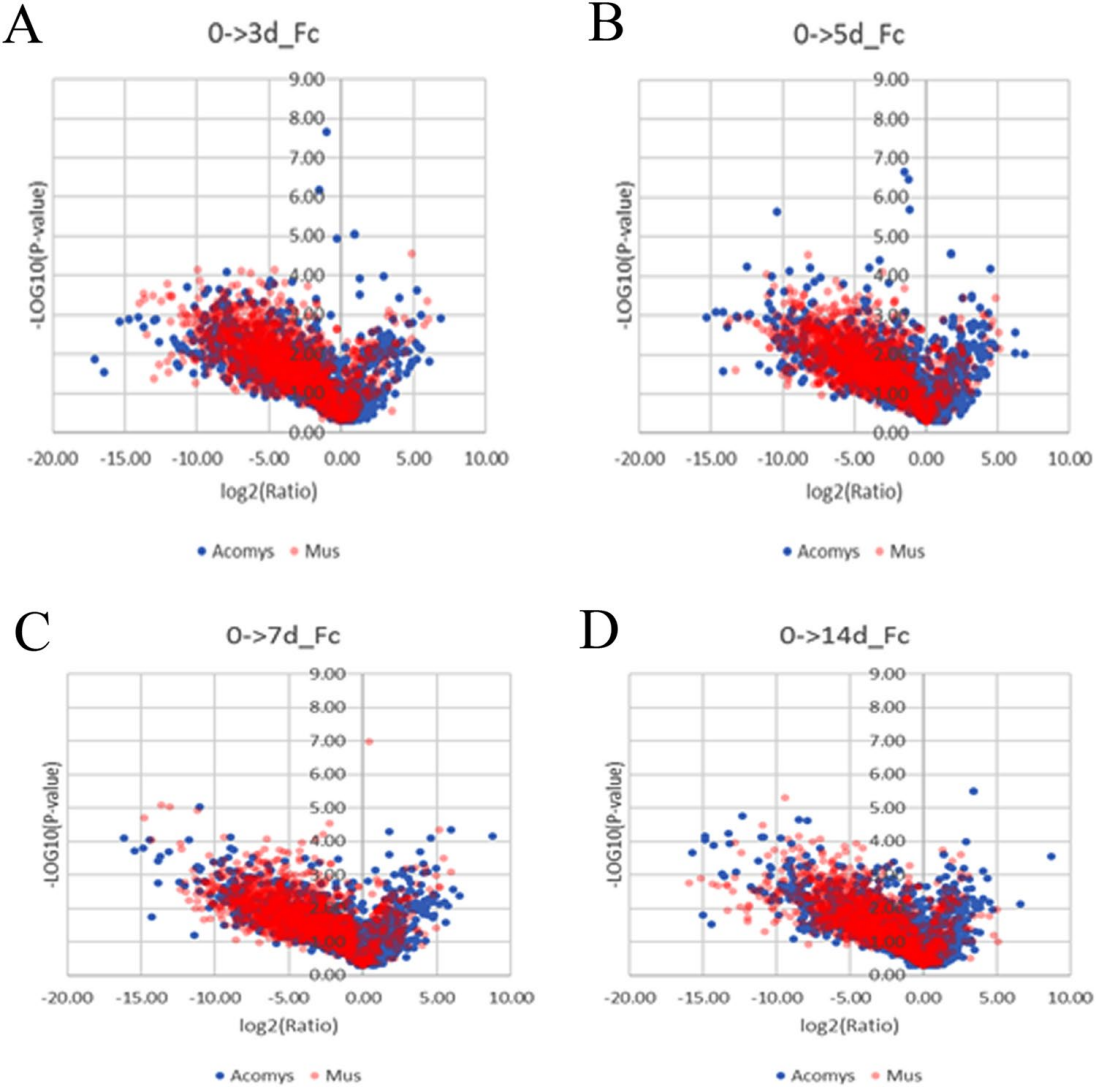
Volcano plots comparing fold changes of common proteins between *Acomys* and *Mus* over periods 0-3d (3A), 0-5d (3B), 0-7d (3C) and 0-14d (3D). The colors indicate the protein source, *Acomys* blue and *Mus* pink.

We next compared the global changes of the common proteins between *Acomys* and *Mus* over 14 days (see Fig. 4A–D). This showed no clear changes over the time periods, but common proteins were highly biased in *Mus* towards negative fold changes. This was not the case in *Acomys* where the fold changes were more evenly distributed than *Mus* between positive and negative.

### KEGG analysis of the differentially expressed proteins in *Acomys* and *Mus* skin during wound healing

**Figure 5:**
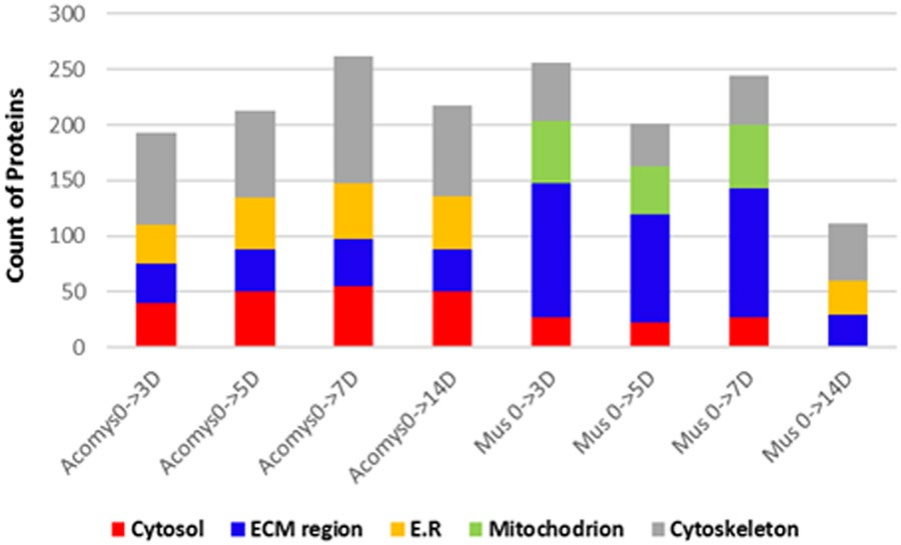
Gene ontology analyses of significantly changed (p <0.01) differentially expressed proteins (DEPs) by subcellular location in *Acomys* and *Mus* at day 0.

Proteins were considered as differentially expressed in *Acomys* and *Mus* if they exhibited a fold change during the four time periods of >1.6 or <-2 with p- value < 0.01 among the biological replicates. When we analyzed the counts of differentially expressed proteins (DEPs) in the two species in terms of their cell locations, despite being similar in the normal skin at day 0 (Fig. 2) it was apparent that during regeneration/scarring differences appeared (Fig. 5). Whereas the DEPs in *Acomys* localized to cytosol, extracellular matrix, ER and cytoskeletal components, DEPs in *Mus* were far more strongly localized to the extracellular matrix and mitochondrial components. This suggests that these two cellular locations characterize *Mus* scarring and the more regenerative phenotype of *Acomys* is characterized by the ER and higher cytoskeletal and cytosolic representations.

**Figure 6:**
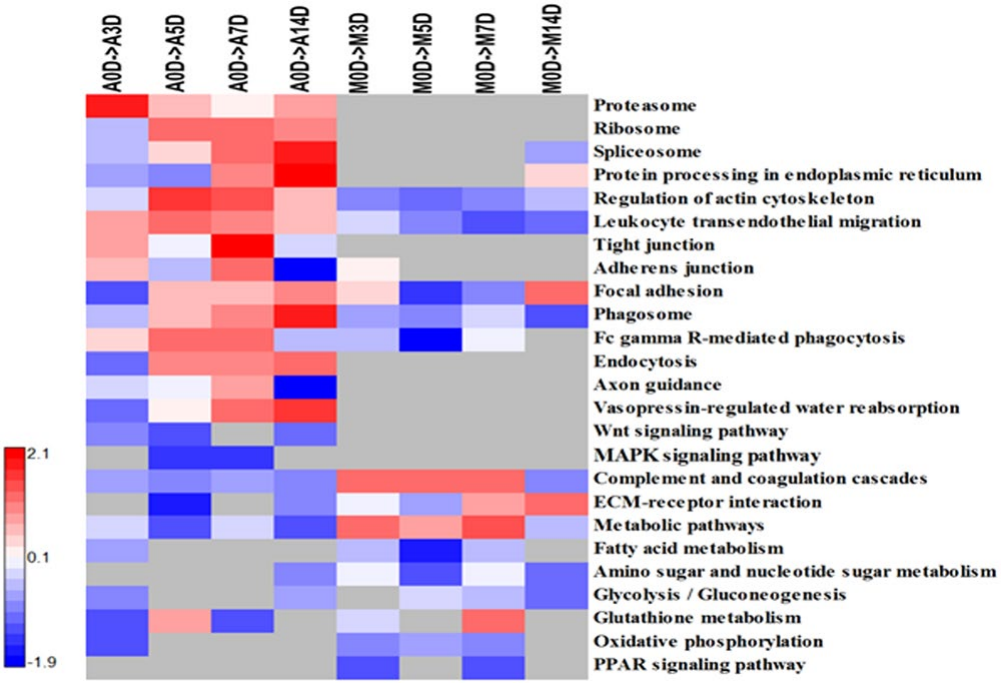
Heat-map shows the results of a pathway analysis related to GO biological process terms for DEPs between *Acomys* (A) and *Mus* (M) at different time periods of 0, 3, 5, 7, 14 days, (*p* < 0.01). The scaling bar indicates protein fold changes ranging from −2 to +2.1.

To obtain further insight into the functional roles for DEPs associated with wound healing in *Acomys* and *Mus*, we carried out GO-BP and KEGG pathway analyses. We first made pathway annotations based on the KEGG database for both positive and negative DEPs. Twenty- five categories were enriched (Fig. 6), among which fourteen were particularly prominent, in *Acomys*. On the other hand, nine were enriched in *Mus* with only three prominent, the hub proteins enriched in pathways in our study are given in Table S3. The most notably enriched categories in *Acomys* were those involved in tight junction formation, endocytosis, the ribosome pathway, the proteasome pathway, Wnt signaling, MAPK signaling and vasopressin-regulated water reabsorption. In contrast, there was little enrichment of pathways in *Mus* overall, except for the complement and coagulation cascades, PPAR signaling, (Table S3) ECM-receptor interactions and metabolic pathways (Fig. 6).

**Figure 7:**
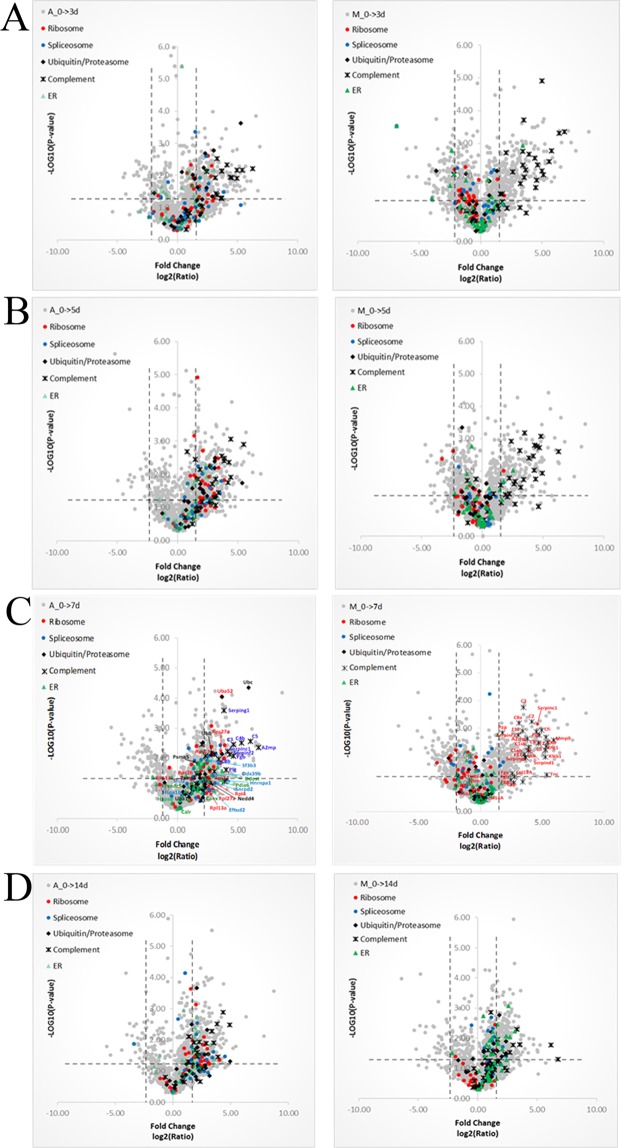
Volcano plots showing fold changes of relative protein abundances for *Acomys* and *Mus* over periods 0–3d (**A**), 0–5d (**B**), 0–7d (**C**) and 0–14d (**D**). The proteins highlighted refer to processes/pathways related to protein degradation/ synthesis and inflammatory response. viz. red dots for ribosome, blue dots for spliceosome, green triangle for protein processing in E.R, black diamond for ubiquitin/proteasome and black cross for complement and coagulation cascades.

The fold changes of levels of proteins representative of five different pathways during different times from day 0 for *Acomys* and *Mus* are shown as volcano plots in Fig. 7A–D to show the changes over time rather than a static plot. These figures showed the trends of DEPs changes at different time points.

Ribosomal proteins showed clearly different behavior, being elevated in *Acomys* but depressed in *Mus*. Spliceosome proteins were elevated in *Acomys* through 14 days, but in *Mus* were initially (0–7d) little changed and then elevated after 14 days. The ubiquitin/proteasome pathways were elevated in *Acomys* throughout all time points. The complement proteins are elevated in both species but there are more representatives in *Mus* and the ER components show changes in both *Acomys* and *Mus* but at different time points.

### Regulatory pathways of protein degradation /synthesis in *Acomys*

Ubiquitin-proteasome pathway (UPP) degradation mechanisms were highly activated in *Acomys* compared to those in *Mus*. Thus UBB, UBC, NEDD4, UBA1, and UBA52 which were detected in both species increased at day 7 in *Acomys*, but those in *Mus* were almost unchanged. Several of this class of proteins, namely UBE2E1, UBE2O, UBE2Q2, USP7, USP34 and RNF40 were only detected in *Acomys* (Table 1).

26S proteases having unfoldase function (PSMC3, PSMA5) showed significant increases in *Acomys*. Several proteins in ribosome pathways, especially 40S ribosomal proteins (S13, S17, S26, S27A) and 60S (L4, L13A, L27A) were identified in both species (Table 1). Most of them showed significant increase in *Acomys*, but not in *Mus*. We also observed DEPs in the spliceosome pathway such as SF3B3, SRSF2, SNRPD2, HNRNPA1, DDX39B and EFTUD2 which in *Acomys* showed a characteristic pattern of low level expression at day 0 rising to high levels by day whereas in *Mus* their levels were almost unchanged or slightly decreased over the same time period.

Proteins in the ER, especially two lectins CALR and CANX which are involved in the CNX/CRT cycle for phosphorylation, showed significant increase in *Acomys*. DDOST, PDIA6 and TXNDC5 were also elevated in *Acomys* to a higher degree than in *Mus*.

Of the 12 identified proteins involved in phosphorylation in *Acomys* (cell cycle proteins, growth factor receptors, tyrosine protein kinases) they generally did not increase significantly over time, but most strikingly only 3 of them (EFGR, EPS15 and WNK1) were identified in *Mus*. Representative DEPs enriched in these pathways in *A. cahirinus* are presented in Table 1.

### Profiles of intermediate filaments (IFs) in *Acomys* and *Mus*

Keratins were highly abundant in both species and twenty-three are listed in Table S4. The amounts of various cytoskeleton keratins showed little change through 14 days with a couple of exceptions, but cuticular keratins (K31, K32, K33b, K35 and K36) were significantly higher in *Acomys* compared to *Mus* at day 0, subsequently showing fairly constant values over 14 days (Table 1). Vimentin, a fibroblast marker prominent in both species, was 1.5-fold elevated in *Acomys* from day 0 to day 7, and 2.5-fold upregulated at day 14 whereas *Mus* showed almost unchanged levels at the same time period (Table 1). Interestingly the only Wnt detected was Wnt 9b in *Acomys*. The small GTPases RhoA and Rac1 were detected at significantly higher levels in *Acomys* at most time points (Table 1).

### Regulatory pathways of immune/inflammatory responses in *Mus*

Innate immune response-related proteins, especially those related to the complement pathway, were up-regulated in *Mus*. 16 complement and coagulation factors and 6 immuno-modulatory proteins were detected in *Mus*, with many showing temporary increases over 3-7 days before decreasing at day 14. Exclusive detection of complement and coagulation factors (C1S, C4BP, C8A, C8G, C9, CFD, and F12) as well as significantly elevated levels of C2, C3, CFI, FGA, FGB, FGG, F2, F10 and PLG were observed in *Mus*. Of these 22 proteins 10 were absent from *Acomys* extracts and the temporary increases in levels was again apparent but less pronounced (Table 1).

Myeloperoxidase as a neutrophil marker was highly increased in both species. However, arginase 1 was only identified in *Mus* and arginase 2 only in *Acomys* (Table 1). 10 proteases were identified and showed very different levels of expression between *Acomys* and *Mus*. Cathepsin G, mast cell protease 4 and tryptase beta 2 were only expressed in *Acomys*, whereas matrix metalloprotease 9, plasma kallikrein and pro-cathepsin H and cathepsin L1 were solely detected in *Mus* (Table 1). Cathepsins B, D, and Z were expressed in both species, they were at constant high levels in *Mus* but increased through 14 days in *Acomys*. A total of 17 serine protease inhibitors (SERPINs) were quantified in our study (Table 1 and S5). Strikingly, the vast majority were not detected in *Acomys* at any stage, for example only 2 of the 13 listed in Table S5 were identified in *Acomys*

### Collagenous composition in extra cellular matrix (ECM)

Next, we investigated collagen levels in the two species and 10 collagens, all alpha isoforms, were identified. Only 1 could not be detected in *Acomys*, COL 17A1, and the remainder increased throughout the 14 days period in both species, but with a greater increase seen in *Mus*. The largest increase in *Mus* over *Acomys* was seen in COL12A1. The collagen triple helix repeat-containing protein 1 which is involved in collagen remodeling showed far higher levels in *Acomys* (Table 1).

### Verification of fibrotic/non-fibrotic related proteins by western blot analysis

**Figure 8:**
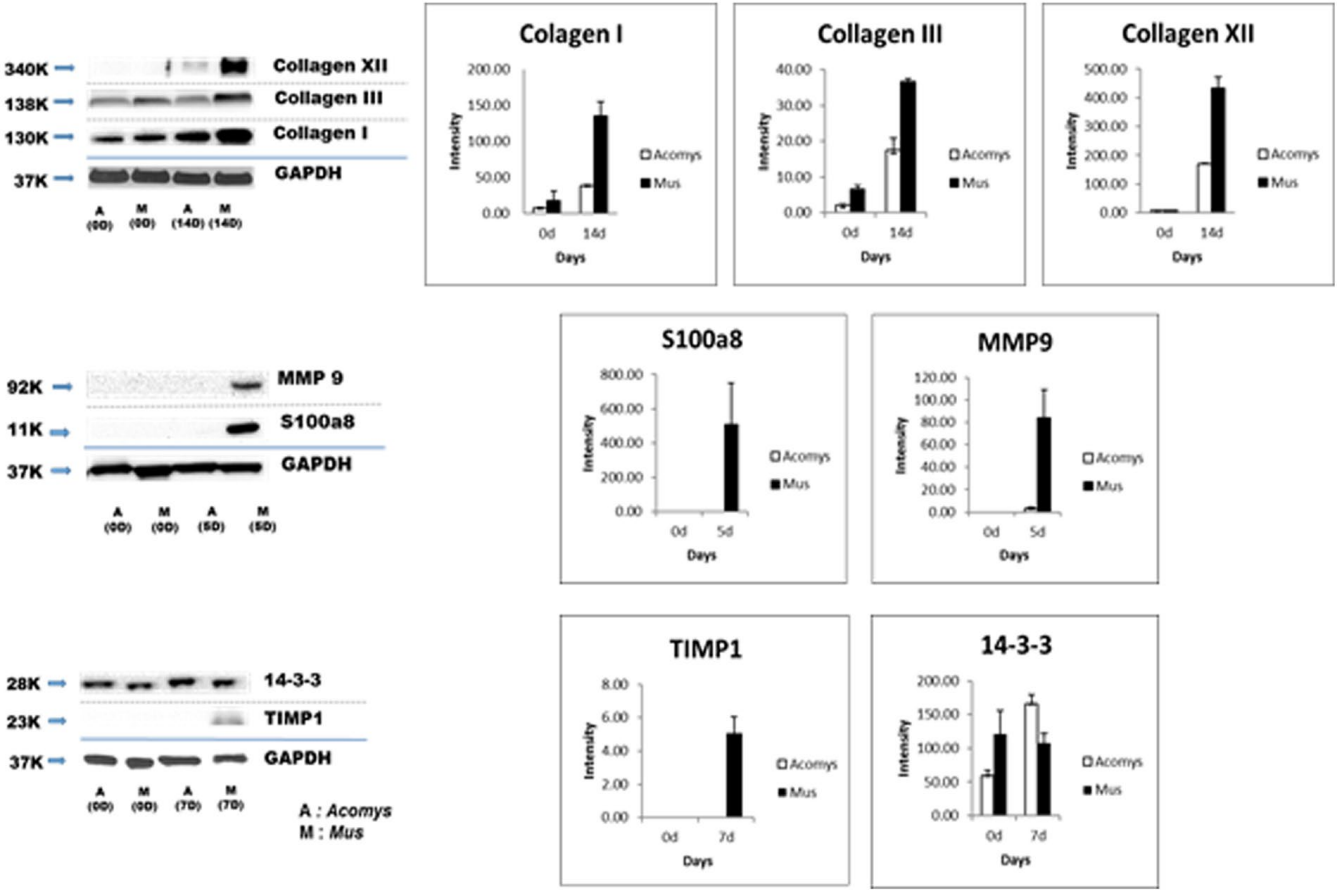
Western blots of skin and wound lysates, with GAPDH as a loading control and detection by specific antibodies. Lane designations A, M, refer to *Acomys* and *Mus*, figures in brackets are days post-wounding; error bars on intensity values are standard deviations.

Since they are of particular interest due to their involvement in immune defense and ECM synthesis, we verified the expression of COL1, COL3, COL12, S1008, MMP9, TIMP and 14-3-3 by western blot analysis (Fig. 8). Wound collagens at day 14 revealed significant changes compared to day 0. COL1, COL3 and COL12 were 7.6 times, 5.5 times and 58 times increased in *Mus* and 5.7 times, 7.8 times and 21 times increased in *Acomys* respectively, confirming the generally higher levels in *Mus* that the proteomic analysis recorded (Table 1). S100A8 and MMP9 at day 5 and TIMP at day 7 were detected only in *Mus*. 14-3-3 δ at day 7 was increased 2.8 times in *Acomys* and less than 1 in *Mus*.

### Protein -protein interaction analysis

**Figure 9:**
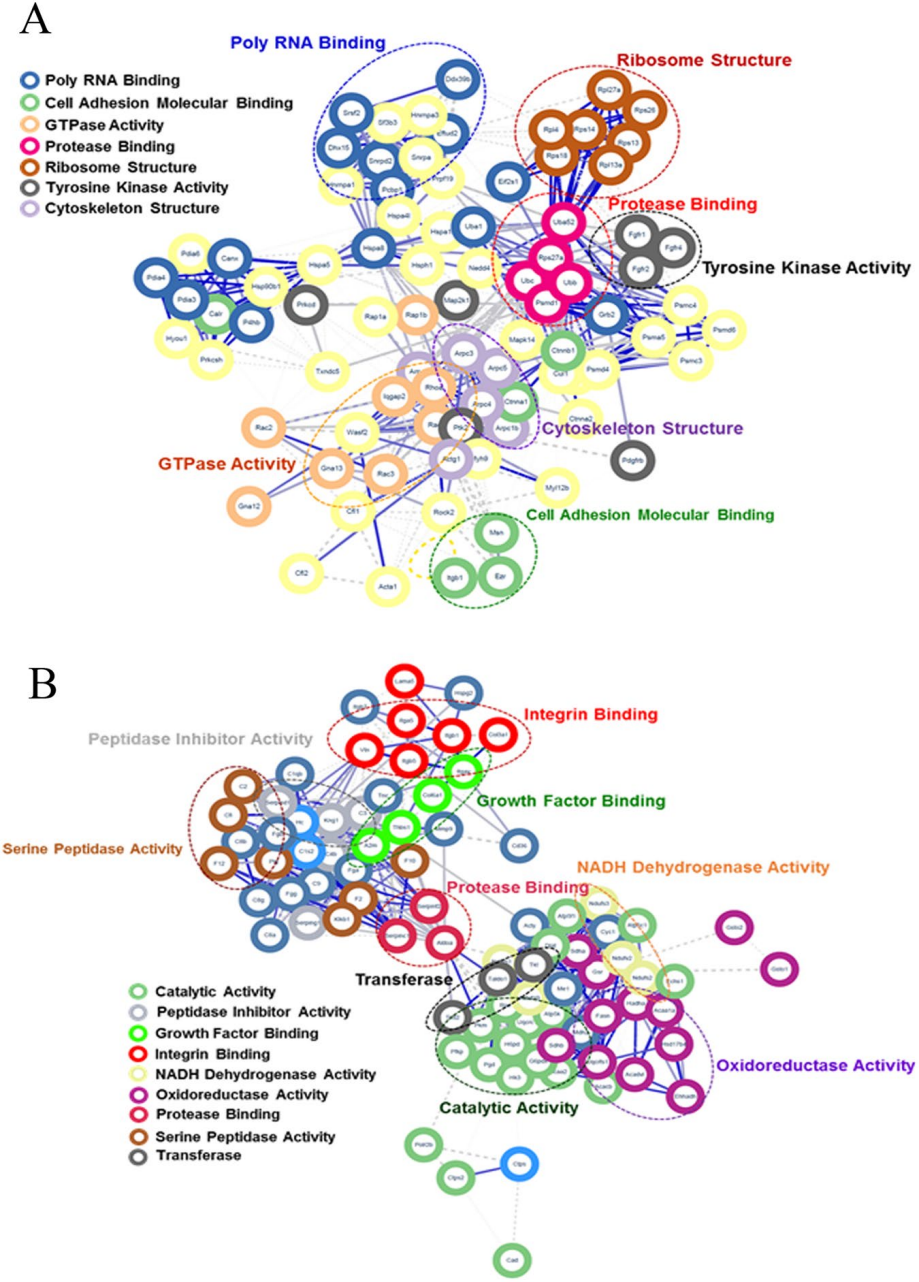
Protein-protein interactions predicted for DEPs found in *Acomys* and *Mus*. The STRING database was used to examine proteins significantly changed in *Acomys* (**A**) or in *Mus* (**B**). Each node in the network represents a DEP. Interactions are shown by the blue lines connecting each node with the weight of each line representing the confidence of the interaction based on available evidence in the database. Clusters of interest are indicated by the colored labels.

To examine the interactions between DEPs detected in response to wound healing in *Acomys* and *Mus*, we constructed network models using DEPs from the STRING database (http://string-db.org) (Fig. 9A,B). From this map, we searched the key proteins and their interactions involved in biological processes that may influence the wound healing outcomes. Overall, several hub proteins exhibiting physical and co-expression interactions with multiple proteins in diverse pathways were identified in the two species. For *Acomys* (Fig. 9A) the number of smaller interaction groups were identified and consisted of cell signaling and protein degradation and synthesis pathway components. These included protein tyrosine kinase activities such as MAP2K1 and FGFR4, GTPase activity (RAC1, RHOA), cell adhesion (CTNNB1, Calr), protease binding (UBA52, S27A), RNA binding (HAPA8, EFTUD2), cytoskeletal structure (ARPC3, ARPC1B), and ribosome structure (L27A, S13) (Fig. 9A). In *Mus*, the interacting groups included integrin binding which would be associated with the higher levels of collagens that we detected, catalytic activity (ACAA2, UQCRC1), oxidoreductase activity (SDHA, GSR), NADH dehydrogenase (NDUFS2 and NDUFS3), serine peptidase inhibitor (KLKB1, F2), growth factor binding (COL6A1, ITGAV), peptidase inhibitor activity (KNG1, SERPIND1), protease binding (SERPINC1, SERPINF2) and transferase activity (TKT, TALDO1) (Fig. 9B). Additional DEPs not exhibiting interactions with other proteins found in this analysis also likely play indirect roles in wound healing for *Acomys* and *Mus*.

## Discussion

*Acomys cahirinus* retains a remarkable capacity for regenerative healing potential in a scar- free manner across multiple adult tissues. To reveal the mechanisms that support regeneration of *Acomys* on a molecular level we have undertaken a qualitative and quantitative proteomic analysis of *Acomys* compared to the scarring *Mus* using LC-MS/MS approaches. We identified proteins and their expression levels from skin tissue extracts with label free quantification through 0, 3, 5, 7 and 14 days as the wounds are undergoing re-epithelialization, the inflammatory phase and establishing granulation tissue. These stages were chosen as key time points for identifying protein differences potentially responsible for regeneration vs scarring and to correlate with our previous cellular and molecular studies^17,18^. The quantitative results of several proteins from skin wounds in this data were also validated by western blot analysis and demonstrate a good correlation of protein levels between our mass spectrometric based methodology and immunoblotting.

Re-epithelialization and histolysis are key early events required for the ability to regenerate complex tissues. Our proteomics studies revealed that the most enriched pathway and proteins among the DEPs were categorized as protein degradation/synthesis, especially, with the highly enriched ubiquitin/proteasome pathway present in *Acomys*. Accordingly, UBA, UBC and NEDD4 as ubiquitin enzymes are highly activated in *Acomys*, resulting in tagging and directing target/condemned protein into the proteasome, where they are degraded and recycled. These degraded proteins activate other proteins that contribute to cell cycle and cell survival mechanisms. A recent study demonstrates highly enriched ubiquitination/proteasomal degradation in liver regeneration, especially NEDD4 as an essential regulator by performing EPS15 ubiquitination which promotes EGFR internalization and efficient signaling in hepatocytes^21^. We were able to detect EGFR and EPS15 after wounding with a 2.7-fold increased NEDD4 at day7 in *Acomys* but EGFR and NEDD4 were either constant or significantly decreased in *Mus*, with little EPS15.

Although both *Mus* and *Acomys* can re-epithelialize their skin wounds the former does not normally replace the hairs and so the repaired skin remains hairless unless large wounds are made in some *Mus* strains, so called wound induced hair follicle neogenesis^13^. *Acomys* on the other hand regenerates all the hairs beginning as early as day 11 after wounding. The Wnt pathway plays a role in controlling epidermal stem cell renewal, in reciprocal interactions with the dermal papilla requirement and activation of the migrating wound epidermis^22–25^. Hair follicle induction specifically involves *Wnt7a* as ectopic expression of this gene in *Mus* induced hair follicle neogenesis^26^ and *Wnt7a* expression is specifically up-regulated in *Acomys* wounds^17^. The only Wnt that we detected here was Wnt9, only in *Acomys* (Table 1) and its levels increased 2-fold on day 7. However, many members of the Wnt pathway were identified as up-regulated in *Acomys* following a KEGG analysis confirming the role of this pathway in hair follicle regeneration.

Another feature of *Acomys* re-epithelialization is a 2x faster rate of cell migration^11,27^. We identified many keratins in this proteomic analysis, the majority of which showed no differences between *Acomys* and *Mus* except for some cuticular keratins such as K31, K32, K35 and K36 which were elevated in *Acomys*. We have also observed similar expression levels of K17, the embryonic keratin involved in follicle neogenesis but a 2 times higher expression of 14-3-3δ as well as strongly increased vimentin levels in *Acomys*. Since an interaction of keratin 17 with 14-3-3δ and with signaling proteins for the regulation of cell growth in wounds^28^ and a vimentin interaction with ribosomal protein S17 and 4-3-3δ for stimulating protein synthesis^29^ have been demonstrated, it is perfectly feasible that increased levels of intermediates such as 14-3-3 δ in *Acomys* could have significant stimulatory effects on regeneration. Furthermore, keratins can profoundly influence cytoarchitecture by regulating signaling pathways and modulating protein synthesis and cell growth during the process of tissue repair^30^ so these particular proteins may also be having some influence on the rate of re-epithelialization if not more profound effects on regeneration after injury in *Acomys*.

We know from previous molecular and cellular analyses that the immune and macrophage responses to injury are quite different between *Acomys* and *Mus*. In skin regeneration there is a huge immune cytokine response in *Mus* and M1 macrophages (F4/80) are present throughout the resolving granulation tissue after wounding whereas in *Acomys* the immune response is blunted and M1 macrophages are absent or deficient from an equivalent region^18,31^. The regenerating *Mus* ear cytokines are expressed at higher levels than in *Acomys* and similarly, the ear is nearly devoid of classically activated macrophages (CD86) but shows plenty of M2, CD206 macrophages^19^. The same is seen during skeletal muscle regeneration and kidney regeneration in *Acomys*^32,33^. Nevertheless macrophages are necessary for epimorphic regeneration^19^ so the M2 phenotype must provide these pro-regenerative cytokines. We have found that to be the case here using proteomics. Thus, several immune modulators including S100A6, S100A8 and S100A9 were detected only in *Mus*. Several complement and coagulation factors were detected only in *Mus* and many serine proteases (serpins), proteins associated with inflammation and fibrosis, were present exclusively in *Mus* (Table S5). With regards to macrophage markers, arginase 1 was observed only in *Mus* whereas arginase 2 was solely identified in *Acomys*. Previous studies showed that arginases 1 and 2 as macrophage phenotypic markers are differentially related to inflammatory responses^34^ with arginase 1 being widely expressed in either M1 or M2 polarized environments^35^ and upregulated Arg1 by some M1 macrophages^36^. Yang showed that iNos-independent pro-inflammatory responses mediated by Arg2 in macrophages are due to enhanced mitochondrial ROS, but how Arg2 affects mitochondrial function leading to ROS production in macrophages remains unclear^34^. Notably, an acute inflammatory response was characterized by a higher myeloperoxidase activity in *Mus* and elevated ROS production in *Acomys*^19^, just as we have seen here and ROS production has been suggested as an essential early signal for regeneration based on studies in Xenopus and zebrafish tail models of regeneration^37,38^. Although we have not directly shown that high ROS production is generated specifically by Arg2, its presence identified here in *Acomys* and high ROS production in *Acomys* ear regeneration^19^ may be no coincidence.

On the other hand, macrophage mannose receptor isomers, MRC1 (CD 206) and MRC2 (CD280) were present in both species. Several studies have demonstrated the critical role of the innate immune system in regulating regeneration and in the absence of macrophages *Acomys* ear regeneration and salamander heart regeneration is inhibited^19,39^. Presumably in these classes of macrophages are those that are crucial for non-fibrotic regenerative events across the vertebrates.

The profiles of extracellular matrix (ECM) proteins were different between *Acomys* and *Mus*. 11 members of the alpha collagen family were detected and, after wounding there is an increase in levels in all but one case with generally higher levels in *Mus*, the highest being for COLXII and COLXIV. Gawriluk et al. by transcriptomic studies of ear hole closure showed increased expression of collagen subunits in *Mus* relative to *Acomys*^14^. But in addition to matrix molecules there were several proteases and collagen remodeling proteins which were more highly or exclusively expressed in *Acomys* suggesting that despite the presence of relatively high levels of collagens a less fibrotic matrix could be generated which is certainly seen in histological analyses^18^. For example, CTHRC1 was more highly expressed in *Acomys* which can reduce collagen I mRNA and protein expression, inhibit TGF-β and promote cell migration^40^. The mast cells-related proteases cathepsin G, mast cell protease 4 and tryptase β2 were exclusively detected in *Acomys* and on the other hand the MMP inhibitors TIMP1 and cystatin were solely detected in *Mus*. Thus, a more fibrotic and rigid matrix is generated in *Mus* which may be why we saw integrin binding appearing as an interacting group in the network analysis of *Mus* proteins (Fig. 9B).

It is clear that many differences in protein expression between regenerative repair in *Acomys* and fibrotic repair in *Mus* can be identified. It will be important to design studies to determine if there are particular genetic or metabolic components that can trigger regeneration repair in favor the fibrotic/inflammatory pathways that are normally seen after wounding in mammals.

## Materials and Methods

The following reagents were used: acetonitrile, water, formic acid (all LC-MS grade), TCEP (Tris (2-carboxyethyl) phosphine) and iodoacetamide were purchased from Sigma-Aldrich (St. Louis, MO). Sequence grade of modified trypsin (Pierce trypsin protease, MS grade # 90057) was obtained from Fisher Scientific (Fairlawn, NJ).

### Animals

All experiments were performed following guidelines of the Guide for the Care and Use of Laboratory Animals of the National Institutes of Health. The protocols were approved by the Institutional Animal Care and Use Committee (IACUC) at the University of Florida (# 201203505 (*Mus*) and 201207707 (*Acomys*)) and animals were housed under the care of the University of Florida’s Animal Care Services. *A. cahirinus* were obtained from a breeding colony house at University of Florida and *M. musculus* of the CD-1 outbred strain was purchased from Charles River (Wilmington, MA). Animals were 6 months of age at time of experiments. Animals were anaesthetized with iso-fluorane, the hair on the dorsum was shaved and two 8 mm biopsy punch wounds made through the mid-dorsal full thickness skin. At various times after wounding (3, 5, 7, 14 days) the animals were sacrificed, and the wound tissue dissected out excluding the surrounding normal skin.

### Protein sample preparation

The tissue (100 mg) was homogenized on ice for 30 seconds using a rotor stator type tissue homogenizer (ProScientific Bio-Gen PRO200 Homogenizer; Multi-Gen 7XL Generator Probes) in a protein extraction buffer (1 mL) containing Tris-Cl (50 mM, pH 7.4), NaCl (100 mM), ethylenediaminetetraacetic acid (EDTA, 1 mM), protease inhibitors (1:25 cOmplete UTLRA, Roche), phosphatase inhibitors (1:10 PhosSTOP, Roche) and kept at 4 °C for 1 h. Soluble proteins were separated from undissolved tissue by centrifugation at 80000 g for 30 min at 4 °C. Protein levels were measured using a BCA kit (Pierce) and protein amounts normalized (2 mg/mL) by dilution in protein extraction buffer. For each experiment and each experimental time point, a group size of 3 animals was used.

### Protein digestion and peptide fractions

Protein samples (100 µg) were loaded on a gel (Novex, 8%, Bis-tris), All steps were carried out as described previously^41^. Briefly, electrophoretic migration was performed to fractionate the protein into 10 gel bands. Incised gel bands were reduced by adding 500 µl of TCEP (10 mM) in NH_4_HCO_3_ (100 mM) at 37 °C for 30 min, and then treated with 500 µl of IAA (55 mM) in NH_4_HCO_3_ (100 mM) at RT for 1 h in the dark. After removing the excess regents in-gel tryptic digestion was performed with trypsin (total protein: trypsin  (50:1, in NH_4_HCO_3_ (50 mM) at 37 °C overnight). The following day, trypsinization was quenched by formic acid (10 µL). Digested samples were dried using a Speed Vac and were stored at -80° until used.

### LC-MS/MS analysis

The tryptic digests were analyzed using an LTQ Velos Orbitrap mass spectrometer (Elite Version, Thermo Scientific, San Jose, USA) coupled with an EASY-nLC system (Thermo Scientific, USA) by a nano electrospray ion source. Samples were dissolved in 20 µl of buffer A (0.1% formic acid aq) and 5 µL (1 µg) were injected for each analysis. Data quality and instrument performance were assessed by examining the performance of HeLa protein digest standard (100 ng, cat # 88328, Thermo Scientific) throughout the sequence. Peptides were delivered to a trap column (Acclaim PepMap 100, 75 µm x 2 cm, nano Viper C_18_, 3 µm, Thermo Scientific) at a flow rate of 5 µL/ min in 100% buffer A. After 20 min of loading and washing, peptides were transferred to an analytical column (C_18_ AQ, 3 µm, 100 µm x 25 cm, Nano LC, USA) and separated using a 120 min gradient from 0-40% of solvent B (0.1% formic acid in acetonitrile) at a flow rate of 300 nL/min. The LTQ Velos Orbitrap mass spectrometer was operated in a data-dependent mode switching between MS and MS2. The MS acquisition parameters were as follows: resolution of full scans was 120000 at m/z 400; six data-dependent MS/MS scans were acquired by collision induced dissociation (CID) per one full scan; CID scans were acquired in linear trap quadrupole (LTQ) with 10 ms activation time and 35% normalized collision energy (NCE) in CID; and a 2.0 Da isolation window. Previously fragmented ions were excluded for 60 s for all MS/MS scans. The MS1 mass scan range was 400−2000 *m/z*. The electrospray voltage was 2.2 kV and the capillary temperature was set at 250 °C.

### Database search and data validation

MS/MS spectra were extracted by the MM File Conversion Tool (Version3.9, http://www.massmatrix.net/mm-cgi/downloads.py) and sent to a database search using SEQUEST^42^. They were searched against the integrated proteomics pipeline (IP2): SEQUEST with modified parameters (precursor ion tolerance = 50 ppm, fragment ion tolerance 0 0.8 Da, missed cleavage ag2, modification , carbamidomethyl cysteine (fixed), methionine oxidation (variable), and enzyme (trypsin)). For peptide validation, a 1% false discovery rate (FDR) at the peptide spectral match (PSM) and/ or peptide level was used. We used the ProteinInferencer^43^ (Scripps Research Institute, La Jolla, CA; http://proteomicwiki.com/wiki/index.php/ProteinInferencer) for an integration of all data generated from the three search engines using an FDR  <1.0% at protein level. The detailed calculation method and the search conditions using this program have been reported^41^. Data were searched against a target-decoy Swiss-Prot database, version 2016_08 from mouse (http://www.uniprot.org). We applied a label-free quantitation of the identified peptides to a protein with manual validation. Protein quantification based on the extracted ion chromatogram (XIC) was obtained by extracting the intensity corresponding to the *m/z* of the selected peptides along the LC-MS run and by integrating the peak area at their respective retention time (RT). Normalized protein quantitative values were calculated as log2 (protein area/total protein area) x 10^6^. Reverse decoy matches were removed from the protein identification list. At least 2 unique peptides per protein were required for protein identification. Only proteins that were identified and quantifiable in at least two technical of at least three biological replicates in each group were used for relative quantification. The criteria for identifying differentially expressed proteins (DEPs) were at least a 2 fold change in levels in either direction with a p-value for significance of ≤ 0.01 and coefficients of variation (CV) <20%. Experiments were repeated at least three times and the results analyzed using the unpaired t-test assuming equal variance on the normalized, scaled dataset. and adjusted for the false discovery rate ($\le$ 0.01) using the Benjamini-Hochberg method (BH)^44^.

### GO enrichment/pathway analysis

Classification and functional enrichment analysis of the DEPs were performed using DAVID (Database for Annotation, Visualization and Integrated Discovery, version 6.8), a Bioinformatics Database for the biological process (BP), cellular components (CC) and molecular function (MF). WEB-based Gene Set Analysis Toolkit (WebGestalt, http;//bioinfo.vanderbilt.edu/webgestalt) was used to map the DEPs to KEGG pathway for biological interpretation^45^. Principal components analysis (PCA) was performed using MetaboAnalyst 4.0^46^. Protein-protein interaction were examined using STRING (v10.5)^47^.

### Western blot analysis

Western blotting was performed on the original samples used for MS analysis or on new wounds (n = 3). The lysates from skin were electrophoresed, transferred to nitrocellulose, and probed for selected proteins as described earlier^48^. The primary antibodies used in this study were TIMP1 (1: 200, cat# AF980, R&D), MMP 9 (1: 1000, cat# LS-B2486, LSBio), 14-3-3 (1:500, cat# ab155037, Abcam), actin (1:1000, cat# mAbcam 8226, Abcam), GAPDH (1:5000, cat# mAbcam 9484, Abcam), collagen 1(1:1000, cat# 34710, Abcam), collagen III (1:500, cat# sc-8781, Santa Cruz), S100A8 (1:100, cat# ab178577, Abcam), collagen XII (rabbit polyclonal, kind.pngt of Dr. D. Birk, University of South Florida and Dr M. Mark, University of Cologne, Germany). The pixel density representing each protein was determined by subtracting a background pixel density using Alphaview version 2 from FluorChem E (Proteinsimple CA, USA).

## Supplementary information

Supplementary Dataset 1.

## Data Availability

MS data have been deposited in the repository with the dataset identified MassIVE MSV000084615.

## References

[1] Clark, R. A. F. & Henson, P. M. *The molecular and cellular biology of wound repair*. xxii, 597 (Plenum, 1988).

[2] Seifert AW, Maden M (2014). New insights into vertebrate skin regeneration. Rev. Cell Int. Mol. Biol..

[3] Seifert AW, Monaghan JR, Voss SR, Maden M (2012). Skin regeneration in adult axolotls: a blueprint for scar-free healing in vertebrates. PLoS One.

[4] Levesque M, Villard E, Roy S (2010). Skin wound healing in axolotls: a scarless process. J. Exp. Zool. Part. B, Mol. Dev. Evol..

[5] Larson BJ, Longaker MT, Lorenz HP (2010). Scarless fetal wound healing: a basic science review. Plast. Reconstr. Surg..

[6] Lo DD, Zimmermann AS, Nauta A, Longaker MT, Lorenz HP (2012). Scarless fetal skin wound healing update. Birth Defects Res. Part C. Embryo Today: Rev..

[7] Satish, L., & Kathju, S. Cellular and molecular characteristics of scarless versus fibrotic wound healing. *Derm. Res. Practice*. 790234 (2010). 10.1155/2010/790234 PMC3021858 21253544

[8] Olutoye OO, Barone EJ, Yager DR, Cohen IK, Diegelmann RF (1997). Collagen induces cytokine release by fetal platelets: implications in scarless healing. J. Pedia. Surg..

[9] Sullivan KM, Lorenz HP, Meuli M, Lin RY, Adzick NS (1995). A model of scarless human fetal wound repair is deficient in transforming growth factor beta. J. Pedia. Surg..

[10] Vorontsova, M. A. & Liosner, L. D. *Asexual Propagation and Regeneration* (Pergamon 1960).

[11] Seifert AW (2012). Skin shedding and tissue regeneration in African spiny mice (Acomys). Nature.

[12] Clark LD, Clark RK, Heber-Katz E (1998). A new murine model for mammalian wound repair and regeneration. Clin. Immunol. Immunopathol..

[13] Nelson AM (2013). Prostaglandin D2 Inhibits Wound-Induced Hair Follicle Neogenesis through the Receptor, Gpr44. J. Invest. Dermatol..

[14] Gawriluk TR (2016). Comparative analysis of ear-hole closure identifies epimorphic regeneration as a discrete trait in mammals. Nat. Commun..

[15] Santos DM (2016). Ear wound regeneration in the african spiny mouse Acomys cahirinus. Regeneration..

[16] Maden. M (2018). Optimal skin regeneration after full thickness thermal burn injury in the spiny mouse, Acomys cahirinus. Burns..

[17] Brant JO, Lopez M-C, Baker HV, Barbazuk WB, Maden M (2015). A., Comparative analysis of gene expression profiles during skin regeneration in Mus and Acomys. PLoS One..

[18] Brant JO, Yoon JH, Polvadore T, Barbazuk WB, Maden M (2016). Cellular events during scar-free skin regeneration in the spiny mouse, Acomys. Wound. Repair. Regen..

[19] Simkin J, Gawriluk TR, Gensel JC, Seifert AW (2017). Macrophages are necessary for epimorphic regeneration in African spiny mice. eLife..

[20] Brant JO (2019). Comparative transcriptomic analysis of dermal wound healing reveals de nove skeletal muscle regeneration in Acomys cahirinus. PLoS One..

[21] Bachofner M (2017). Large-scale quantitative proteomics identifies the ubiquitin ligase Nedd4-1 as an essential regulator of liver regeneration. Dev. Cell..

[22] Hsu YC, Li L, Fuchs E (2014). Emerging interactions between skin stem cells and their niches. Nat. Med..

[23] Fuchs E (2007). Scratching the surface of skin development. Nature.

[24] Myung PS, Takeo M, Ito M, Atit RP (2013). Epithelial Wnt ligand secretion is required for adult hair follicle growth and regeneration. J. Invest. Dermatol..

[25] Okuse T, Chiba T, Katsumi I, Imai K (2005). Differential expression and localization of WNTs in an animal model of skin wound healing. Wound. Repair. Regen..

[26] Ito M (2007). Wnt-dependent de novo hair follicle regeneration in adult mouse skin after wounding. Nature.

[27] Stewart DC (2018). Unique behavior of dermal cells from regenerative mammal, the African spiny Mouse, in response to substrate stiffness. J. Biomech..

[28] Kim S, Wong P, Coulombe PA (2006). Keratin cytoskeletal protein regulates protein synthesis and epithelial cell growth. Nature.

[29] Staley JP, Woolford JJ (2009). Assembly of ribosomes and spliceosomes: complex ribonucleoprotein machines. Curr. Opin. Cell Biol..

[30] Gu LH, Coulombe PA (2006). Keratin function in skin epithelia: a broadening palette with surprising shades. Curr. Opin. Cell Biol..

[31] Gawronska-Kozak, B. & Bukowska, J. *Animal models of skin regeneration in animal models for the study of human disease* (2nd edition), 343–356 (2017).

[32] Maden M (2018). Perfect chronic skeletal muscle regeneration in adult spiny mice, Acomys cahirinus. Sci. Rep..

[33] Okamura, D. M. *et al*. Scarless repair of acute and chronic kidney injury in African Spiny mice (*Acomys cahirinus*). *bioRxiv* 315069 (2018).

[34] Yang, Z. & Ming, X. F. Functions of arginase isoforms in macrophage inflammatory responses: impact on cardiovascular diseases and metabolic disorders. *Front. Immunol*., 10.3389/fimmu.2014.00533 (2014). 10.3389/fimmu.2014.00533 PMC4209887 25386179

[35] Murray P, Wynn TA (2011). Protective and pathogenic functions of macrophage subsets. Nat. Immunol..

[36] Jablonski KA (2015). Novel markers to delineate Murine M1 and M2 macrophages. PLoS One..

[37] Gauron C (2013). Sustained production of ROS triggers compensatory proliferation and is required for regeneration to proceed. Sci. Rep..

[38] Love NR (2013). Amputation-induced reactive oxygen species are required for successful xenopus tadpole tail regeneration. Nat. Cell. Biol..

[39] Godwin JW, Debuque R, Salimova E, Rosenthal NA (2017). Heart regeneration in the Salamander relies on macrophage-mediated control of fibroblast activation and the extracellular landscape. NPJ Reg. Med..

[40] LeClair RJ (2007). Cthrc1 is a novel inhibitor of transforming growth factor-beta signaling and neointimal lesion formation. Circ. Res..

[41] Cho K (2012). Quantitative phosphoproteomics of the human neural stem cell differentiation into oligodendrocyte by mass spectrometry. Mass. Spectrom. Lett..

[42] Eng JK, McCormack AL, Yates JR (1994). An approach to correlate tandem mass spectral data of peptides with amino acid sequences in a protein database. J. Am. Soc. Mass. Spectrom..

[43] Zhang Y (2015). ProteinInferencer: Confident protein identification and multiple experiment comparison for large scale proteomics projects. J. Proteom..

[44] Benjamini Y, Hochberg Y (1995). Controlling the False Discovery Rate: a practical and powerful approach to multiple testing. J. R. Statist. Soc. B.

[45] Zhang B, Kirov S, Snoddy J (2005). WebGestalt: an integrated system for exploring gene sets in various biological contexts. Nuceicl. Acids. Res..

[46] Chong J (2018). MetaboAnalyst 4.0: towards more transparent and integrative metabolomics analysis. Nucleic. Acids. Res..

[47] Szklarczyk D (2015). STRINGv10: Protein–protein interaction networks, integrated over the tree of life. Nucleic Acids Res..

[48] Yoon JH (2012). Comparative proteomic profiling of dystroglycan-associated proteins in wild type, mdx, and galgt2 transgenic mouse skeletal muscle. J. Proteome. Res..

