# Author Correction: Comparative Proteomic Analysis in Scar-Free Skin Regeneration in *Acomys cahirinus* and Scarring *Mus musculus*

**DOI:** 10.1038/s41598-020-65513-z

**Published:** 2020-05-20

**Authors:** Jung Hae Yoon, Kun Cho, Timothy J. Garrett, Paul Finch, Malcolm Maden

**Affiliations:** 1 Department of Biology & UF Genetics Institute, 2033 Mowry Road, University of Florida, Gainesville, Florida 32610 USA; 2 Biomedical Omics Group, Korea Basic Science Institute, Ochang, 863-883 Republic of Korea; 3 Department of Pathology, Immunology, and Laboratory Medicine, University of Florida, Gainesville, Florida USA; 4 School of Biological Sciences, Royal Holloway, University of London, Egham, Surrey UK

Correction to: *Scientific Reports* 10.1038/s41598-019-56823-y, published online 13 January 2020

This Article contains errors in Table 1 where a block of numbers in the ubiquitin/proteasome section is inadvertently duplicated. The correct Table 1 appears below.

**Table 1:** Proteins identified from *Acomys* and *Mus* associated with wound healing over 14 days.

Accession	Protein Description	Gene	***Acomys***					***Mus***				
			**0 day**	**3 days**	**5 days**	**7 days**	**14days**	**0 day**	**3 days**	**5 days**	**7 days**	**14days**
			Quantitative Value (CV)									
**Ubiquitin/Proteasome**												
O88685	26S protease regulatory subunit 6A	Psmc3	1.55(20.4)	4.43(9.6)	4.19(5.9)	3.39(1.3)	4.32(6.1)	3.26(16.6)	3.56(18.2)	2.69(7.8)	1.45(9.1)	5.27(3.0
Q6ZPJ3	E2/E3 hybrid ubiquitin-protein ligase UBE2O	Ube2o	1.04(19.2)	4.45(5.5)	4.02(7.8)	4.00(10.3)	3.14(13.6)					
Q3U319	E3 ubiquitin-protein ligase BRE1B	Rnf40	1.55(17.9)	2.23(22.4)	2.04(19.6)	3.02(13.9)	2.51(19.6)					
P46935	E3 ubiquitin-protein ligase NEDD4	Nedd4	3.34(21.8)	2.84(21.3)	4.82(19.6)	6.05(11.1)	6.51(14.5)	4.64(17.4)	1.04(12.4)	3.33(5.0)	2.92(20.6)	5.73(7.0)
P0CG49	Polyubiquitin-B	Ubb	3.16(5.1)	4.66(17.7)	5.28(5.0)	5.92(1.6)	5.14(19.4)	5.07(2.9)	5.42(7.3)	5.05(10.3)	5.08(3.4)	5.58(6.3)
P0CG50	Polyubiquitin-C	Ubc		5.26(3.7)	5.32(8.2)	5.93(1.7)	4.95(21.3)	4.39(10.4)	5.77(17.3)	4.84(10.8)	5.08(4.9)	5.56(9.0)
Q9Z2U1	Proteasome subunit alpha type-5	Psma5	2.05(19.1)	3.93(16.3)	4.47(3.4)	3.75(10.7)	4.10(19.0)	2.98(16.1)	4.00(8.5)	3.38(11.1)	2.46(6.8)	2.81(16.3)
Q6ZQ93	Ubiquitin carboxyl-terminal hydrolase 34	Usp34	0.94(16.4)	1.86(16.1)	1.69(17.7)	1.64(18.3)	1.62(12.3)					
Q6A4J8	Ubiquitin carboxyl-terminal hydrolase 7	Usp7	1.04(19.2)	1.42(21.1)	1.74(17.3)	1.83(16.4)	2.00(16.1)					
P62984	Ubiquitin-60S ribosomal protein L40	Uba52	2.23(8.3)	5.26(3.7)	5.32(5.8)	5.93(1.7)	5.28(18.9)	5.02(2.4)	5.38(10.3)	5.34(2.5)	4.84(1.9)	5.42(5.5)
P52482	Ubiquitin-conjugating enzyme E2 E1	Ube2e1	1.84(14.6)	3.34(15.6)	1.87(13.3)	1.79(2.5)	3.82(13.1)					
Q8K2Z8	Ubiquitin-conjugating enzyme E2 Q2	Ube2q2	2.45(19.5)	2.50(16.0)	2.51(17.7)	1.44(14.5)	3.51(4.3)					
Q02053	Ubiquitin-like modifier-activating enzyme 1	Uba1	4.53(14.8)	4.99(21.4)	5.41(2.4)	5.45(7.2)	5.34(18.0)	5.93(6.4)	5.56(13.1)	5.37(19.6)	5.57(15.4)	7.28(2.3)
**Ribosome**												
P62301	40S ribosomal protein S13	Rps13	1.55(17.9)	3.05(6.1)	4.13(13.6)	4.49(8.0)	3.92(8.0)	3.35(15.7)	3.04(4.2)	3.62(6.8)	3.05(5.5)	2.73(15.9)
P63276	40S ribosomal protein S17	Rps17	1.55(17.9)	1.86(16.1)	3.01(10.5)	4.20(10.4)	2.89(3.4)	3.32(15.9)	2.04(8.9)	1.61(18.6)	1.35(14.8)	1.73(13.5)
P62855	40S ribosomal protein S26	Rps26	0.94(16.4)	2.05(9.1)	3.82(20.1)	3.47(20.6)	3.89(2.6)	3.77(18.8)	2.63(4.9)	3.13(9.1)	2.76(8.9)	3.17(13.1)
P19253	60S ribosomal protein L13a	Rpl13a	1.05(21.3)	2.37(16.5)	4.90(17.2)	3.94(15.0)	3.47(13.0)	3.23(13.3)	3.08(4.2)	2.59(4.5)	2.93(17.1)	3.86(11.0)
P14115	60S ribosomal protein L27a	Rpl27a	1.55(17.9)	4.58(9.0)	4.65(18.4)	3.81(13.1)	4.21(13.0)	4.07(21.1)	3.61(4.5)	3.30(7.5)	2.33(13.5)	5.50(19.2)
Q9D8E6	60S ribosomal protein L4	Rpl4	3.00(19.7)	5.12(5.9)	5.01(19.6)	5.48(5.7)	7.04(1.1)	5.47(21.3)	4.89(12.2)	4.67(6.7)	4.53(19.5)	6.66(9.5)
P62983	Ubiquitin-40S ribosomal protein S27a	Rps27a	2.23(11.7)	4.66(17.7)	5.39(6.2)	5.93(1.7)	5.02(15.0)	4.70(12.8)	5.42(7.3)	4.97(14.3)	4.98(5.0)	5.58(6.3)
**Spliceosome**												
O08810	116 kDa U5 small nuclear ribonucleoprotein component	Eftud2	1.73(17.4)	2.05(9.1)	4.39(7.9)	4.36(12.3)	5.21(17.9)	3.78(13.0)	4.17(17.7)	3.80(2.4)	3.79(16.2)	5.66(8.6)
P17879	Heat shock 70 kDa protein 1B	Hspa1b	5.50(3.5)	4.83(20.6)	5.74(8.7)	5.88(2.0)	2.10(13.2)	4.77(16.5)	5.78(3.3)	5.40(3.1)	5.82(5.2)	7.16(0.2)
P49312	Heterogeneous nuclear ribonucleoprotein A1	Hnrnpa1	0.94(16.4)	2.43(15.4)	4.53(3.6)	5.03(11.9)	5.44(8.4)	4.22(13.9)	3.69(16.9)	3.22(15.5)	2.74(8.2)	5.24(17.2)
Q62093	Serine/arginine-rich splicing factor 2	Srsf2	1.66(17.2)	1.64(13.4)	2.82(6.8)	3.39(1.3)	4.00(9.5)	2.41(17.9)	1.79(21.5)	2.59(6.4)	2.87(14.1)	3.32(13.1)
P62317	Small nuclear ribonucleoprotein Sm D2	Snrpd2	1.27(15.7)	1.86(16.1)	3.66(14.8)	4.42(12.9)	2.89(3.4)	2.30(14.1)	2.54(20.6)	1.12(17.8)	1.62(18.5)	2.23(4.2)
Q9Z1N5	Spliceosome RNA helicase Ddx39b	Ddx39b	2.55(20.5)	4.36(16.9)	5.23(11.8)	5.54(6.6)	5.58(10.9)	4.40(5.0)	4.48(2.2)	4.36(7.6)	4.11(19.9)	5.67(1.8)
Q921M3	Splicing factor 3B subunit 3	Sf3b3	1.94(7.9)	2.05(9.1)	5.43(9.6)	5.39(2.8)	5.33(16.6)	4.01(19.9)	4.42(11.3)	4.03(15.4)	3.86(18.5)	5.33(3.7)
**Protein Processing in Endoplasmic Reticulum**												
P14211	Calreticulin	Calr	3.56(11.5)	2.55(15.2)	4.99(12.2)	5.29(18.8)	5.48(10.5)	4.57(11.8)	4.83(3.1)	5.21(12.4)	5.34(12.3)	5.81(9.7)
P35564	Calnexin	Canx	3.13(13.5)	4.73(18.0)	4.39(21.0)	4.79(11.6)	5.54(1.4)	5.16(8.8)	5.14(17.9)	5.14(6.2)	4.77(12.1)	6.06(9.0)
O54734	Dolichyl-diphospho oligosaccharide--protein glycosyl transferase 48 kDa subunit	Ddost	1.05(11.7)	2.05(12.9)	4.32(1.0)	3.84(14.5)	4.63(11.6)	3.95(12.4)	3.92(14.6)	4.08(13.1)	3.21(12.2)	5.27(4.7)
Q922R8	Protein disulfide-isomerase A6	Pdia6	2.55(15.4)	4.72(14.3)	5.36(6.5)	5.89(2.0)	5.85(3.4)	5.05(8.5)	5.29(9.3)	5.45(1.4)	4.96(15.4)	6.32(9.9)
Q91W90	Thioredoxin domain-containing protein 5	Txndc5	1.94(7.9)	3.74(13.4)	3.18(14.0)	3.10(10.9)	4.14(10.3)	2.76(21.7)	1.04(17.5)	3.41(15.3)	3.12(16.2)	5.45(4.1)
**Keratin**												
Q61765	Keratin, type I cuticular Ha1	Krt31	8.16(14.5)	7.11(15.9)	6.55(15.6)	7.48(14.4)	7.74(11.6)	6.16(12.8)	6.48(17.0)	6.23(9.5)	6.32(3.0)	7.77(8.5)
Q62168	Keratin, type I cuticular Ha2	Krt32	7.74(19.6)	7.04(16.0)	6.09(21.1)	7.13(9.1)	7.75(11.8)	5.84(12.2)	5.96(10.6)	6.08(6.3)	5.99(11.2)	6.57(2.0)
Q61897	Keratin, type I cuticular Ha3-II	Krt33b	7.93(13.7)	7.01(14.5)	6.55(15.6)	7.41(14.7)	7.73(11.5)	6.12(12.3)	6.46(16.4)	6.25(9.9)	6.30(4.9)	7.68(7.3)
Q497I4	Keratin, type I cuticular Ha5	Krt35	7.89(15.4)	7.00(14.2)	6.17(18.5)	7.31(12.2)	7.76(11.8)	6.00(11.0)	6.28(13.0)	6.25(8.0)	6.26(6.3)	7.15(4.8)
B1AQ75	Keratin, type I cuticular Ha6	Krt36	7.78(17.6)	6.86(14.4)	5.84(21.9)	7.13(9.6)	7.61(10.5)	5.80(12.9)	5.96(10.6)	6.07(6.1)	5.99(11.2)	6.64(1.3)
Q9QWL7	Keratin, type I cytoskeletal 17	Krt17	9.12(5.0)	9.20(12.2)	8.78(7.5)	9.50(1.9)	9.36(4.7)	8.93(3.7)	8.16(6.1)	8.57(1.8)	8.46(2.8)	9.04(1.4)
**Protein Phosphorylation**												
P11440	Cyclin-dependent kinase 1	Cdk1	1.27(15.7)	1.42(14.1)	2.37(11.6)	3.42(0.5)	1.94(20.2)					
P31938	Dual specificity mitogen-activated protein kinase kinase 1	Map2k1	1.95(14.2)	3.74(13.4)	4.23(4.5)	3.34(16.1)	3.26(19.0)					
Q01279	Epidermal growth factor receptor	Egfr		1.42(14.1)	1.89(11.5)	2.44(12.3)	1.60(19.6)	3.10(10.1)	5.82(4.3)	4.11(22.0)	4.57(19.5)	4.67(5.4)
P42567	Epidermal growth factor receptor substrate 15	Eps15		1.86(16.1)	1.74(11.5)	2.44(12.3)	1.50(11.5)	2.07(18.7)	1.40(14.3)			2.79(10.3)
P16092	Fibroblast growth factor receptor 1	Fgfr1	1.27(15.7)	1.42(14.1)	1.87(13.3)	2.92(4.7)	1.72(8.2)					
P18653	Ribosomal protein S6 kinase alpha-1	Rps6ka1	1.05(21.3)	1.42(14.1)	1.74(11.5)	1.83(10.9)	1.38(14.5)					
O55098	Serine/threonine-protein kinase 10	Stk10	1.16(14.0)	3.01(16.6)	1.74(11.5)	1.76(11.4)	1.62(12.3)					
P83741	Serine/threonine-protein kinase WNK1	Wnk1	1.04(9.6)	1.42(14.1)	1.89(11.5)	1.44(13.9)	1.60(19.6)		1.17(8.5)		2.35(12.8)	3.60(5.2)
P16277	Tyrosine-protein kinase Blk	Blk	1.27(15.7)	1.42(14.1)	1.87(13.3)	2.63(10.5)	1.72(8.2)					
P41241	Tyrosine-protein kinase CSK	Csk	2.63(15.2)	3.70(12.5)	3.02(17.5)	3.10(15.4)	3.14(13.6)					
P14234	Tyrosine-protein kinase Fgr	Fgr	1.27(15.7)	2.42(16.5)	1.87(13.3)	2.63(10.5)	1.72(8.2)					
Q922K9	Tyrosine-protein kinase FRK	Frk	2.63(15.2)	3.63(7.3)	3.18(19.8)	3.10(15.4)	2.80(14.8)					
**Protease**												
P10605	Cathepsin B	Ctsb	1.05(21.3)	2.83(21.0)	3.60(22.8)	2.79(12.7)	3.00(20.7)	5.16(16.1)	4.24(17.5)	5.24(14.7)	4.86(2.9)	5.15(15.0)
P18242	Cathepsin D	Ctsd	1.05(21.3)	2.14(13.1)	2.37(19.4)	3.59(4.8)	2.94(21.4)	4.01(17.3)	4.24(16.3)	3.63(18.1)	4.62(8.7)	4.79(1.1)
P28293	Cathepsin G	Ctsg		4.44(15.9)	3.02(17.5)	1.60(14.4)	2.10(18.7)					
P49935	Pro-cathepsin H	Ctsh						2.90(22.1)	2.04(8.9)	1.68(17.7)	1.35(14.8)	2.78(21.9)
P06797	Cathepsin L1	Ctsl						1.53(16.7)	1.17(8.5)	1.18(10.8)	1.46(11.4)	1.17(8.5)
Q9WUU7	Cathepsin Z	Ctsz	1.04(19.2)	2.37(16.5)	3.55(6.8)	3.64(5.5)	3.45(10.1)	3.15(12.1)	3.20(1.4)	3.80(12.1)	3.79(8.3)	3.15(12.3)
P26262	Plasma kallikrein	Klkb1						2.56(16.3)	6.19(3.3)	6.10(1.6)	6.96(2.4)	3.86(17.4)
P21812	Mast cell protease 4	Mcpt4	3.04(16.4)	2.42(12.4)	3.32(15.1)	3.34(15.0)	4.43(11.3)					
P41245	Matrix metallo proteinase-9	Mmp9							6.81(5.2)	6.26(12.2)	5.96(12.8)	2.97(16.8)
P21845	Tryptase beta-2	Tpsb2	1.04(9.6)	1.42(14.1)	1.42(14.1)	1.74(17.3)	1.62(18.5)					
**Protease Inhibitors**												
Q61247	Alpha-2-antiplasmin	Serpinf2		3.70(21.3)	3.66(1.2)	4.17(18.8)	2.27(9.7)	2.17(13.8)	6.12(1.4)	5.58(11.6)	6.70(2.5)	1.78(22.0)
Q61838	Alpha-2-macroglobulin	Pzp	2.63(19.0)	7.36(9.8)	6.91(3.3)	7.59(9.7)	6.27(8.2)	6.93(3.8)	10.39(3.6)	10.41(0.5)	10.75(2.5)	7.81(2.9)
Q6GQT1	Alpha-2-macroglobulin-P	A2mp	1.04(19.2)	7.32(12.8)	6.50(2.7)	7.79(12.6)	5.95(8.5)	2.71(12.4)	5.87(6.6)	5.99(1.6)	6.32(2.9)	3.83(15.3)
P32261	Antithrombin-III	Serpinc1	1.04(19.2)	6.00(12.1)	5.18(15.9)	5.69(14.1)	3.91(13.2)	2.38(19.6)	7.03(4.6)	6.21(6.2)	7.35(3.6)	4.17(16.3)
Q62426	Cystatin-B	Cstb						1.10(19.9)	0.91(10.9)	1.47(13.6)	1.58(12.7)	1.73(19.3)
P49182	Heparin cofactor 2	Serpind1	1.04(19.2)	2.05(12.9)	3.05(14.6)	3.29(22.8)	2.96(16.9)	2.27(13.1)	5.83(8.0)	5.42(8.2)	6.15(4.1)	3.44(2.1)
O08677	Kininogen-1	Kng1						2.65(18.7)	7.63(0.6)	6.83(6.3)	7.90(6.2)	3.70(14.3)
P12032	Metalloproteinase inhibitor 1	Timp1						0.58(8.6)	1.17(17.1)	2.71(14.8)	2.12(19.2)	
P97290	Plasma protease C1 inhibitor	Serping1	1.66(9.8)	6.04(9.8)	4.79(7.4)	5.51(4.7)	5.56(10.3)	3.27(20.7)	6.95(4.7)	5.96(5.6)	6.89(4.2)	4.34(20.7)
**ECM(Extra Cellular Matrix)**												
P11087	Collagen alpha-1(I) chain	Col1a1	2.71(22.8)	2.05(12.9)	5.08(20.0)	4.40(13.6)	5.02(17.5)	3.56(15.6)	4.11(11.1)	3.86(3.8)	3.90(6.6)	7.05(4.9)
P08121	Collagen alpha-1(III) chain	Col3a1	1.16(9.9)	1.42(14.1)	3.18(14.0)	4.63(5.9)	4.64(20.9)	2.65(18.4)	2.50(13.6)	2.80(3.2)	4.45(10.8)	5.63(13.8)
Q04857	Collagen alpha-1(VI) chain	Col6a1	2.34(31.1)	4.74(21.1)	6.26(12.8)	4.46(14.2)	4.68(8.4)	4.03(21.8)	2.82(20.9)	3.51(18.9)	3.14(6.2)	7.32(17.7)
Q60847	Collagen alpha-1(XII) chain	Col12a1	3.00(4.8)	2.33(4.0)	5.70(17.6)	5.41(0.0)	7.41(0.1)	2.54(20.4)	4.15(10.3)	7.13(20.8)	5.98(10.1)	9.29(10.2)
Q80X19	Collagen alpha-1(XIV) chain	Col14a1	4.04(10.0)	5.32(16.3)	7.21(11.4)	7.68(2.9)	8.23(9.5)	6.63(19.4)	6.94(19.1)	5.43(11.7)	7.01(2.8)	9.08(6.5)
O35206	Collagen alpha-1(XV) chain	Col15a1	1.55(20.4)	1.42(14.1)	2.68(16.4)	1.60(10.2)	1.72(5.8)	3.63(11.1)	1.04(17.5)	3.51(10.9)	2.49(7.0)	4.06(19.7)
Q07563	Collagen alpha-1(XVII) chain	Col17a1						0.17(17.6)	1.16(21.1)	0.89(11.2)	1.32(15.2)	1.39(14.4)
P39061	Collagen alpha-1(XVIII) chain	Col18a1	3.04(19.7)	(0.0)	2.74(18.3)	1.60(10.2)	2.62(19.1)	3.92(12.2)	1.40(14.3)	4.00(2.3)	2.33(22.1)	3.87(15.7)
Q01149	Collagen alpha-2(I) chain	Col1a2	4.29(8.0)	2.62(14.8)	4.04(0.5)	5.07(14.9)	6.36(8.8)	4.07(16.5)	4.70(4.5)	2.96(16.5)	4.14(16.5)	7.71(8.0)
Q02788	Collagen alpha-2(VI) chain	Col6a2	4.92(21.5)	3.74(13.4)	3.93(17.5)	4.29(19.3)	3.76(2.3)	2.75(20.8)	1.95(17.8)	1.01(11.6)	2.99(22.6)	6.62(21.0)
Q9D1D6	Collagen triple helix repeat-containing protein 1	Cthrc1	0.83(12.1)	1.64(18.3)	2.39(20.5)	3.09(7.4)	4.44(12.8)	2.14(18.7)	1.16(8.6)	0.89(16.9)	1.35(22.2)	3.47(6.1)
Q80YX1	Tenascin	Tnc		3.05(13.6)	6.86(18.4)	8.73(2.0)	8.74(4.1)	2.07(19.9)	5.80(24.3)	7.01(21.7)	7.43(5.4)	8.25(2.7)
**Complement and Coagulation Factors**												
P08607	C4b-binding protein	C4bp							4.63(4.2)	3.57(5.0)	4.49(6.3)	2.39(20.9)
O88947	Coagulation factor X	F10		3.28(13.5)	3.82(14.2)	1.60(14.4)	1.38(21.8)	1.56(19.2)	4.81(19.6)	2.47(19.5)	4.98(15.5)	1.23(7.5)
Q80YC5	Coagulation factor XII	F12							3.24(21.0)	2.30(10.8)	3.67(16.3)	1.28(12.1)
Q8CG14	Complement C1s-A subcomponent	C1s							3.55(16.7)	2.30(10.7)	4.50(11.4)	2.28(55.3)
P21180	Complement C2	C2	1.27(15.7)	2.14(18.6)	1.87(13.3)	2.59(17.7)	1.38(21.8)		5.10(17.9)	2.36(8.6)	4.19(5.9)	1.28(12.1)
P01027	Complement C3	C3	3.99(8.5)	8.52(11.7)	8.20(4.9)	8.61(8.1)	7.36(3.9)	7.57(2.4)	11.07(1.9)	10.64(1.7)	11.05(2.5)	8.22(2.2)
Q8K182	Complement component C8 alpha chain	C8a							6.39(5.5)	4.02(14.0)	3.14(6.2)	3.36(25.6)
Q8VCG4	Complement component C8 gamma chain	C8g						0.58(17.2)	5.10(20.0)	2.30(17.8)	4.34(13.4)	1.39(14.4)
P06683	Complement component C9	C9						0.38(14.8)	6.04(10.3)	4.76(5.5)	4.98(15.8)	3.78(16.2)
P03953	Complement factor D	Cfd						0.36(12.1)	1.69(19.5)	2.69(10.8)	2.86(15.5)	1.29(8.6)
Q61129	Complement factor I	Cfi	1.05(21.3)	4.20(16.7)	2.89(7.5)	4.12(14.9)	2.39(19.7)	0.17(11.8)	5.18(0.5)	4.88(10.6)	5.48(11.0)	1.28(8.6)
E9PV24	Fibrinogen alpha chain	Fga	3.05(7.3)	3.83(15.0)	4.48(3.4)	4.58(22.4)	5.16(12.8)	5.08(17.0)	6.54(0.6)	6.98(2.6)	7.86(5.8)	5.93(19.7)
Q8K0E8	Fibrinogen beta chain	Fgb	3.97(15.9)	7.28(19.4)	7.81(4.2)	8.34(11.7)	7.79(3.0)	5.91(16.2)	7.67(5.9)	8.12(7.8)	8.36(4.6)	6.59(2.5)
Q8VCM7	Fibrinogen gamma chain	Fgg	5.51(18.1)	6.21(19.9)	6.69(5.4)	7.48(11.6)	6.48(7.3)	5.97(17.4)	7.74(5.3)	8.04(9.0)	8.56(4.4)	6.43(5.3)
P20918	Plasminogen	Plg	4.40(18.4)	8.01(5.2)	7.53(11.6)	8.46(11.8)	6.76(15.4)	4.91(11.7)	9.34(2.1)	9.19(1.8)	9.65(2.0)	5.69(19.7)
P19221	Prothrombin	F2	2.73(20.8)	6.40(16.6)	6.08(11.8)	6.61(17.7)	5.26(7.3)	2.81(16.7)	7.39(8.9)	7.07(5.4)	8.15(3.3)	5.52(0.9)
**Immunomodulators**												
P08071	Lactotransferrin	Ltf	1.04(19.2)	7.58(3.5)	6.91(6.1)	7.57(9.7)	6.49(0.4)	3.39(16.9)	9.88(6.2)	9.70(6.4)	9.63(5.0)	5.94(21.5)
P11247	Myeloperoxidase	Mpo	1.04(19.2)	6.70(15.2)	6.48(9.4)	6.42(16.4)	5.04(9.7)	3.17(18.9)	7.62(5.8)	7.52(6.9)	7.69(7.2)	3.13(14.7)
P50543	Protein S100-A11	S100a11		2.33(17.4)	2.37(11.6)	3.26(6.4)	2.82(17.7)	3.73(18.2)	4.53(7.0)	5.78(6.1)	4.28(13.2)	4.39(14.5)
P14069	Protein S100-A6	S100a6						2.10(21.1)	1.54(17.5)	2.67(20.4)	2.87(18.2)	2.73(15.9)
P27005	Protein S100-A8	S100a8							6.08(13.7)	8.81(11.9)	4.57(12.4)	2.17(9.2)
P31725	Protein S100-A9	S100a9							7.76(10.2)	8.15(10.3)	7.48(13.0)	2.03(17.7)
**Macropage Markers**												
O08691	Arginase-2, mitochondrial	Arg2		1.83(22.2)	2.04(14.7)	2.10(17.3)	1.61(13.8)					
Q61176	Arginase-1	Arg1						1.38(21.1)	6.07(11.3)	5.63(9.2)	5.95(7.6)	4.52(16.7)
Q61830	Macrophage mannose receptor 1	Mrc1	1.55(20.4)	3.30(13.4)	5.25(7.5)	4.33(1.9)	4.38(18.7)	5.04(15.8)	5.33(16.9)	5.43(9.2)	5.05(17.2)	6.79(8.8)
Q64449	C-type mannose receptor 2	Mrc2	1.27(15.7)	1.42(14.1)	1.89(8.1)	3.31(15.3)	4.00(22.0)	2.00(18.4)	2.45(2.7)	3.33(3.5)	3.94(9.7)	4.65(16.8)
**Others**												
O70456	14-3-3 protein sigma	Sfn	5.69(5.2)	6.61(8.1)	7.13(2.9)	7.19(3.8)	6.73(4.8)	6.42(14.9)	5.09(15.1)	5.56(4.3)	6.18(2.0)	6.30(10.3)
P63101	14-3-3 protein zeta/delta	Ywhaz	5.88(5.0)	6.69(10.8)	7.29(6.1)	7.36(2.7)	6.80(2.8)	6.81(10.5)	5.82(18.7)	6.59(1.1)	6.72(5.4)	6.62(9.9)
P62737	Actin, aortic smooth muscle	Acta2	10.82(2.8)	10.79(18.5)	11.19(5.2)	10.56(1.2)	10.79(5.3)	10.22(10.5)	10.31(2.6)	9.92(1.4)	10.00(15.0)	9.37(2.3)
Q9WV32	Actin-related protein 2/3 complex subunit 1B	Arpc1b	2.16(7.5)	4.94(19.1)	4.85(9.5)	4.84(7.5)	3.97(14.4)	2.30(16.3)	4.42(1.4)	4.14(15.4)	4.55(8.9)	3.68(18.8)
Q9JM76	Actin-related protein 2/3 complex subunit 3	Arpc3	1.05(21.3)	5.18(9.9)	5.32(0.8)	4.56(7.8)	4.59(12.4)	3.73(14.9)	5.31(7.4)	4.36(14.0)	4.61(20.8)	3.61(18.5)
Q91V92	ATP-citrate synthase	Acly	4.82(19.4)	3.22(16.0)	5.52(12.2)	5.22(15.4)	5.30(19.6)	5.76(15.7)	4.05(13.3)	4.62(3.8)	4.34(22.9)	6.42(8.8)
P26231	Catenin alpha-1	Ctnna1	3.72(20.6)	1.64(13.4)	3.89(21.8)	4.51(17.4)	5.02(17.2)	5.33(11.6)	1.16(21.1)	3.96(12.3)	4.15(14.4)	6.23(5.1)
Q61301	Catenin alpha-2	Ctnna2	2.82(19.4)	5.08(20.6)	3.39(10.2)	3.73(16.4)	3.89(12.5)	3.59(21.5)	1.16(21.1)	1.80(22.7)	2.35(21.3)	4.32(14.6)
Q9CZ13	Cytochrome b-c1 complex subunit 1, mitochondrial	Uqcrc1	2.45(10.3)	3.80(10.0)	1.89(8.1)	2.10(14.3)	2.29(18.1)	4.51(22.4)	3.66(12.5)	4.31(8.9)	3.88(16.1)	4.37(9.4)
Q00612	Glucose-6-phosphate 1-dehydrogenase X	G6pdx	3.30(12.2)	5.91(18.2)	6.00(7.0)	5.46(19.7)	4.86(1.9)	3.51(21.6)	6.42(10.4)	5.75(17.6)	5.54(13.6)	3.78(16.5)
P63017	Heat shock cognate 71 kDa protein	Hspa8	7.55(13.6)	7.99(5.6)	8.01(8.0)	8.41(3.7)	8.41(3.9)	8.02(2.5)	7.85(6.4)	7.75(7.7)	7.40(6.1)	8.49(2.6)
P09055	Integrin beta-1	Itgb1	1.55(20.4)	3.96(5.5)	4.62(12.5)	4.93(11.1)	4.85(22.8)	4.31(3.9)	3.84(18.2)	4.15(22.9)	3.30(18.8)	4.98(17.5)
O70309	Integrin beta-5	Itgb5		2.05(12.9)	3.82(7.1)	3.26(6.4)	2.10(10.6)		3.42(18.1)	3.02(12.7)	3.05(17.7)	2.57(15.6)
Q91WD5	NADH dehydrogenase [ubiquinone] iron-sulfur protein 2, mitochondrial	Ndufs2	3.24(16.8)	2.14(13.1)	2.51(12.5)	2.10(16.0)	2.90(18.1)	2.74(14.4)	1.66(14.8)	1.01(16.4)	2.35(21.3)	3.44(7.9)
Q9DCT2	NADH dehydrogenase [ubiquinone] iron-sulfur protein 3, mitochondrial	Ndufs3	3.02(20.1)	3.74(16.0)	1.82(10.5)	1.60(10.2)	1.60(13.9)	3.62(17.9)	2.04(8.9)	1.30(18.9)	1.33(1.1)	2.68(12.8)
O35468	Protein Wnt-9b	Wnt9b	1.04(19.2)	1.86(16.1)	2.04(14.7)	2.83(14.1)						
P63001	Ras-related C3 botulinum toxin substrate 1	Rac1	2.34(22.0)	4.71(22.6)	5.40(7.5)	4.84(16.0)	3.77(17.2)	2.80(21.7)	4.62(0.3)	2.96(16.2)	3.87(19.1)	3.57(15.9)
Q8K2B3	Succinate dehydrogenase [ubiquinone] flavoprotein subunit, mitochondrial	Sdha	4.08(14.4)	3.30(18.9)	2.82(6.8)	3.42(0.4)	2.60(12.1)	5.05(16.5)	3.04(13.5)	2.59(6.4)	2.33(22.1)	4.29(21.2)
Q93092	Transaldolase	Taldo1	4.45(18.8)	6.53(14.4)	6.83(10.8)	6.33(12.3)	5.38(6.8)	4.77(5.2)	6.05(8.4)	5.62(5.1)	5.88(2.0)	5.54(3.5)
Q9QUI0	Transforming protein RhoA	Rhoa	1.55(14.4)	4.37(18.9)	5.20(9.1)	6.02(5.5)	3.77(13.2)	3.93(16.2)	4.50(0.1)	3.99(2.2)	3.96(15.6)	4.21(14.6)
Q9D4D4	Transketolase-like protein 2	Tktl2	2.04(14.7)	2.64(11.7)	2.89(15.4)	3.51(16.5)	1.82(16.2)	1.21(20.1)	3.13(17.7)	2.34(22.4)	2.74(8.2)	2.86(5.4)
P20152	Vimentin	Vim	7.84(13.6)	8.68(0.7)	9.37(2.1)	9.50(5.2)	10.30(6.3)	8.75(4.4)	8.37(9.9)	8.73(6.3)	8.33(8.4)	9.51(5.7)

*Quantitative value is log2(protein area/ total protein area) X 10^6^.

