# Supplementary material for: Comparative Proteomic Analysis in Scar-Free Skin Regeneration in Acomys cahirinus and Scarring Mus musculus
Source: Sci Rep. 2020 Jan 13;10:166. doi: 10.1038/s41598-019-56823-y (PMC6957500; doi:10.1038/s41598-019-56823-y)
Supplement: Supplementary file 1 — Supplementary Dataset 1. [file 41598_2019_56823_MOESM1_ESM.docx]

**SUPPLEMENTARY DATA**

Comparative Proteomic Analysis in Scar-Free Skin Regeneration in *Acomys* *cahirinus* and Scarring *Mus* *musculus*

Jung Hae Yoon**^1^**, Kun Cho**^2^**, Timothy J. Garrett**^3^**, Paul Finch^4^ and Malcolm Maden**^5^***

**^1^** Department of Biology & UF Genetics Institute, 2033 Mowry Road, University of Florida, Gainesville, Florida 32610, USA

**^2^** Biomedical Omics Group, Korea Basic Science Institute, Ochang 863-883, Republic of Korea

**^3^** Department of Pathology, Immunology, and Laboratory Medicine, University of Florida, Gainesville, Florida, USA.

**^4^** School of Biological Sciences, Royal Holloway, University of London, Egham, Surrey, UK

**^5^*** Department of Biology & UF Genetics Institute, 2033 Mowry Road, University of Florida, Gainesville, Florida 32610, USA

**Corresponding author**: Malcolm Maden, Department of Biology & UF Genetics Institute, rm 415 Cancer Genetics Research Center, 2033 Mowry Road, University of Florida, Gainesville Florida 32610, USA.

Tel: 352-273- 7875, Fax 352-392-3704, email malcmaden[@ufl.edu](mailto:junghae.yoon@ufl.edu)

**Figure S1**: Workflow for proteomics study of *Acomys* and *Mus* skin.

**Figure S2**: Full image of the membrane probed for proteins shown in *Acomys* and *Mus* skin.

**Figure S3**: The boxplot representation on the Coefficient of variation (CV) of three biological replicates from *Acomys* and *Mus* at various times (0, 3, 5, 7, and 14 days).

**Table S1:** Comparisons of the known *Acomys cahirinus* protein sequences with proteins from *Mus musculus*.

**Table S2:** The list of proteins identified in *Acomys* and *Mus* skin at day 0.

**Table S3:** The proteins involved in various biological pathways in *Acomys* and *Mus.*

**Table S4:** Identified cytoskeletal and cuticular keratins from *Acomys* and *Mus* associated with wound healing over 14 days*.*

**Table S5:** Identified serine protease inhibitors (SERPINs) from *Acomys* and *Mus* associated with wound healing over 14 days*.*

**Figure S1**: Workflow for proteomics study of *Acomys* and *Mus* skin.


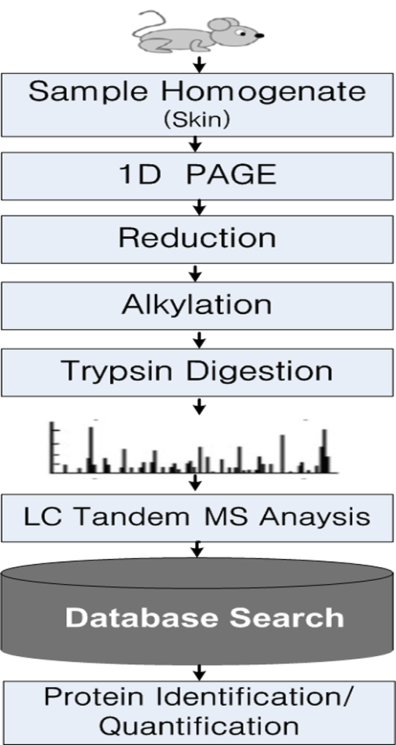


**Figure S2**: Full image of the membranes probed for proteins shown in *Acomys* and *Mus* skin.

Skin lysates before and after wounding were used to identify proteins. Proteins separated on 4-12% gradient SDS-PAGE gels (Invitrogen) were transferred to nitrocellulose membrane (Whatman), blocked with 5% skim milk in 0.1% Tween 20 / Tris-buffered saline and incubated with primary antibodies overnight. Membranes were incubated with appropriate horseradish peroxidase-conjugated secondary antibodies (Jackson immune Research) and enhanced chemiluminescence reagents (Pierce). Signal was detected using FluorChem E (Proteinsimple CA, USA). Approximate molecular weight of bands on Western blots are 130 kDa for Collagen I, 138 kDa for Collagen III, 340 kDa for Collagen XII, 11kDa for S100a8, 92kDa for MMP9, 23kDa for TIMP1 and 28kDa for 14-3-3. GAPDH (37kDa) or actin (42kDa) were used as a loading control.


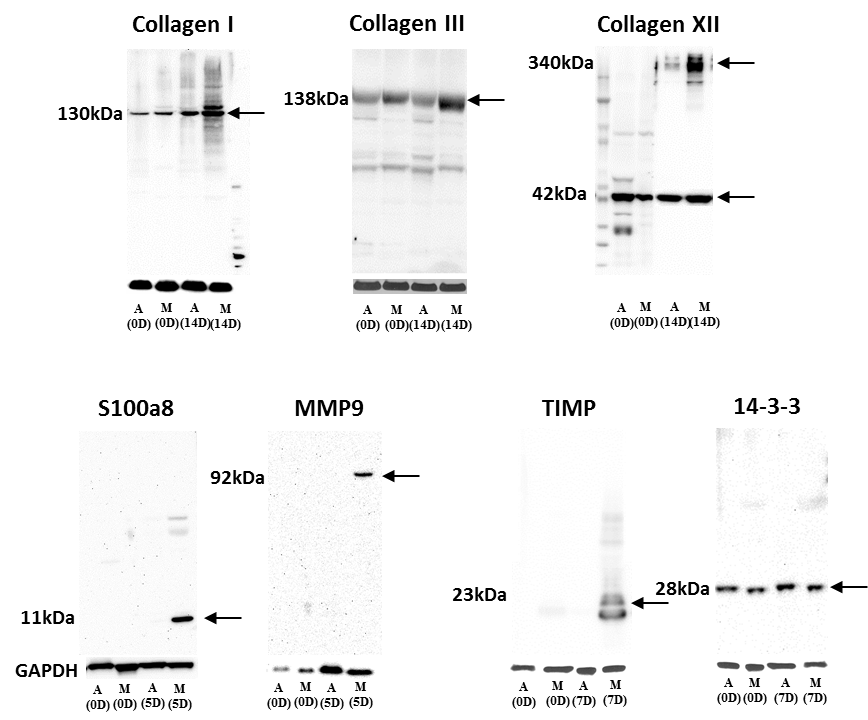


**Figure S3**: The boxplot representation of the CV values of *Acomys* (A) and *Mus* (B) from three biological replicates at five different time points; *Acomys* 0, 3, 5, 7 and14 days present as A0, A3, A5, A7 and A14, correspondingly, M0, M3, M5, M7, M14 for *Mus*. The coefficient of variance was calculated as follows: 100*(standard deviation/average) and reported. The boxed region indicates the range of values containing 20% of points around the median. Outliers with respect to CV are shown by open circles.

**A**

**
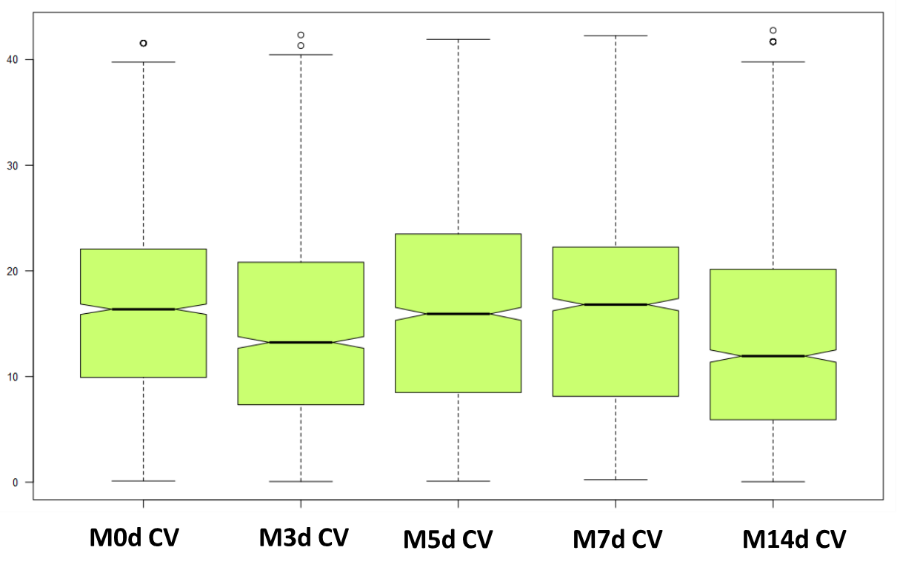
**

B

**
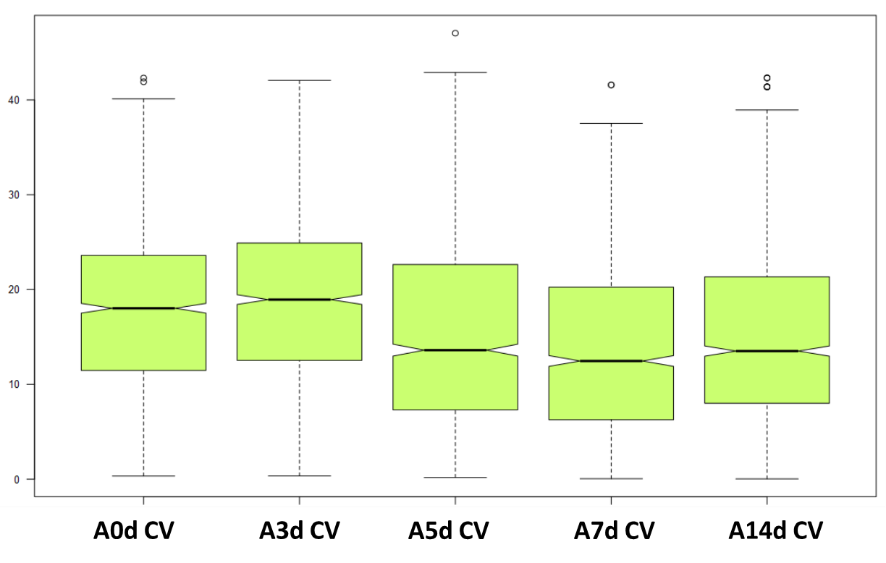
**

| **Table S1.** Comparison of the known *Acomys cahirinus* protein sequences with proteins from *Mus musculus*. |
| --- |
| **Query #1: 720921A insulin [Acomys cahirinus] Query ID: lcl\|Query_8004 Length:51** |
| **Sequence ID: P01325.1 Length: 108** |
|  |
| Query 22 FVNQHLCGSHLVEALYLVCGERGFFYTPKS 51 |
| FV QHLCG HLVEALYLVCGERGFFYTPKS |
| Sbjct 25 FVKQHLCGPHLVEALYLVCGERGFFYTPKS 54 |
|  |
| Query 1 GIVDQCCTSICSLYQLENYCN 21 |
| GIVDQCCTSICSLYQLENYCN |
| Sbjct 88 GIVDQCCTSICSLYQLENYCN 108 |
|  |
| **Query #2: QAT96945.1 recombination activating protein 1, partial [Acomys cahirinus] Query ID: lcl\|Query_8005 Length: 322** |
| **Sequence ID: P15919.2 Length: 1040** |
|  |
| Query 1 GPAVPEKAVRFSFTVMRITIEHGSQSVKVFEEPKPNSELCCXPLCLMLADESDHETLTAI 60 |
| GPAVPEKAVRFSFTVMRITIEHGSQ+VKVFEEPKPNSELCC PLCLMLADESDHETLTAI |
| Sbjct 612 GPAVPEKAVRFSFTVMRITIEHGSQNVKVFEEPKPNSELCCKPLCLMLADESDHETLTAI 671 |
|  |
| Query 61 LSPLIAEREAMKSSELMXEMGGILRTFKFIFRGTGYDEKLVREVEGXEASGSVYICTLCD 120 |
| LSPLIAEREAMKSSEL EMGGI RTFKFIFRGTGYDEKLVREVEG EASGSVYICTLCD |
| Sbjct 672 LSPLIAEREAMKSSELTLEMGGIPRTFKFIFRGTGYDEKLVREVEGLEASGSVYICTLCD 731 |
|  |
| Query 121 ATRLEASQNLVFHSITRSHAENLERYEVWRSNPYHESVEELRDRVKGVSAKPFIETVPSI 180 |
| TRLEASQNLVFHSITRSHAENL+RYEVWRSNPYHESVEELRDRVKGVSAKPFIETVPSI |
| Sbjct 732 TTRLEASQNLVFHSITRSHAENLQRYEVWRSNPYHESVEELRDRVKGVSAKPFIETVPSI 791 |
|  |
| Query 181 DALHCDIGNAAEFYKIFQLEIGEVYKNPNASKEERKRWQATLDKHLRKRMNLKPIMRMNG 240 |
| DALHCDIGNAAEFYKIFQLEIGEVYK+PNASKEERKRWQATLDKHLRKRMNLKPIMRMNG |
| Sbjct 792 DALHCDIGNAAEFYKIFQLEIGEVYKHPNASKEERKRWQATLDKHLRKRMNLKPIMRMNG 851 |
|  |
| Query 241 NFARKLMTQETVDAVCELIPSEERHEALRELMDLYLKMKPVWRSSCPAKECPESLCQYSF 300 |
| NFARKLMTQETVDAVCELIPSEERHEALRELMDLYLKMKPVWRSSCPAKECPESLCQYSF |
| Sbjct 852 NFARKLMTQETVDAVCELIPSEERHEALRELMDLYLKMKPVWRSSCPAKECPESLCQYSF 911 |
|  |
| Query 301 NSQRFAELLSTKFKYRYEGKIT 322 |
| NSQRFAELLSTKFKYRYEGKIT |
| Sbjct 912 NSQRFAELLSTKFKYRYEGKIT 933 |
|  |
| **Query #3: Q9QY94.3 Glutamine synthetase [Acomys cahirinus] Query ID: lcl\|Query_8006 Length: 373** |
| **Sequence ID: P15105.6 Length: 373** |
|  |
| Query 1 MATSASSHLNKGIKQMYMSLPQGEKVQAMYIWVDGTGEGLRCKTRTLDCEPKCVEELPEW 60 |
| MATSASSHLNKGIKQMYMSLPQGEKVQAMYIWVDGTGEGLRCKTRTLDCEPKCVEELPEW |
| Sbjct 1 MATSASSHLNKGIKQMYMSLPQGEKVQAMYIWVDGTGEGLRCKTRTLDCEPKCVEELPEW 60 |
|  |
| Query 61 NFDGSSTFQSEGSNSDMYLSPVAMFRDPFRKEPNKLVFCEVFKYNRKPAETNLRHSCKRI 120 |
| NFDGSSTFQSEGSNSDMYL PVAMFRDPFRK+PNKLV CEVFKYNRKPAETNLRH CKRI |
| Sbjct 61 NFDGSSTFQSEGSNSDMYLHPVAMFRDPFRKDPNKLVLCEVFKYNRKPAETNLRHICKRI 120 |
|  |
| Query 121 MDMVSNQHPWFGMEQEYTLMGTDGHPFGWPSNGFPGPQGPYYCGVGADKAYGRDIVEAHY 180 |
| MDMVSNQHPWFGMEQEYTLMGTDGHPFGWPSNGFPGPQGPYYCGVGADKAYGRDIVEAHY |
| Sbjct 121 MDMVSNQHPWFGMEQEYTLMGTDGHPFGWPSNGFPGPQGPYYCGVGADKAYGRDIVEAHY 180 |
|  |
| Query 181 RACLYAGVKITGTNAEVMPAQWEFQIGPCEGIRMGDHLWVARFILHRVCEDFGVIATFDP 240 |
| RACLYAGVKITGTNAEVMPAQWEFQIGPCEGIRMGDHLW+ARFILHRVCEDFGVIATFDP |
| Sbjct 181 RACLYAGVKITGTNAEVMPAQWEFQIGPCEGIRMGDHLWIARFILHRVCEDFGVIATFDP 240 |
|  |
| Query 241 KPIPGNWNGAGCHTNFSTKAMREENGLKYIEEAIDKLSKRHQYHIRAYDPKGGLDNARRL 300 |
| KPIPGNWNGAGCHTNFSTKAMREENGLK IEEAIDKLSKRHQYHIRAYDPKGGLDNARRL |
| Sbjct 241 KPIPGNWNGAGCHTNFSTKAMREENGLKCIEEAIDKLSKRHQYHIRAYDPKGGLDNARRL 300 |
|  |
| Query 301 TGFHETSNINDFSAGVANRGASIRIPRTVGQEKRGYFEDRRPSANCDPYAVTEAIVRTCL 360 |
| TGFHETSNINDFSAGVANRGASIRIPRTVGQEK+GYFEDRRPSANCDPYAVTEAIVRTCL |
| Sbjct 301 TGFHETSNINDFSAGVANRGASIRIPRTVGQEKKGYFEDRRPSANCDPYAVTEAIVRTCL 360 |
|  |
| Query 361 LNETGNEPFQYKN 373 |
| LNETG+EPFQYKN |
| Sbjct 361 LNETGDEPFQYKN 373 |
|  |
| **Query #4: QAT97086.1 cytochrome b, partial [Acomys cahirinus] Query ID: lcl\|Query_8007 Length: 339** |
| **Sequence ID: P00158.1 Length: 381** |
|  |
| Query 1 MRKTHPLLKIINHAFVDLPAPSNISSWWNFGSLLGVCLVIQILTGLFLAMHYTSDTMTAF 60 |
| MRKTHPL KIINH+F+DLPAPSNISSWWNFGSLLGVCL++QI+TGLFLAMHYTSDTMTAF |
| Sbjct 4 MRKTHPLFKIINHSFIDLPAPSNISSWWNFGSLLGVCLMVQIITGLFLAMHYTSDTMTAF 63 |
|  |
| Query 61 SSVTHICRDVNYGWLIRYLHANGASMFFVCLFMHVGRGIYYGSYTFTETWNLGIILLFAV 120 |
| SSVTHICRDVNYGWLIRY+HANGASMFF+CLF+HVGRG+YYGSYTF ETWN+G++LLFAV |
| Sbjct 64 SSVTHICRDVNYGWLIRYMHANGASMFFICLFLHVGRGLYYGSYTFMETWNIGVLLLFAV 123 |
|  |
| Query 121 MATAFMGYVLPWGQMSFWGATVITNLLSAIPYIGTDLVEWIWGGFSVDKATLTRFFALHF 180 |
| MATAFMGYVLPWGQMSFWGATVITNLLSAIPYIGT LVEWIWGGFSVDKATLTRFFA HF |
| Sbjct 124 MATAFMGYVLPWGQMSFWGATVITNLLSAIPYIGTTLVEWIWGGFSVDKATLTRFFAFHF 183 |
|  |
| Query 181 ILPFIIAALAMVHLLFLHETGSNNPTGVNSDADKIPFHPYYTIKDLLGVFLMLLVLLLLV 240 |
| ILPFIIAALA+VHLLFLHETGSNNPTG+NSDADKIPFHPYYTIKD+LG+ +M L+L+ LV |
| Sbjct 184 ILPFIIAALAIVHLLFLHETGSNNPTGLNSDADKIPFHPYYTIKDILGILIMFLILMTLV 243 |
|  |
| Query 241 LFSPDLLGDPDNYTPANPLNTPPHIKPEWYFLFAYAILRSIPNKLGGVLALVMSILILIF 300 |
| LF PD+LGDPDNY PANPLNTPPHIKPEWYFLFAYAILRSIPNKLGGVLAL++SILIL |
| Sbjct 244 LFFPDMLGDPDNYMPANPLNTPPHIKPEWYFLFAYAILRSIPNKLGGVLALILSILILAL 303 |
|  |
| Query 301 MPLTHTSKQRSLMFRPISQAMFWILTANLLILTWIGGQP 339 |
| MP HTSKQRSLMFRPI+Q ++WIL ANLLILTWIGGQP |
| Sbjct 304 MPFLHTSKQRSLMFRPITQILYWILVANLLILTWIGGQP 342 |
|  |
| **Query #5: QAT96957.1 recombination activating protein 1, partial [Acomys cahirinus] Query ID: lcl\|Query_8008 Length: 362** |
| **Sequence ID: P15919.2 Length: 1040** |
|  |
| Query 1 SQELEDYLNGPFTVVVKESCDGMGDVSEKHGSGPAVPEKAVRFSFTVMRITIEHGSQSVK 60 |
| SQ+L+DYLNGPFTVVVKESCDGMGDVSEKHGSGPAVPEKAVRFSFTVMRITIEHGSQ+VK |
| Sbjct 580 SQDLDDYLNGPFTVVVKESCDGMGDVSEKHGSGPAVPEKAVRFSFTVMRITIEHGSQNVK 639 |
|  |
| Query 61 VFEEPKPNSELCCKPLCLMLADESDHETLTAILSPLIAEREAMKSSELMLEMGGILRTFK 120 |
| VFEEPKPNSELCCKPLCLMLADESDHETLTAILSPLIAEREAMKSSEL LEMGGI RTFK |
| Sbjct 640 VFEEPKPNSELCCKPLCLMLADESDHETLTAILSPLIAEREAMKSSELTLEMGGIPRTFK 699 |
|  |
| Query 121 FIFRGTGYDEKLVREVEGLEASGSVYICTLCDATRLEASQNLVFHSITRSHAENLERYEV 180 |
| FIFRGTGYDEKLVREVEGLEASGSVYICTLCD TRLEASQNLVFHSITRSHAENL+RYEV |
| Sbjct 700 FIFRGTGYDEKLVREVEGLEASGSVYICTLCDTTRLEASQNLVFHSITRSHAENLQRYEV 759 |
|  |
| Query 181 WRSNPYHESVEELRDRVKGVSAKPFIETVPSIDALHCDIGNAAEFYKIFQLEIGEVYKNP 240 |
| WRSNPYHESVEELRDRVKGVSAKPFIETVPSIDALHCDIGNAAEFYKIFQLEIGEVYK+P |
| Sbjct 760 WRSNPYHESVEELRDRVKGVSAKPFIETVPSIDALHCDIGNAAEFYKIFQLEIGEVYKHP 819 |
|  |
| Query 241 NASKEERKRWQATLDKHLRKRMNLKPIMRMNGNFARKLMTQETVDAVCELIPSEERHEAL 300 |
| NASKEERKRWQATLDKHLRKRMNLKPIMRMNGNFARKLMTQETVDAVCELIPSEERHEAL |
| Sbjct 820 NASKEERKRWQATLDKHLRKRMNLKPIMRMNGNFARKLMTQETVDAVCELIPSEERHEAL 879 |
|  |
| Query 301 RELMDLYLKMKPVWRSSCPAKECPESLCQYSFNSQRFAELLSTKFKYRYEGKITNYFHKT 360 |
| RELMDLYLKMKPVWRSSCPAKECPESLCQYSFNSQRFAELLSTKFKYRYEGKITNYFHKT |
| Sbjct 880 RELMDLYLKMKPVWRSSCPAKECPESLCQYSFNSQRFAELLSTKFKYRYEGKITNYFHKT 939 |
|  |
| Query 361 LA 362 |
| LA |
| Sbjct 940 LA 941 |
|  |
| **Query #6: QAT96896.1 interphotoreceptor retinoid binding protein, partial [Acomys cahirinus] lcl\|Query_8009 Length: 273** |
| **Sequence ID: P49194.3 Length: 1234** |
|  |
| Query 1 HCAGGHVSGIPYVVSYLXPGNTVMHVDTIYDRPSNTTTEIWTLPRVLGERYSADKDVVVL 60 |
| HC+GGH SGIPYV+SYL PGNTVMHVDT+YDRPSNTTTEIWTLP VLGERYSADKDVVVL |
| Sbjct 171 HCSGGHFSGIPYVISYLHPGNTVMHVDTVYDRPSNTTTEIWTLPEVLGERYSADKDVVVL 230 |
|  |
| Query 61 TSGRTGGVAEDIAYILKQMRRAIVVGERTEGGALDLQKLRIGKSNFFLTVPVSRSLGPLG 120 |
| TSG TGGVAEDIAYILKQMRRAIVVGERTEGGALDLQKLRIG+SNFFLTVPVSRSLGPLG |
| Sbjct 231 TSGHTGGVAEDIAYILKQMRRAIVVGERTEGGALDLQKLRIGQSNFFLTVPVSRSLGPLG 290 |
|  |
| Query 121 GGGQTWEGSGVLPCVGTPAEQALEKALAILTLRRALPGVVLRLQEALQDYYTLVDRVPGL 180 |
| GGGQTWEGSGVLPCVGTPAEQALEKALAILTLRRALPGVVLRLQEALQDYYTLVDRVPGL |
| Sbjct 291 GGGQTWEGSGVLPCVGTPAEQALEKALAILTLRRALPGVVLRLQEALQDYYTLVDRVPGL 350 |
|  |
| Query 181 LHHLASMDYSAVVSEEDLVTKLNAGLQAVSEDPRLLVRATGPRETSCRPETGSNEPPAAV 240 |
| LHHLASMDYSAVVSEEDLVTKLNAGLQAVSEDPRLLVRATGPR++S RPETG NE PAA |
| Sbjct 351 LHHLASMDYSAVVSEEDLVTKLNAGLQAVSEDPRLLVRATGPRDSSSRPETGPNESPAAT 410 |
|  |
| Query 241 SEVPEEEAARRSLVDSVFQVSVLPGNVGYLRFD 273 |
| EVP EE ARR+LVDSVFQVSVLPGNVGYLRFD |
| Sbjct 411 PEVPTEEDARRALVDSVFQVSVLPGNVGYLRFD 443 |
|  |
| **Query #7: QAT96887.1 interphotoreceptor retinoid binding protein, partial [Acomys sp. B (cahirinus group)] Length: 397** |
| **Sequence ID: P49194.3 Length: 1234** |
|  |
| Query 1 MKSREILGISDPQTLAQVLTAGVQSSLNDPRLLISYEPSTLEAPQQVPELTNLTREDLLA 60 |
| MKS EILGISDPQTLAQVLTAGVQSSL+DPRL ISYEPSTLEAPQQ P LTNLTRE+LLA |
| Sbjct 56 MKSHEILGISDPQTLAQVLTAGVQSSLSDPRLFISYEPSTLEAPQQAPVLTNLTREELLA 115 |
|  |
| Query 61 RIQRNIHHEVLEGNVGYLRVDDLPGQEVLSELGEFLVTHVWRQLMSTSSLVLDLRHCAGG 120 |
| +IQRNI HEVLEGNVGYLRVDDLPGQEVLSELGEFLV+HVWRQLM TSSLVLDLRHC+GG |
| Sbjct 116 QIQRNIRHEVLEGNVGYLRVDDLPGQEVLSELGEFLVSHVWRQLMGTSSLVLDLRHCSGG 175 |
|  |
| Query 121 HVSGIPYVVSYLHPGNTVMHVDTIYDRPSNTTTEIWTLPRVLGERYSADKDVVVLTSGRT 180 |
| H SGIPYV+SYLHPGNTVMHVDT+YDRPSNTTTEIWTLP VLGERYSADKDVVVLTSG T |
| Sbjct 176 HFSGIPYVISYLHPGNTVMHVDTVYDRPSNTTTEIWTLPEVLGERYSADKDVVVLTSGHT 235 |
|  |
| Query 181 GGVAEDIAYILKQMRRAIVVGERTEGGALDLQKLRIGKSNFFLTVPVSRSLGPLGGGGQT 240 |
| GGVAEDIAYILKQMRRAIVVGERTEGGALDLQKLRIG+SNFFLTVPVSRSLGPLGGGGQT |
| Sbjct 236 GGVAEDIAYILKQMRRAIVVGERTEGGALDLQKLRIGQSNFFLTVPVSRSLGPLGGGGQT 295 |
|  |
| Query 241 WEGSGVLPCVGTPAEQALEKALAILTLRRALPGVVLRLQEALQDYYTLVDRVPGLLHHLA 300 |
| WEGSGVLPCVGTPAEQALEKALAILTLRRALPGVVLRLQEALQDYYTLVDRVPGLLHHLA |
| Sbjct 296 WEGSGVLPCVGTPAEQALEKALAILTLRRALPGVVLRLQEALQDYYTLVDRVPGLLHHLA 355 |
|  |
| Query 301 SMDYSAVVSEEDLVTKLNAGLQAVSEDPRLLVRATGPRETSCRPETGSNEPPAAVSEVPE 360 |
| SMDYSAVVSEEDLVTKLNAGLQAVSEDPRLLVRATGPR++S RPETG NE PAA EVP |
| Sbjct 356 SMDYSAVVSEEDLVTKLNAGLQAVSEDPRLLVRATGPRDSSSRPETGPNESPAATPEVPT 415 |
|  |
| Query 361 EEAARRSLVDSVFQVSVLPGNVGYLRFDRFADAPVL 396 |
| EE ARR+LVDSVFQVSVLPGNVGYLRFDRFADA VL |
| Sbjct 416 EEDARRALVDSVFQVSVLPGNVGYLRFDRFADAAVL 451 |
|  |
| **Query #8: P01324.1 Insulin [Acomys cahirinus] Query ID: lcl\|Query_8011 Length: 51** |
| **Sequence ID: P01325.1 Length: 108** |
|  |
| Query 1 FVBQHLCGSHLVEALYLVCGERGFFYTPKS------------------------------ 30 |
| FV QHLCG HLVEALYLVCGERGFFYTPKS |
| Sbjct 25 FVKQHLCGPHLVEALYLVCGERGFFYTPKSRREVEDPQVEQLELGGSPGDLQTLALEVAR 84 |
|  |
| Query 31 ---GIVDQCCTSICSLYQLENYCN 51 |
| GIVDQCCTSICSLYQLENYCN |
| Sbjct 85 QKRGIVDQCCTSICSLYQLENYCN 108 |
|  |
| **Query #9: Q9WTT5.1 Ribonuclease pancreatic [Acomys cahirinus] Query ID: lcl\|Query_8012 Length: 149** |
| **Sequence ID: Q9QYX2.1 Length: 149** |
|  |
| Query 1 MGLEKSLILLPLLVLVLAWVQPSLGKETPAMKFERQHMDSAGSSSSSPTYCNQMMKRREM 60 |
| MGLEKSL+L PL VL+L WVQPSLG+E+ A KF+RQHMD AGSSS+SPTYCNQMMKRR+M |
| Sbjct 1 MGLEKSLMLFPLFVLLLGWVQPSLGRESSAQKFQRQHMDPAGSSSNSPTYCNQMMKRRDM 60 |
|  |
| Query 61 TKGSCKRVNTFVHEPLADVQAVCSQKNVTCKNGKKNCYKSRSALTITDCRLKGNSKYPDC 120 |
| TKGSCK VNTFVHEPLADVQA+CSQ+NVTCKNGK NCYKS SAL ITDC LKGNSKYP+C |
| Sbjct 61 TKGSCKPVNTFVHEPLADVQAICSQENVTCKNGKSNCYKSSSALHITDCHLKGNSKYPNC 120 |
|  |
| Query 121 DYQTSHQQKHIIVACEGSPYVPVHFDASV 149 |
| DY+TS QK IIVACEG+PYVPVHFDA+V |
| Sbjct 121 DYKTSQYQKQIIVACEGNPYVPVHFDATV 149 |
|  |
| **Query #10: QAT96943.1 recombination activating protein 1, partial [Acomys johannis] Query ID: lcl\|Query_8013 Length: 344** |
| **Sequence ID: P15919.2 Length: 1040** |
|  |
| Query 1 NGPFTVVVKESCDGMGDVSEKHGSGPAVPEKAVRFSFTVMRITIEHGSQSVKVFEEPKPN 60 |
| NGPFTVVVKESCDGMGDVSEKHGSGPAVPEKAVRFSFTVMRITIEHGSQ+VKVFEEPKPN |
| Sbjct 588 NGPFTVVVKESCDGMGDVSEKHGSGPAVPEKAVRFSFTVMRITIEHGSQNVKVFEEPKPN 647 |
|  |
| Query 61 SELCCKPLCLMLADESDHETLTAILSPLIAEREAMKSSELMLEMGGILRTFKFIFRGTGY 120 |
| SELCCKPLCLMLADESDHETLTAILSPLIAEREAMKSSEL LEMGGI RTFKFIFRGTGY |
| Sbjct 648 SELCCKPLCLMLADESDHETLTAILSPLIAEREAMKSSELTLEMGGIPRTFKFIFRGTGY 707 |
|  |
| Query 121 DEKLVREVEGLEASGSVYICTLCDATRLEASQNLVFHSITRSHAENLERYEVWRSNPYHE 180 |
| DEKLVREVEGLEASGSVYICTLCD TRLEASQNLVFHSITRSHAENL+RYEVWRSNPYHE |
| Sbjct 708 DEKLVREVEGLEASGSVYICTLCDTTRLEASQNLVFHSITRSHAENLQRYEVWRSNPYHE 767 |
|  |
| Query 181 SVEELRDRVKGVSAKPFIETVPSIDALHCDIGNAAEFYKIFQLEIGEVYKNPNASKEERK 240 |
| SVEELRDRVKGVSAKPFIETVPSIDALHCDIGNAAEFYKIFQLEIGEVYK+PNASKEERK |
| Sbjct 768 SVEELRDRVKGVSAKPFIETVPSIDALHCDIGNAAEFYKIFQLEIGEVYKHPNASKEERK 827 |
|  |
| Query 241 RWQATLDKHLRKRMNLKPIMRMNGNFARKLMTQETVDAVCELIPSEERHEALRELMDLYL 300 |
| RWQATLDKHLRKRMNLKPIMRMNGNFARKLMTQETVDAVCELIPSEERHEALRELMDLYL |
| Sbjct 828 RWQATLDKHLRKRMNLKPIMRMNGNFARKLMTQETVDAVCELIPSEERHEALRELMDLYL 887 |
|  |
| Query 301 KMKPVWRSSCPAKECPESLCQYSFNSQRFAELLSTKFKYRYEGK 344 |
| KMKPVWRSSCPAKECPESLCQYSFNSQRFAELLSTKFKYRYEGK |
| Sbjct 888 KMKPVWRSSCPAKECPESLCQYSFNSQRFAELLSTKFKYRYEGK 931 |
|  |
| **Query #11: YP_007626822.1 cytochrome b [Acomys cahirinus] Query ID: lcl\|Query_8014 Length: 380** |
| **Sequence ID: P00158.1 Length: 381** |
|  |
| Query 1 MKIMRKTHPLLKIINHAFVDLPAPSNISSWWNFGSLLGVCLVMQILTGLFLAMHYTSDTM 60 |
| M MRKTHPL KIINH+F+DLPAPSNISSWWNFGSLLGVCL++QI+TGLFLAMHYTSDTM |
| Sbjct 1 MTNMRKTHPLFKIINHSFIDLPAPSNISSWWNFGSLLGVCLMVQIITGLFLAMHYTSDTM 60 |
|  |
| Query 61 TAFSSVTHICRDVNYGWLIRYLHANGASMFFVCLFMHVGRGIYYGSYTFTETWNLGVILL 120 |
| TAFSSVTHICRDVNYGWLIRY+HANGASMFF+CLF+HVGRG+YYGSYTF ETWN+GV+LL |
| Sbjct 61 TAFSSVTHICRDVNYGWLIRYMHANGASMFFICLFLHVGRGLYYGSYTFMETWNIGVLLL 120 |
|  |
| Query 121 FAVMATAFMGYVLPWGQMSFWGATVITNLLSAIPYIGTNLVEWIWGGFSVDKATLTRFFA 180 |
| FAVMATAFMGYVLPWGQMSFWGATVITNLLSAIPYIGT LVEWIWGGFSVDKATLTRFFA |
| Sbjct 121 FAVMATAFMGYVLPWGQMSFWGATVITNLLSAIPYIGTTLVEWIWGGFSVDKATLTRFFA 180 |
|  |
| Query 181 FHFILPFIIAALAMVHLLFLHETGSNNPTGVNSDADKIPFHPYYTIKDLLGVFLMLLILL 240 |
| FHFILPFIIAALA+VHLLFLHETGSNNPTG+NSDADKIPFHPYYTIKD+LG+ +M LIL+ |
| Sbjct 181 FHFILPFIIAALAIVHLLFLHETGSNNPTGLNSDADKIPFHPYYTIKDILGILIMFLILM 240 |
|  |
| Query 241 LLVLFSPDLLGDPDNYTPANPLNTPPHIKPEWYFLFAYAILRSIPNKLGGVLALVLSILI 300 |
| LVLF PD+LGDPDNY PANPLNTPPHIKPEWYFLFAYAILRSIPNKLGGVLAL+LSILI |
| Sbjct 241 TLVLFFPDMLGDPDNYMPANPLNTPPHIKPEWYFLFAYAILRSIPNKLGGVLALILSILI 300 |
|  |
| Query 301 LILMPLIHTSKQRSLMFRPISQAMFWILTANLLILTWIGGQPVEHPFIIIGQLASISYFS 360 |
| L LMP +HTSKQRSLMFRPI+Q ++WIL ANLLILTWIGGQPVEHPFIIIGQLASISYFS |
| Sbjct 301 LALMPFLHTSKQRSLMFRPITQILYWILVANLLILTWIGGQPVEHPFIIIGQLASISYFS 360 |
|  |
| Query 361 IILILMPISGLIENKMLKL 379 |
| IILILMPISG+IE+KMLKL |
| Sbjct 361 IILILMPISGIIEDKMLKL 379 |
|  |
| **Query #12: QAT96898.1 interphotoreceptor retinoid binding protein, partial [Acomys cahirinus] Query ID: lcl\|Query_8015 Length: 222** |
| **Sequence ID: P49194.3 Length: 1234** |
|  |
| Query 1 LGERYSADKDVVVLTSGRTGGVAEDIAYILKQMRRAIVVGERTEGGALDLQKLRIGKSNF 60 |
| LGERYSADKDVVVLTSG TGGVAEDIAYILKQMRRAIVVGERTEGGALDLQKLRIG+SNF |
| Sbjct 217 LGERYSADKDVVVLTSGHTGGVAEDIAYILKQMRRAIVVGERTEGGALDLQKLRIGQSNF 276 |
|  |
| Query 61 FLTVPVSRSLGPLGGGGQTWEGSGVLPCVGTPAEQALEKALAILTLRRALPGVVLRLQEA 120 |
| FLTVPVSRSLGPLGGGGQTWEGSGVLPCVGTPAEQALEKALAILTLRRALPGVVLRLQEA |
| Sbjct 277 FLTVPVSRSLGPLGGGGQTWEGSGVLPCVGTPAEQALEKALAILTLRRALPGVVLRLQEA 336 |
|  |
| Query 121 LQDYYTLVDRVPGLLHHLASMDYSAVVSEEDLVTKLNAGLQAVSEDPRLLVRATGPRETS 180 |
| LQDYYTLVDRVPGLLHHLASMDYSAVVSEEDLVTKLNAGLQAVSEDPRLLVRATGPR++S |
| Sbjct 337 LQDYYTLVDRVPGLLHHLASMDYSAVVSEEDLVTKLNAGLQAVSEDPRLLVRATGPRDSS 396 |
|  |
| Query 181 CRPETGSNEPPAAVSEVPEEEAARRSLVDSVFQVSVLPGNVG 222 |
| RPETG NE PAA EVP EE ARR+LVDSVFQVSVLPGNVG |
| Sbjct 397 SRPETGPNESPAATPEVPTEEDARRALVDSVFQVSVLPGNVG 438 |
|  |
| **Query #13: ACR24163.1 cytochrome b, partial [Acomys cahirinus] Query ID: lcl\|Query_8016 Length: 364** |
| **Sequence ID: P00158.1 Length: 381** |
|  |
| Query 1 PLLKIINHAFVDLPAPSNISSWWNFGSLLGVCLVIQILTGLFLAMHYTSDTMTAFSSVTH 60 |
| PL KIINH+F+DLPAPSNISSWWNFGSLLGVCL++QI+TGLFLAMHYTSDTMTAFSSVTH |
| Sbjct 9 PLFKIINHSFIDLPAPSNISSWWNFGSLLGVCLMVQIITGLFLAMHYTSDTMTAFSSVTH 68 |
|  |
| Query 61 ICRDVNYGWLIRYLHANGASMFFVCLFMHVGRGIYYGSYTFTETWNLGIILLFAVMATAF 120 |
| ICRDVNYGWLIRY+HANGASMFF+CLF+HVGRG+YYGSYTF ETWN+G++LLFAVMATAF |
| Sbjct 69 ICRDVNYGWLIRYMHANGASMFFICLFLHVGRGLYYGSYTFMETWNIGVLLLFAVMATAF 128 |
|  |
| Query 121 MGYVLPWGQMSFWGATVITNLLSAIPYIGTDLVEWIWGGFSVDKATLTRFFALHFILPFI 180 |
| MGYVLPWGQMSFWGATVITNLLSAIPYIGT LVEWIWGGFSVDKATLTRFFA HFILPFI |
| Sbjct 129 MGYVLPWGQMSFWGATVITNLLSAIPYIGTTLVEWIWGGFSVDKATLTRFFAFHFILPFI 188 |
|  |
| Query 181 IAALAMVHLLFLHETGSNNPTGVNSDADKIPFHPYYTIKDLLGVFLMLLVLLLLVLFSPD 240 |
| IAALA+VHLLFLHETGSNNPTG+NSDADKIPFHPYYTIKD+LG+ +M L+L+ LVLF PD |
| Sbjct 189 IAALAIVHLLFLHETGSNNPTGLNSDADKIPFHPYYTIKDILGILIMFLILMTLVLFFPD 248 |
|  |
| Query 241 LLGDPDNYTPANPLNTPPHIKPEWYFLFAYAILRSIPNKLGGVLALVMSILILIFMPLTH 300 |
| +LGDPDNY PANPLNTPPHIKPEWYFLFAYAILRSIPNKLGGVLAL++SILIL MP H |
| Sbjct 249 MLGDPDNYMPANPLNTPPHIKPEWYFLFAYAILRSIPNKLGGVLALILSILILALMPFLH 308 |
|  |
| Query 301 TSKQRSLMFRPISQAMFWILTANLLILTWIGGQPVEHPFIIIGQLASISYFSIILILMPI 360 |
| TSKQRSLMFRPI+Q ++WIL ANLLILTWIGGQPVEHPFIIIGQLASISYFSIILILMPI |
| Sbjct 309 TSKQRSLMFRPITQILYWILVANLLILTWIGGQPVEHPFIIIGQLASISYFSIILILMPI 368 |
|  |
| Query 361 SGLI 364 |
| SG+I |
| Sbjct 369 SGII 372 |
|  |
| **Query #14: ABW94925.1 melanocortin 4 receptor, partial [Acomys cahirinus] Query ID: lcl\|Query_8017 Length: 288** |
| **Sequence ID: P56450.3 Length: 332** |
|  |
| Query 1 DGGCYEQLFVSPEVFVTLGVISLLENILVIVAIAKNKNLHSPMYFFICSLAVADMLVSVS 60 |
| DGGCYEQLFVSPEVFVTLGVISLLENILVIVAIAKNKNLHSPMYFFICSLAVADMLVSVS |
| Sbjct 37 DGGCYEQLFVSPEVFVTLGVISLLENILVIVAIAKNKNLHSPMYFFICSLAVADMLVSVS 96 |
|  |
| Query 61 NGSETIVITLLNSTDTDAQXFTVNIDNVIDSVICSSLLASICSLLSIAVDRYFTIFYALQ 120 |
| NGSETIVITLLNSTDTDAQ FTVNIDNVIDSVICSSLLASICSLLSIAVDRYFTIFYALQ |
| Sbjct 97 NGSETIVITLLNSTDTDAQSFTVNIDNVIDSVICSSLLASICSLLSIAVDRYFTIFYALQ 156 |
|  |
| Query 121 YHNIMTVKRVGIIISCIWAACTVSGILFIIYSDSSAVIICLITMFFTMLVLMASLYVHMF 180 |
| YHNIMTV+RVGIIISCIWAACTVSG+LFIIYSDSSAVIICLI+MFFTMLVLMASLYVHMF |
| Sbjct 157 YHNIMTVRRVGIIISCIWAACTVSGVLFIIYSDSSAVIICLISMFFTMLVLMASLYVHMF 216 |
|  |
| Query 181 LMARLHIKRIAVLPGTGTIRQGANMKGAITLTILIGVFVVCWAPFFLHLLFYISCPQNPY 240 |
| LMARLHIKRIAVLPGTGTIRQG NMKGAITLTILIGVFVVCWAPFFLHLLFYISCPQNPY |
| Sbjct 217 LMARLHIKRIAVLPGTGTIRQGTNMKGAITLTILIGVFVVCWAPFFLHLLFYISCPQNPY 276 |
|  |
| Query 241 CVCFMSHFNLYLILIMCNAVIDPLIYALRSQELRKTFKEIICCYPLGG 288 |
| CVCFMSHFNLYLILIMCNAVIDPLIYALRSQELRKTFKEIIC YPLGG |
| Sbjct 277 CVCFMSHFNLYLILIMCNAVIDPLIYALRSQELRKTFKEIICFYPLGG 324 |
|  |
| **Query #15: CAB37993.1 cytochrome b, partial [Acomys cahirinus] Query ID: lcl\|Query_8018 Length: 380** |
| **Sequence ID: P00158.1 Length: 381** |
|  |
| Query 1 MKIMRKTHPLLKIINHAFVDLPAPSNISSWWNFGSLLGVCLVIQILTGLFLAMHYTSDTM 60 |
| M MRKTHPL KIINH+F+DLPAPSNISSWWNFGSLLGVCL++QI+TGLFLAMHYTSDTM |
| Sbjct 1 MTNMRKTHPLFKIINHSFIDLPAPSNISSWWNFGSLLGVCLMVQIITGLFLAMHYTSDTM 60 |
|  |
| Query 61 TAFSSVTHICRDVNYGWLIRYLHANGASMFFVCLFMHVGRGIYYGSYTFTETWNLGIILL 120 |
| TAFSSVTHICRDVNYGWLIRY+HANGASMFF+CLF+HVGRG+YYGSYTF ETWN+G++LL |
| Sbjct 61 TAFSSVTHICRDVNYGWLIRYMHANGASMFFICLFLHVGRGLYYGSYTFMETWNIGVLLL 120 |
|  |
| Query 121 FAVMATAFMGYVLPWGQMSFWGATVITNLLSAIPYIGTDLVEWIWGGFSVDKATLTRFFA 180 |
| FAVMATAFMGYVLPWGQMSFWGATVITNLLSAIPYIGT LVEWIWGGFSVDKATLTRFFA |
| Sbjct 121 FAVMATAFMGYVLPWGQMSFWGATVITNLLSAIPYIGTTLVEWIWGGFSVDKATLTRFFA 180 |
|  |
| Query 181 LHFILPFIIAALAMVHLLFLHETGSNNPTGVNSDADKIPFHPYYTIKDLLGVFLMLLVLL 240 |
| HFILPFIIAALA+VHLLFLHETGSNNPTG+NSDADKIPFHPYYTIKD+LG+ +M L+L+ |
| Sbjct 181 FHFILPFIIAALAIVHLLFLHETGSNNPTGLNSDADKIPFHPYYTIKDILGILIMFLILM 240 |
|  |
| Query 241 LLVLFSPDLLGDPDNYTPANPLNTPPHIKPEWYFLFAYAILRSIPNKLGGVLALVMSILI 300 |
| LVLF PD+LGDPDNY PANPLNTPPHIKPEWYFLFAYAILRSIPNKLGGVLAL++SILI |
| Sbjct 241 TLVLFFPDMLGDPDNYMPANPLNTPPHIKPEWYFLFAYAILRSIPNKLGGVLALILSILI 300 |
|  |
| Query 301 LIFMPLTHTSKQRSLMFRPISQAMFWILTANLLILTWIGGQPVEHPFIIIGQLASISYFS 360 |
| L MP HTSKQRSLMFRPI+Q ++WIL ANLLILTWIGGQPVEHPFIIIGQLASISYFS |
| Sbjct 301 LALMPFLHTSKQRSLMFRPITQILYWILVANLLILTWIGGQPVEHPFIIIGQLASISYFS 360 |
|  |
| Query 361 IILILMPISGLIENKMLKL 379 |
| IILILMPISG+IE+KMLKL |
| Sbjct 361 IILILMPISGIIEDKMLKL 379 |
|  |
| **Query #16: CAB41660.2 cytochrome b, partial [Acomys cahirinus] Query ID: lcl\|Query_8019 Length: 373** |
| **Sequence ID: P00158.1 Length: 381** |
|  |
| Query 1 MKIMRKTHPLLKIINHAFVDLPAPSNISSWWNFGSLLGVCLVIQILTGLFLAMHYTSDTM 60 |
| M MRKTHPL KIINH+F+DLPAPSNISSWWNFGSLLGVCL++QI+TGLFLAMHYTSDTM |
| Sbjct 1 MTNMRKTHPLFKIINHSFIDLPAPSNISSWWNFGSLLGVCLMVQIITGLFLAMHYTSDTM 60 |
|  |
| Query 61 TAFSSVTHICRDVNYGWLIRYLHANGASMFFVCLFMHVGRGIYYGSYTFTETWNLGIILL 120 |
| TAFSSVTHICRDVNYGWLIRY+HANGASMFF+CLF+HVGRG+YYGSYTF ETWN+G++LL |
| Sbjct 61 TAFSSVTHICRDVNYGWLIRYMHANGASMFFICLFLHVGRGLYYGSYTFMETWNIGVLLL 120 |
|  |
| Query 121 FAVMATAFMGYVLPWGQMSFWGATVITNLLSAIPYIGTNLVEWIWGGFSVDKATLTRFFA 180 |
| FAVMATAFMGYVLPWGQMSFWGATVITNLLSAIPYIGT LVEWIWGGFSVDKATLTRFFA |
| Sbjct 121 FAVMATAFMGYVLPWGQMSFWGATVITNLLSAIPYIGTTLVEWIWGGFSVDKATLTRFFA 180 |
|  |
| Query 181 FHFILPFIIAALAMVHLLFLHETGSNNPTGMNSDADKIPFHPYYTIKDLLGVFLMLSVLL 240 |
| FHFILPFIIAALA+VHLLFLHETGSNNPTG+NSDADKIPFHPYYTIKD+LG+ +M +L+ |
| Sbjct 181 FHFILPFIIAALAIVHLLFLHETGSNNPTGLNSDADKIPFHPYYTIKDILGILIMFLILM 240 |
|  |
| Query 241 LLVLFSPDLLGDPDNYTPANPLNTPPHIKPEWYFLFAYAILRSIPNKLGGVLALVLSILI 300 |
| LVLF PD+LGDPDNY PANPLNTPPHIKPEWYFLFAYAILRSIPNKLGGVLAL+LSILI |
| Sbjct 241 TLVLFFPDMLGDPDNYMPANPLNTPPHIKPEWYFLFAYAILRSIPNKLGGVLALILSILI 300 |
|  |
| Query 301 LAFMPLTHTSKQRSLMFRPISQTMFWILAANLLILTWIGGQPVEHPFIIIGQLASISYFS 360 |
| LA MP HTSKQRSLMFRPI+Q ++WIL ANLLILTWIGGQPVEHPFIIIGQLASISYFS |
| Sbjct 301 LALMPFLHTSKQRSLMFRPITQILYWILVANLLILTWIGGQPVEHPFIIIGQLASISYFS 360 |
|  |
| Query 361 IILILMPISG 370 |
| IILILMPISG |
| Sbjct 361 IILILMPISG 370 |
|  |
| **Query #17: CAB41661.1 cytochrome b, partial [Acomys cahirinus] Query ID: lcl\|Query_8020 Length: 380** |
| **Sequence ID: P00158.1 Length: 381** |
|  |
| Query 1 MKIMRKTHPLLKIINHAFVDLPAPSNISSWWNFGSLLGVCLVIQILTGLFLAMHYTSDTM 60 |
| M MRKTHPL KIINH+F+DLPAPSNISSWWNFGSLLGVCL++QI+TGLFLAMHYTSDTM |
| Sbjct 1 MTNMRKTHPLFKIINHSFIDLPAPSNISSWWNFGSLLGVCLMVQIITGLFLAMHYTSDTM 60 |
|  |
| Query 61 TAFSSVTHICRDVNYGWLIRYLHANGASMFFVCLFMHVGRGIYYGSYTFTETWNLGIILL 120 |
| TAFSSVTHICRDVNYGWLIRY+HANGASMFF+CLF+HVGRG+YYGSYTF ETWN+G++LL |
| Sbjct 61 TAFSSVTHICRDVNYGWLIRYMHANGASMFFICLFLHVGRGLYYGSYTFMETWNIGVLLL 120 |
|  |
| Query 121 FAVMATAFMGYVLPWGQMSFWGATVITNLLSAIPYIGTDLVEWIWGGFSVDKATLTRFFA 180 |
| FAVMATAFMGYVLPWGQMSFWGATVITNLLSAIPYIGT LVEWIWGGFSVDKATLTRFFA |
| Sbjct 121 FAVMATAFMGYVLPWGQMSFWGATVITNLLSAIPYIGTTLVEWIWGGFSVDKATLTRFFA 180 |
|  |
| Query 181 LHFILPFIIAALAMVHLLFLHETGSNNPTGVNSDADKIPFHPYYTIKDLLGVFLMLLVLL 240 |
| HFILPFIIAALA+VHLLFLHETGSNNPTG+NSDADKIPFHPYYTIKD+LG+ +M L+L+ |
| Sbjct 181 FHFILPFIIAALAIVHLLFLHETGSNNPTGLNSDADKIPFHPYYTIKDILGILIMFLILM 240 |
|  |
| Query 241 LLVLFSPDLLGDPDNYTPANPLNTPPHIKPEWYFLFAYAILRSIPNKLGGVLALVMSILI 300 |
| LVLF PD+LGDPDNY PANPLNTPPHIKPEWYFLFAYAILRSIPNKLGGVLAL++SILI |
| Sbjct 241 TLVLFFPDMLGDPDNYMPANPLNTPPHIKPEWYFLFAYAILRSIPNKLGGVLALILSILI 300 |
|  |
| Query 301 LIFMPLTHTSKQRSLMFRPISQAMFWILTANLLILTWIGGQPVEHPFIIIGQLASISYFS 360 |
| L MP HTSKQRSLMFRPI+Q ++WIL ANLLILTWIGGQPVEHPFIIIGQLASISYFS |
| Sbjct 301 LALMPFLHTSKQRSLMFRPITQILYWILVANLLILTWIGGQPVEHPFIIIGQLASISYFS 360 |
|  |
| Query 361 IILXXMPISGLIENKMLKL 379 |
| IIL MPISG+IE+KMLKL |
| Sbjct 361 IILILMPISGIIEDKMLKL 379 |
|  |
| **Query #18: CAG27723.1 interphotoreceptor retinoid binding protein, partial [Acomys cahirinus] Query ID: lcl\|Query_8021 Length: 423** |
| **Sequence ID: P49194.3 Length: 1234** |
|  |
| Query 1 PENLMGMQATIEQAMKSREILGISDPQTLAQVLTAGVQSSLNDPRLLISYEPSTLEAXQQ 60 |
| PENLMGMQA IEQAMKS EILGISDPQTLAQVLTAGVQSSL+DPRL ISYEPSTLEA QQ |
| Sbjct 42 PENLMGMQAAIEQAMKSHEILGISDPQTLAQVLTAGVQSSLSDPRLFISYEPSTLEAPQQ 101 |
|  |
| Query 61 VPELTNLTREDLLARIQRNIHHEVLEGNVGYLRVDDLPGQEVLSELGEFLVTHVWRQLMS 120 |
| P LTNLTRE+LLA+IQRNI HEVLEGNVGYLRVDDLPGQEVLSELGEFLV+HVWRQLM |
| Sbjct 102 APVLTNLTREELLAQIQRNIRHEVLEGNVGYLRVDDLPGQEVLSELGEFLVSHVWRQLMG 161 |
|  |
| Query 121 TSSLVLDLRHCAGGHVSGIPYVVSYLHPGNTVMHVDTIYDRPSNTTTEIWTLPRVLGERY 180 |
| TSSLVLDLRHC+GGH SGIPYV+SYLHPGNTVMHVDT+YDRPSNTTTEIWTLP VLGERY |
| Sbjct 162 TSSLVLDLRHCSGGHFSGIPYVISYLHPGNTVMHVDTVYDRPSNTTTEIWTLPEVLGERY 221 |
|  |
| Query 181 SADKDVVVLTSGRTGGVAEDIAYILKQMRRAIVVGERTEGGALDLQKLRIGKSNFFLTVP 240 |
| SADKDVVVLTSG TGGVAEDIAYILKQMRRAIVVGERTEGGALDLQKLRIG+SNFFLTVP |
| Sbjct 222 SADKDVVVLTSGHTGGVAEDIAYILKQMRRAIVVGERTEGGALDLQKLRIGQSNFFLTVP 281 |
|  |
| Query 241 VSRSLGPLGGGGQTWEGSGVLPCVGTPAEQALEKALAILTLRRALPGVVLRLQEALQDYY 300 |
| VSRSLGPLGGGGQTWEGSGVLPCVGTPAEQALEKALAILTLRRALPGVVLRLQEALQDYY |
| Sbjct 282 VSRSLGPLGGGGQTWEGSGVLPCVGTPAEQALEKALAILTLRRALPGVVLRLQEALQDYY 341 |
|  |
| Query 301 TLVDRVPGLLHHLASMDYSAVVSEEDLVTKLNAGLQAVSEDPRLLVRATGPRETSCRPET 360 |
| TLVDRVPGLLHHLASMDYSAVVSEEDLVTKLNAGLQAVSEDPRLLVRATGPR++S RPET |
| Sbjct 342 TLVDRVPGLLHHLASMDYSAVVSEEDLVTKLNAGLQAVSEDPRLLVRATGPRDSSSRPET 401 |
|  |
| Query 361 GSNEPPAAVSEVPEEEAARRSLVDSVFQVSVLPGNVGYLRFDRFADAPVLRALGPYVLHQ 420 |
| G NE PAA EVP EE ARR+LVDSVFQVSVLPGNVGYLRFDRFADA VL LGPYVL Q |
| Sbjct 402 GPNESPAATPEVPTEEDARRALVDSVFQVSVLPGNVGYLRFDRFADAAVLETLGPYVLKQ 461 |
|  |
| Query 421 VWE 423 |
| VWE |
| Sbjct 462 VWE 464 |
|  |
| **Query #19: CAB09426.1 cytochrome b, partial [Acomys cahirinus] Query ID: lcl\|Query_8022 Length: 380** |
| **Sequence ID: P00158.1 Length: 381** |
|  |
| Query 1 MKIMRKTHPLLKIINHAFVDLPAPSNISSWWNFGSLLGVCLVIQILTGLFLAMHYTSDTM 60 |
| M MRKTHPL KIINH+F+DLPAPSNISSWWNFGSLLGVCL++QI+TGLFLAMHYTSDTM |
| Sbjct 1 MTNMRKTHPLFKIINHSFIDLPAPSNISSWWNFGSLLGVCLMVQIITGLFLAMHYTSDTM 60 |
|  |
| Query 61 TAFSSVTHICRDVNYGWLIRYLHANGASMFFESLFMHVGRGIYYGSYTFTETWNLGIILL 120 |
| TAFSSVTHICRDVNYGWLIRY+HANGASMFF LF+HVGRG+YYGSYTF ETWN+G++LL |
| Sbjct 61 TAFSSVTHICRDVNYGWLIRYMHANGASMFFICLFLHVGRGLYYGSYTFMETWNIGVLLL 120 |
|  |
| Query 121 FAVMATAFMGYVLPWGQMSFWGATVITNLLSAIPYIGTNLVEWIWGGFSVDKATLTRFFA 180 |
| FAVMATAFMGYVLPWGQMSFWGATVITNLLSAIPYIGT LVEWIWGGFSVDKATLTRFFA |
| Sbjct 121 FAVMATAFMGYVLPWGQMSFWGATVITNLLSAIPYIGTTLVEWIWGGFSVDKATLTRFFA 180 |
|  |
| Query 181 FHFILPFIIAALAMVHLLFLHETGSNNPTGMNSDADKIPFHPYYTIKDLLGVFLMLSVLL 240 |
| FHFILPFIIAALA+VHLLFLHETGSNNPTG+NSDADKIPFHPYYTIKD+LG+ +M +L+ |
| Sbjct 181 FHFILPFIIAALAIVHLLFLHETGSNNPTGLNSDADKIPFHPYYTIKDILGILIMFLILM 240 |
|  |
| Query 241 LLVLFSPDLLGDPDNYTPANPLNTPPHIKPEWYFLFAYAILRSIPNKLGGVLALVLSILI 300 |
| LVLF PD+LGDPDNY PANPLNTPPHIKPEWYFLFAYAILRSIPNKLGGVLAL+LSILI |
| Sbjct 241 TLVLFFPDMLGDPDNYMPANPLNTPPHIKPEWYFLFAYAILRSIPNKLGGVLALILSILI 300 |
|  |
| Query 301 LAFMPLTHTSKQRSLMFRPISQTMFWILTANLLILTWIGGQPVEHPFIIIGQLASISYFS 360 |
| LA MP HTSKQRSLMFRPI+Q ++WIL ANLLILTWIGGQPVEHPFIIIGQLASISYFS |
| Sbjct 301 LALMPFLHTSKQRSLMFRPITQILYWILVANLLILTWIGGQPVEHPFIIIGQLASISYFS 360 |
|  |
| Query 361 IILILMPISGLIENKMLKL 379 |
| IILILMPISG+IE+KMLKL |
| Sbjct 361 IILILMPISGIIEDKMLKL 379 |
|  |
| **Query #20: ABR23643.1 prion protein, partial [Acomys cahirinus] Query ID: lcl\|Query_8023 Length: 245** |
| **Sequence ID: P04925.2 Length: 254** |
|  |
| Query 1 MANLGYWLLALFVTMWTDVGLCKKRPKPGGWNTGGSRYPGQGSPGGNRYPPQGGGTWGQP 60 |
| MANLGYWLLALFVTMWTDVGLCKKRPKPGGWNTGGSRYPGQGSPGGNRYPPQGG TWGQP |
| Sbjct 1 MANLGYWLLALFVTMWTDVGLCKKRPKPGGWNTGGSRYPGQGSPGGNRYPPQGG-TWGQP 59 |
|  |
| Query 61 HGGGWGQPHGGGWGQPHGGGWGQPHGGGWGQGGGTHSQWGKPSKPKTNMKHVAGAAAAGA 120 |
| HGGGWGQPHGG WGQPHGG WGQPHGGGWGQGGGTH+QW KPSKPKTN+KHVAGAAAAGA |
| Sbjct 60 HGGGWGQPHGGSWGQPHGGSWGQPHGGGWGQGGGTHNQWNKPSKPKTNLKHVAGAAAAGA 119 |
|  |
| Query 121 VVGGLGGYMLGSAMSRPMIHFGNDWEDRYYRENMYRYPNQVYYRPVDQYSNQNNFVHDCV 180 |
| VVGGLGGYMLGSAMSRPMIHFGNDWEDRYYRENMYRYPNQVYYRPVDQYSNQNNFVHDCV |
| Sbjct 120 VVGGLGGYMLGSAMSRPMIHFGNDWEDRYYRENMYRYPNQVYYRPVDQYSNQNNFVHDCV 179 |
|  |
| Query 181 NITIKQHTVTTTTKGENFTETDVKMMERVVEQMCVTQYQKESQAYYDGRRSSA-VLFSSP 239 |
| NITIKQHTVTTTTKGENFTETDVKMMERVVEQMCVTQYQKESQAYYDGRRSS+ VLFSSP |
| Sbjct 180 NITIKQHTVTTTTKGENFTETDVKMMERVVEQMCVTQYQKESQAYYDGRRSSSTVLFSSP 239 |
|  |
| Query 240 PVILLI 245 |
| PVILLI |
| Sbjct 240 PVILLI 245 |
|  |
| **Query #21: CAC37557.1 von Willebrand Factor, partial [Acomys cahirinus] Query ID: lcl\|Query_8024 Length: 385** |
| **Sequence ID: Q8CIZ8.2 Length: 2813** |
|  |
| Query 1 PTSSTTPYVENTPEPPLQSFSCSKLLDLVFLLDGSSSLSEAEFEKLKAFVMGTMXKLHIS 60 |
| P SSTTPYVE+TPEPPL +F CSKLLDLVFLLDGSS LSEAEFE LKAFV+G M +LHIS |
| Sbjct 1251 PVSSTTPYVEDTPEPPLHNFYCSKLLDLVFLLDGSSMLSEAEFEVLKAFVVGMMERLHIS 1310 |
|  |
| Query 61 XKRIRVAVVEYHDGSHAYIELKARKRPSXLRRITSQVKYAGSEVASTSEVLKYTLFQIFG 120 |
| KRIRVAVVEYHDGS AY+ELKARKRPS LRRITSQ+KY GS+VASTSEVLKYTLFQIFG |
| Sbjct 1311 QKRIRVAVVEYHDGSRAYLELKARKRPSELRRITSQIKYTGSQVASTSEVLKYTLFQIFG 1370 |
|  |
| Query 121 RIDRPEASRIALLLTASQEPSQTARTLTRYVQALKKKKVIVVPVGIGPRVSLKQIRLIER 180 |
| +IDRPEAS I LLLTASQEP + AR L RYVQ LKKKKVIV+PVGIGP SLKQIRLIE+ |
| Sbjct 1371 KIDRPEASHITLLLTASQEPPRMARNLVRYVQGLKKKKVIVIPVGIGPHASLKQIRLIEK 1430 |
|  |
| Query 181 QAPENKAFLLSGVDELEQKRSELINHLCDLVPGAPAPTQPPQVAQITVGPEISGVSSPGP 240 |
| QAPENKAFLLSGVDELEQ+R E++++LCDL P APAPTQPPQVA +TV P I+G+SSPGP |
| Sbjct 1431 QAPENKAFLLSGVDELEQRRDEIVSYLCDLAPEAPAPTQPPQVAHVTVSPGIAGISSPGP 1490 |
|  |
| Query 241 KRKSMVLDVVFVLEGSDKVGEANFNKSKEFLEEVIRRMDVGQGGIHITVLQYSYTVTVEY 300 |
| KRKSMVLDVVFVLEGSD+VGEANFNKSKEF+EEVI+RMDV I+VLQYSYTVT+EY |
| Sbjct 1491 KRKSMVLDVVFVLEGSDEVGEANFNKSKEFVEEVIQRMDVSPDATRISVLQYSYTVTMEY 1550 |
|  |
| Query 301 TFNEAQAKEDVLRHVREIRYHGGNRTNTGLALQYVSEHSFSPRQGDRQQAPNLVYMVTGN 360 |
| FN AQ+KE+VLRHVREIRY GGNRTNTG ALQY+SEHSFSP QGDR +APNLVYMVTGN |
| Sbjct 1551 AFNGAQSKEEVLRHVREIRYQGGNRTNTGQALQYLSEHSFSPSQGDRVEAPNLVYMVTGN 1610 |
|  |
| Query 361 PASDEIKRLPGDIQIVPIGVGPHAN 385 |
| PASDEIKRLPGDIQ+VPIGVGPHAN |
| Sbjct 1611 PASDEIKRLPGDIQVVPIGVGPHAN 1635 |
|  |
| **Query #22: CAB09425.1 cytochrome b, partial [Acomys cahirinus] Query ID: lcl\|Query_8025 Length: 380** |
| **Sequence ID: P00158.1 Length: 381** |
|  |
| Query 1 MKIMRKTHPLLKIINHAFVDLPAPSNISSWWNFGSLLGVCLVIQILTGLFLAMHYTSDTM 60 |
| M MRKTHPL KIINH+F+DLPAPSNISSWWNFGSLLGVCL++QI+TGLFLAMHYTSDTM |
| Sbjct 1 MTNMRKTHPLFKIINHSFIDLPAPSNISSWWNFGSLLGVCLMVQIITGLFLAMHYTSDTM 60 |
|  |
| Query 61 TAFSSVTHICRDVNYGWLIRYLHANGASMFFVCLFMHVGRGIYYGSYTFTETWNLGIILL 120 |
| TAFSSVTHICRDVNYGWLIRY+HANGASMFF+CLF+HVGRG+YYGSYTF ETWN+G++LL |
| Sbjct 61 TAFSSVTHICRDVNYGWLIRYMHANGASMFFICLFLHVGRGLYYGSYTFMETWNIGVLLL 120 |
|  |
| Query 121 FAVMATAFMGYVLPWGQMSFWGATVITNLLSAIPYIGTDLVEWIWGGFSVDKATLTRFFA 180 |
| FAVMATAFMGYVLPWGQMSFWGATVITNLLSAIPYIGT LVEWIWGGFSVDKATLTRFFA |
| Sbjct 121 FAVMATAFMGYVLPWGQMSFWGATVITNLLSAIPYIGTTLVEWIWGGFSVDKATLTRFFA 180 |
|  |
| Query 181 LHFILPFIIAALAMVHLLFLHETGSNNPSGVNSDADKIPFHPYYTIKDLLGVFLMLLVLL 240 |
| HFILPFIIAALA+VHLLFLHETGSNNP+G+NSDADKIPFHPYYTIKD+LG+ +M L+L+ |
| Sbjct 181 FHFILPFIIAALAIVHLLFLHETGSNNPTGLNSDADKIPFHPYYTIKDILGILIMFLILM 240 |
|  |
| Query 241 LLVLFSPDLLGDPDNYTPANPLNTPPHIKPEWYFLFAYAILRSIPNKLGGVLALVMSILI 300 |
| LVLF PD+LGDPDNY PANPLNTPPHIKPEWYFLFAYAILRSIPNKLGGVLAL++SILI |
| Sbjct 241 TLVLFFPDMLGDPDNYMPANPLNTPPHIKPEWYFLFAYAILRSIPNKLGGVLALILSILI 300 |
|  |
| Query 301 LIFMPLTHTSKQRSLMFRPISQAMFWILTANLLILTWIGGQPVEHPFIIIGQLASISYFS 360 |
| L MP HTSKQRSLMFRPI+Q ++WIL ANLLILTWIGGQPVEHPFIIIGQLASISYFS |
| Sbjct 301 LALMPFLHTSKQRSLMFRPITQILYWILVANLLILTWIGGQPVEHPFIIIGQLASISYFS 360 |
|  |
| Query 361 IILILMPISGLIENKMLKL 379 |
| IILILMPISG+IE+KMLKL |
| Sbjct 361 IILILMPISGIIEDKMLKL 379 |
|  |
| **Query #23: CAB09427.1 cytochrome b, partial [Acomys cahirinus] Query ID: lcl\|Query_8026 Length: 380** |
| **Sequence ID: P00158.1 Length: 381** |
|  |
| Query 1 MKIMRKTHPLLKIINHAFVDLPAPSNISSWWNFGSLLGVCLMIQILTGLFLAMHYTSDTM 60 |
| M MRKTHPL KIINH+F+DLPAPSNISSWWNFGSLLGVCLM+QI+TGLFLAMHYTSDTM |
| Sbjct 1 MTNMRKTHPLFKIINHSFIDLPAPSNISSWWNFGSLLGVCLMVQIITGLFLAMHYTSDTM 60 |
|  |
| Query 61 TAFSSVTHICRDVNYGWLIRYLHANGASMFFMCLFMHVGRGIYYGSYTFMETWNLGIILL 120 |
| TAFSSVTHICRDVNYGWLIRY+HANGASMFF+CLF+HVGRG+YYGSYTFMETWN+G++LL |
| Sbjct 61 TAFSSVTHICRDVNYGWLIRYMHANGASMFFICLFLHVGRGLYYGSYTFMETWNIGVLLL 120 |
|  |
| Query 121 FAVMATAFMGYVLPWGQMSFWGATVITNLLSAIPYIGTNLVEWIWGGFSVDKATLTRFFA 180 |
| FAVMATAFMGYVLPWGQMSFWGATVITNLLSAIPYIGT LVEWIWGGFSVDKATLTRFFA |
| Sbjct 121 FAVMATAFMGYVLPWGQMSFWGATVITNLLSAIPYIGTTLVEWIWGGFSVDKATLTRFFA 180 |
|  |
| Query 181 FHFILPFIIAALAMVHLLFLHETGSNNPTGMNSDADKIPFHPYYTIKDLLGVFLMLSVLL 240 |
| FHFILPFIIAALA+VHLLFLHETGSNNPTG+NSDADKIPFHPYYTIKD+LG+ +M +L+ |
| Sbjct 181 FHFILPFIIAALAIVHLLFLHETGSNNPTGLNSDADKIPFHPYYTIKDILGILIMFLILM 240 |
|  |
| Query 241 LLVLFSPDLLGDPDNYTPANPLNTPPHIKPEWYFLFAYAILRSIPNKLGGVLALVLSILI 300 |
| LVLF PD+LGDPDNY PANPLNTPPHIKPEWYFLFAYAILRSIPNKLGGVLAL+LSILI |
| Sbjct 241 TLVLFFPDMLGDPDNYMPANPLNTPPHIKPEWYFLFAYAILRSIPNKLGGVLALILSILI 300 |
|  |
| Query 301 LAFMPLTYTLKQRILMFRPISQTMFWILAANLLILTWIGGQPVEHPFIIIGQLASISYFS 360 |
| LA MP +T KQR LMFRPI+Q ++WIL ANLLILTWIGGQPVEHPFIIIGQLASISYFS |
| Sbjct 301 LALMPFLHTSKQRSLMFRPITQILYWILVANLLILTWIGGQPVEHPFIIIGQLASISYFS 360 |
|  |
| Query 361 IILILMPISGLIENKMLKL 379 |
| IILILMPISG+IE+KMLKL |
| Sbjct 361 IILILMPISGIIEDKMLKL 379 |
|  |
| **Query #24: CAA65724.1 cytochrome b, partial [Acomys cahirinus] Query ID: lcl\|Query_8027 Length: 380** |
| **Sequence ID: P00158.1 Length: 381** |
|  |
| Query 1 MKIMRKTHPLLKIINHAFVDLPAPSNISSWWNFGSLLGVCLVIQILTGLFLAMHYTSDTM 60 |
| M MRKTHPL KIINH+F+DLPAPSNISSWWNFGSLLGVCL++QI+TGLFLAMHYTSDTM |
| Sbjct 1 MTNMRKTHPLFKIINHSFIDLPAPSNISSWWNFGSLLGVCLMVQIITGLFLAMHYTSDTM 60 |
|  |
| Query 61 TAFSSVTHICRDVNYGWLIRYLHANGASMFFVCLFMHVGRGIYYGSYTFTETWNLGIILL 120 |
| TAFSSVTHICRDVNYGWLIRY+HANGASMFF+CLF+HVGRG+YYGSYTF ETWN+G++LL |
| Sbjct 61 TAFSSVTHICRDVNYGWLIRYMHANGASMFFICLFLHVGRGLYYGSYTFMETWNIGVLLL 120 |
|  |
| Query 121 FAVMATAFMGYVLPWGQMSFWGATVITNLLSAIPYIGTNLVEWIWGGFSVDKATLTRFFA 180 |
| FAVMATAFMGYVLPWGQMSFWGATVITNLLSAIPYIGT LVEWIWGGFSVDKATLTRFFA |
| Sbjct 121 FAVMATAFMGYVLPWGQMSFWGATVITNLLSAIPYIGTTLVEWIWGGFSVDKATLTRFFA 180 |
|  |
| Query 181 FHFILPFIIAALAMVHLLFLHETGSNNPTGMNSNADKIPFHPYYTIKDLLGVFLMLSVLL 240 |
| FHFILPFIIAALA+VHLLFLHETGSNNPTG+NS+ADKIPFHPYYTIKD+LG+ +M +L+ |
| Sbjct 181 FHFILPFIIAALAIVHLLFLHETGSNNPTGLNSDADKIPFHPYYTIKDILGILIMFLILM 240 |
|  |
| Query 241 LLVLFSPDLLGDPDNYTPANPLNTPPHIKPEWYFLFAYAILRSIPNKLGGVLALVLSILI 300 |
| LVLF PD+LGDPDNY PANPLNTPPHIKPEWYFLFAYAILRSIPNKLGGVLAL+LSILI |
| Sbjct 241 TLVLFFPDMLGDPDNYMPANPLNTPPHIKPEWYFLFAYAILRSIPNKLGGVLALILSILI 300 |
|  |
| Query 301 LAFMPLIHTSKQRSLMFRPISQTMFWILAANLLILTWIGGQPVEHPFIIIGQLASISYFS 360 |
| LA MP +HTSKQRSLMFRPI+Q ++WIL ANLLILTWIGGQPVEHPFIIIGQLASISYFS |
| Sbjct 301 LALMPFLHTSKQRSLMFRPITQILYWILVANLLILTWIGGQPVEHPFIIIGQLASISYFS 360 |
|  |
| Query 361 IILILMPISGLIENKMLKL 379 |
| IILILMPISG+IE+KMLKL |
| Sbjct 361 IILILMPISGIIEDKMLKL 379 |
|  |
| **Query #25: YP_007626810.1 NADH dehydrogenase subunit 1 [Acomys cahirinus] Query ID: lcl\|Query_8028 Length: 318** |
| **Sequence ID: P03888.3 Length: 318** |
|  |
| Query 4 INTLTLLIPILMAMAFLTLVERKMLGYMQLRKGPNIVGPYGILQPFADAMKLIIKEPLRP 63 |
| IN LTLL+PIL+AMAFLTLVERK+LGYMQLRKGPNIVGPYGILQPFADAMKL +KEP+RP |
| Sbjct 4 INILTLLVPILIAMAFLTLVERKILGYMQLRKGPNIVGPYGILQPFADAMKLFMKEPMRP 63 |
|  |
| Query 64 LSTSVVLFIIAPTLSLTLALSLWIPMPMPHPLANMNLSTLFILALSSLSVYSILWSGWAS 123 |
| L+TS+ LFIIAPTLSLTLALSLW+P+PMPHPL N+NL LFILA SSLSVYSILWSGWAS |
| Sbjct 64 LTTSMSLFIIAPTLSLTLALSLWVPLPMPHPLINLNLGILFILATSSLSVYSILWSGWAS 123 |
|  |
| Query 124 NSKYSLFGAIRAVAQTISYEVTMAIILLSVLLMNGSFSLQTLIITQEHMWLIIPTWPLAM 183 |
| NSKYSLFGA+RAVAQTISYEVTMAIILLSVLLMNGS+SLQTLI TQEHMWL++P WP+AM |
| Sbjct 124 NSKYSLFGALRAVAQTISYEVTMAIILLSVLLMNGSYSLQTLITTQEHMWLLLPAWPMAM 183 |
|  |
| Query 184 MWYISTLAETNRAPFDLTEGESELVSGFNVEYSAGPFALFFMAEYTNIILMNALSSIMFM 243 |
| MW+ISTLAETNRAPFDLTEGESELVSGFNVEY+AGPFALFFMAEYTNIILMNAL++I+F+ |
| Sbjct 184 MWFISTLAETNRAPFDLTEGESELVSGFNVEYAAGPFALFFMAEYTNIILMNALTTIIFL 243 |
|  |
| Query 244 APLYYADHPETFTTNFMLKTLMLTSLFLWVRASYPRFRYDHLMHLLWKNFLPLTLALCMW 303 |
| PLYY + PE ++TNFM++ L+L+S FLW+RASYPRFRYD LMHLLWKNFLPLTLALCMW |
| Sbjct 244 GPLYYINLPELYSTNFMMEALLLSSTFLWIRASYPRFRYDQLMHLLWKNFLPLTLALCMW 303 |
|  |
| Query 304 YVSLPIFLSSIPPY 317 |
| ++SLPIF + +PPY |
| Sbjct 304 HISLPIFTAGVPPY 317 |
|  |
| **Query #26: CAC18111.2 lecithin cholesterol acyl transferase, partial [Acomys cahirinus] Query ID: lcl\|Query_8029 Length: 268** |
| **Sequence ID: P16301.2 Length: 438** |
|  |
| Query 2 FTIWLDLNMFLPLGVNCWIDNTRVVYNRSSGSVTNAPGVQIRVPGFGKTYSVEYLDDNKL 61 |
| FTIWLD N+FLPLGV+CWIDNTR+VYN SSG V+NAPGVQIRVPGFGKT SVEY+DDNKL |
| Sbjct 82 FTIWLDFNLFLPLGVDCWIDNTRIVYNHSSGRVSNAPGVQIRVPGFGKTESVEYVDDNKL 141 |
|  |
| Query 62 A-YMHTLLQNLVNNGYVRDETVRAAPYDWRLEP-QQGEYYQKLAGLAEEMYAAYGKPVFL 119 |
| A Y+HTL+QNLVNNGYVRDETVRAAPYDWRL P QQ EYY+KLAGL EEMYAAYGKPVFL |
| Sbjct 142 AGYLHTLVQNLVNNGYVRDETVRAAPYDWRLAPHQQDEYYKKLAGLVEEMYAAYGKPVFL 201 |
|  |
| Query 120 IGHSLGCLX------------------------------------------------XXX 131 |
| IGHSLGCL |
| Sbjct 202 IGHSLGCLHVLHFLLRQPQSWKDHFIDGFISLGAPWGGSIKAMRILASGDNQGIPILSNI 261 |
|  |
| Query 132 XXREEQRITTTSPWMFPASQVWPEDHVFISTPNFNYTGQDFERFFTDLHFEEGWYMWLQS 191 |
| +EEQRITTTSPWM PA VWPEDHVFISTPNFNYT QDFERFFTDLHFEEGW+M+LQS |
| Sbjct 262 KLKEEQRITTTSPWMLPAPHVWPEDHVFISTPNFNYTVQDFERFFTDLHFEEGWHMFLQS 321 |
|  |
| Query 192 RDLLAGLPAPGVEVYCLYGVGLPTPYTYIYDHNFPYKDPVAALYEDGDDPVATRSTELCG 251 |
| RDLL LPAPGVEVYCLYGVG PTP+TYIYDHNFPYKDPVAALYEDGDD VATRSTELCG |
| Sbjct 322 RDLLERLPAPGVEVYCLYGVGRPTPHTYIYDHNFPYKDPVAALYEDGDDTVATRSTELCG 381 |
|  |
| Query 252 RWQGRQSQPVHLL 264 |
| +WQGRQSQPVHLL |
| Sbjct 382 QWQGRQSQPVHLL 394 |
|  |
| **Query #27: YP_007626821.1 NADH dehydrogenase subunit 6 [Acomys cahirinus] Query ID: lcl\|Query_8030 Length: 174** |
| **Sequence ID: P03925.1 Length: 172** |
|  |
| Query 1 MTNYMYVLSSIFLAGCLGLALKPSPIYGGLVLILSGCVGCFLVLGLGGSFLGLMVFLIYL 60 |
| M NY++VLSS+FL GCLGLALKPSPIYGGL LI+SG VGC +VLG GGSFLGLMVFLIYL |
| Sbjct 1 MNNYIFVLSSLFLVGCLGLALKPSPIYGGLGLIVSGFVGCLMVLGFGGSFLGLMVFLIYL 60 |
|  |
| Query 61 GGMMVVFGYTTAMAMEEYPETWGSSWLSVGVLVWGLVVEVILMYVISYYGDLGLGVEVYG 120 |
| GGM+VVFGYTTAMA EEYPETWGS+WL +G LV G+++EV L+ V++YY ++G+ + + G |
| Sbjct 61 GGMLVVFGYTTAMATEEYPETWGSNWLILGFLVLGVIMEVFLICVLNYYDEVGV-INLDG 119 |
|  |
| Query 121 MDSWAVYETDDLGVMGEGGVGVAAMYSCASWLMIVAGWSLFIGVFIIIEITRD 173 |
| + W +YE DD+GVM EGG+GVAAMYSCA+W+M+VAGWSLF G+FIIIEITRD |
| Sbjct 120 LGDWLMYEVDDVGVMLEGGIGVAAMYSCATWMMVVAGWSLFAGIFIIIEITRD 172 |
|  |
| **Query #28: YP_007626819.1 NADH dehydrogenase subunit 4 [Acomys cahirinus] Query ID: lcl\|Query_8031 Length: 457** |
| **Sequence ID: P03911.1 Length: 459** |
|  |
| Query 1 MLKIIMASTMLLPLTWLSKNKYMWINVTSHSFMISLTSLTLLWQ-NENCTTFSISLFSDH 59 |
| MLKII+ S MLLPLTWLS K W NVTS+SF+ISLTSLTLLWQ +EN FS SD |
| Sbjct 1 MLKIILPSLMLLPLTWLSSPKKTWTNVTSYSFLISLTSLTLLWQTDENYKNFSNMFSSDP 60 |
|  |
| Query 60 ISTPLIILTTWLLPLMLLASQNHMKKENPTHKKLYITMLILLQILLILTFSATEMIMFYI 119 |
| +STPLIILT WLLPLML+ASQNH+KK+N +KLYI+MLI LQILLI+TFSATE+IMFYI |
| Sbjct 61 LSTPLIILTAWLLPLMLMASQNHLKKDNNVLQKLYISMLISLQILLIMTFSATELIMFYI 120 |
|  |
| Query 120 MFEATLIPTLIIITRWGNQTERLNAGLYFLFYTLVGSIPLLIALIMTQNSTGTLNFMLMT 179 |
| +FEATLIPTLIIITRWGNQTERLNAG+YFLFYTL+GSIPLLIALI+ QN GTLN M+++ |
| Sbjct 121 LFEATLIPTLIIITRWGNQTERLNAGIYFLFYTLIGSIPLLIALILIQNHVGTLNLMILS 180 |
|  |
| Query 180 LNSQTMNTTWSNNILWLTCMLAFLIKMPLYGVHLWLPKAHVEAPIAGSMVLAAILLKLGG 239 |
| + T++ +WSNN+LWL CM+AFLIKMPLYGVHLWLPKAHVEAPIAGSM+LAAILLKLG |
| Sbjct 181 FTTHTLDASWSNNLLWLACMMAFLIKMPLYGVHLWLPKAHVEAPIAGSMILAAILLKLGS 240 |
|  |
| Query 240 YGMMRISIMLDPMTKSMSYPFIILSLWGMIMTSSICLRQTDLKSLIAYSSVSHMALVIAS 299 |
| YGM+RISI+LDP+TK M+YPFI+LSLWGMIMTSSICLRQTDLKSLIAYSSVSHMALVIAS |
| Sbjct 241 YGMIRISIILDPLTKYMAYPFILLSLWGMIMTSSICLRQTDLKSLIAYSSVSHMALVIAS 300 |
|  |
| Query 300 IMIQTPWSFMGAMALMIAHGLTSSLMFCLANSNYERTHSRTMILARSLQTIFPLMAIWWV 359 |
| IMIQTPWSFMGA LMIAHGLTSSL+FCLANSNYER HSRTMI+AR LQ +FPLMA WW+ |
| Sbjct 301 IMIQTPWSFMGATMLMIAHGLTSSLLFCLANSNYERIHSRTMIMARGLQMVFPLMATWWL 360 |
|  |
| Query 360 LASLANLALPPSINLIGELLITMSLFSWSNPSIILLGVNIIITALYSLYMIIMTQRGKLN 419 |
| +ASLANLALPPSINL+GEL ITMSLFSWSN +IIL+G+NIIIT +YS+YMII TQRGKL |
| Sbjct 361 MASLANLALPPSINLMGELFITMSLFSWSNFTIILMGINIIITGMYSMYMIITTQRGKLT 420 |
|  |
| Query 420 YHINNLHPSHTRELTLMSLHIMPLILLTVKPELILG 455 |
| H+ NL PSHTRELTLM+LH++PLILLT P+LI G |
| Sbjct 421 NHMINLQPSHTRELTLMALHMIPLILLTTSPKLITG 456 |
|  |
| **Query #29: YP_007626814.1 ATP synthase F0 subunit 8 [Acomys cahirinus] Query ID: lcl\|Query_8032 Length: 67** |
| **Sequence ID: P03930.1 Length: 67** |
|  |
| Query 1 MPQLDTSTWFITALSSTATLFILLQLKLSNIYYFNAPTPKTLKSLKTENPWEMKWTKIYS 60 |
| MPQLDTSTWFIT +SS TLFIL QLK+S+ + AP+PK+L ++K + PWE+KWTKIY |
| Sbjct 1 MPQLDTSTWFITIISSMITLFILFQLKVSSQTFPLAPSPKSLTTMKVKTPWELKWTKIYL 60 |
|  |
| Query 61 PHSLP 65 |
| PHSLP |
| Sbjct 61 PHSLP 65 |
|  |
| **Query #30: YP_007626820.1 NADH dehydrogenase subunit 5 [Acomys cahirinus] Query ID: lcl\|Query_8033 Length: 610** |
| **Sequence ID: P03921.3 Length: 607** |
|  |
| Query 23 LTHLMKHTKFPHYATLSIKNAFLISLVPLATLLHYNIESMISSWHWMTINTINLSISFKI 82 |
| +++L+KH FP Y T SIK +F+ISL+PL H N+E MI++WHW+T+N++ L +SFK |
| Sbjct 23 MSNLIKHINFPLYTTTSIKFSFIISLLPLLMFFHNNMEYMITTWHWVTMNSMELKMSFKT 82 |
|  |
| Query 83 DFFSILFLSVALFVTWSIMEFSSWYMHSDPDLNRFIKYLLLFLITMIILTSANNLFQLFI 142 |
| DFFSILF SVALFVTWSIM+FSSWYMHSDP++NRFIKYL LFLITM+ILTSANN+FQLFI |
| Sbjct 83 DFFSILFTSVALFVTWSIMQFSSWYMHSDPNINRFIKYLTLFLITMLILTSANNMFQLFI 142 |
|  |
| Query 143 GWEGVGIMSFLLIGWWYGRTEANTAALQAILYNRIGDIGLILAMTWFCLNYNSWEMQQIL 202 |
| GWEGVGIMSFLLIGWWYGRT+ANTAALQAILYNRIGDIG ILAM WF LN NSWE+QQI+ |
| Sbjct 143 GWEGVGIMSFLLIGWWYGRTDANTAALQAILYNRIGDIGFILAMVWFSLNMNSWELQQIM 202 |
|  |
| Query 203 MHNNN-NTIPLMGLLLAATGKSAQFGLHPWLPSAMEGPTPVSALLHSSTMVVAGVFLMIR 261 |
| NNN N IPLMGLL+AATGKSAQFGLHPWLPSAMEGPTPVSALLHSSTMVVAG+FL++R |
| Sbjct 203 FSNNNDNLIPLMGLLIAATGKSAQFGLHPWLPSAMEGPTPVSALLHSSTMVVAGIFLLVR 262 |
|  |
| Query 262 FFPLTSNNSTILTLMLCLGSITTLFTAICALTQNDIKKIVAFSTSSQLGLMMVTLGINQP 321 |
| F PLT+NN+ ILT MLCLG++TTLFTAICALTQNDIKKI+AFSTSSQLGLMMVTLG+NQP |
| Sbjct 263 FHPLTTNNNFILTTMLCLGALTTLFTAICALTQNDIKKIIAFSTSSQLGLMMVTLGMNQP 322 |
|  |
| Query 322 YLAFLHICMHAFFKAMLFMCSGSIIHNLNDEQDIRKMGSTTQTLPFTTSCLTIGSLALTG 381 |
| +LAFLHIC HAFFKAMLFMCSGSIIH+L DEQDIRKMG+ T+ +PFT+SCL IGSLALTG |
| Sbjct 323 HLAFLHICTHAFFKAMLFMCSGSIIHSLADEQDIRKMGNITKIMPFTSSCLVIGSLALTG 382 |
|  |
| Query 382 MPFLTGFYSKDLIIEAVNTSNTNAWALLITLIATSLTAAYSMRIIYFVTMTKPRYPPMMS 441 |
| MPFLTGFYSKDLIIEA+NT NTNAWALLITLIATS+TA YSMRIIYFVTMTKPR+PP++S |
| Sbjct 383 MPFLTGFYSKDLIIEAINTCNTNAWALLITLIATSMTAMYSMRIIYFVTMTKPRFPPLIS 442 |
|  |
| Query 442 MSENNPKLINPIKRLALGSIFAGYIISHNIPPINTQTMTMPWQLKMTALFITITGLLVAL 501 |
| ++EN+P L+NPIKRLA GSIFAG++IS+NIPP + +TMPW LK TAL I++ G L+AL |
| Sbjct 443 INENDPDLMNPIKRLAFGSIFAGFVISYNIPPTSIPVLTMPWFLKTTALIISVLGFLIAL 502 |
|  |
| Query 502 ELNNLTSNLTPNKTSPYSTFSALLGYFPTITHRLIPAKTLNLGMKISLNTLDQTWLETAI 561 |
| ELNNLT L+ NK +PYS+FS LLG+FP+I HR+ P K+LNL +K SL LD WLE I |
| Sbjct 503 ELNNLTMKLSMNKANPYSSFSTLLGFFPSIIHRITPMKSLNLSLKTSLTLLDLIWLEKTI 562 |
|  |
| Query 562 PKSTSTLHTMMSKHISNQKGLIKIYFLSF 590 |
| PKSTSTLHT M+ +NQKGLIK+YF+SF |
| Sbjct 563 PKSTSTLHTNMTTLTTNQKGLIKLYFMSF 591 |
|  |
| **Query #31: YP_007626813.1 cytochrome c oxidase subunit II [Acomys cahirinus] Query ID: lcl\|Query_8034 Length: 227** |
| **Sequence ID: P00405.1 Length: 227** |
|  |
| Query 1 MAYPFQLGLQDATSPIMEELTSFHDHTLMIVFLISSLVLYIISSMLSTKMTHTSTMDAQG 60 |
| MAYPFQLGLQDATSPIMEEL +FHDHTLMIVFLISSLVLYIIS ML+TK+THTSTMDAQ |
| Sbjct 1 MAYPFQLGLQDATSPIMEELMNFHDHTLMIVFLISSLVLYIISLMLTTKLTHTSTMDAQE 60 |
|  |
| Query 61 VETIWTILPAAILVLIALPSLRILYMMDEINNPVLTVKTMGHQWYWSYEYTDYEDLCFDS 120 |
| VETIWTILPA IL++IALPSLRILYMMDEINNPVLTVKTMGHQWYWSYEYTDYEDLCFDS |
| Sbjct 61 VETIWTILPAVILIMIALPSLRILYMMDEINNPVLTVKTMGHQWYWSYEYTDYEDLCFDS 120 |
|  |
| Query 121 YMTPTSDLKPGELRLLEVDNRVVLPMELPIRMLISSEDVLHAWAVPSLGLKTDAIPGRLN 180 |
| YM PT+DLKPGELRLLEVDNRVVLPMELPIRMLISSEDVLH+WAVPSLGLKTDAIPGRLN |
| Sbjct 121 YMIPTNDLKPGELRLLEVDNRVVLPMELPIRMLISSEDVLHSWAVPSLGLKTDAIPGRLN 180 |
|  |
| Query 181 QATISSNRPGLFYGQCSEICGSNHSFMPIVLEMVPLKHFENWSASMI 227 |
| QAT++SNRPGLFYGQCSEICGSNHSFMPIVLEMVPLK+FENWSASMI |
| Sbjct 181 QATVTSNRPGLFYGQCSEICGSNHSFMPIVLEMVPLKYFENWSASMI 227 |
|  |
| **Query #32: YP_007626815.1 ATP synthase F0 subunit 6 [Acomys cahirinus] Query ID: lcl\|Query_8035 Length: 226** |
| **Sequence ID: P00848.1 Length: 226** |
|  |
| Query 1 MNENLFSSFITPTLMGLPVVIIIITLPFIMFPNSKRLFNNRLISLQQWLIKLIMKQMMLI 60 |
| MNENLF+SFITPT+MG P+V+ II P I+FP+SKRL NNRL S Q WL+KLI+KQMMLI |
| Sbjct 1 MNENLFASFITPTMMGFPIVVAIIMFPSILFPSSKRLINNRLHSFQHWLVKLIIKQMMLI 60 |
|  |
| Query 61 HTPKGRTWSLMIISLIMFIGSTNLLGLLPHTFTPTTQLSMNLSMAIPLWAGAVIMGFRHK 120 |
| HTPKGRTW+LMI+SLIMFIGSTNLLGLLPHTFTPTTQLSMNLSMAIPLWAGAVI GFRHK |
| Sbjct 61 HTPKGRTWTLMIVSLIMFIGSTNLLGLLPHTFTPTTQLSMNLSMAIPLWAGAVITGFRHK 120 |
|  |
| Query 121 LKDSLAHFLPQGTPITLIPMLIIIETISLFIQPMALAVRLTANITAGHLLMHLIGGATLV 180 |
| LK SLAHFLPQGTPI+LIPMLIIIETISLFIQPMALAVRLTANITAGHLLMHLIGGATLV |
| Sbjct 121 LKSSLAHFLPQGTPISLIPMLIIIETISLFIQPMALAVRLTANITAGHLLMHLIGGATLV 180 |
|  |
| Query 181 LMSISPPTALITFIILLLLTVLEFAVALIQAYVFTLLVSLYLHDNT 226 |
| LM+ISPPTA ITFIILLLLT+LEFAVALIQAYVFTLLVSLYLHDNT |
| Sbjct 181 LMNISPPTATITFIILLLLTILEFAVALIQAYVFTLLVSLYLHDNT 226 |
|  |
| **Query #33: YP_007626812.1 cytochrome c oxidase subunit I [Acomys cahirinus] Query ID: lcl\|Query_8036 Length: 514** |
| **Sequence ID: P00397.2 Length: 514** |
|  |
| Query 1 MFINRWLFSTNHKDIGTLYLIFGAWAGMVGTALSILIRAELGQPGALLGDDQIYNVIVTA 60 |
| MFINRWLFSTNHKDIGTLYL+FGAWAGMVGTALSILIRAELGQPGALLGDDQIYNVIVTA |
| Sbjct 1 MFINRWLFSTNHKDIGTLYLLFGAWAGMVGTALSILIRAELGQPGALLGDDQIYNVIVTA 60 |
|  |
| Query 61 HAFVMIFFMVMPMMIGGFGNWLVPLMIGAPDMAFPRMNNMSFWLLPPSFLLLMASSMVEA 120 |
| HAFVMIFFMVMPMMIGGFGNWLVPLMIGAPDMAFPRMNNMSFWLLPPSFLLL+ASSMVEA |
| Sbjct 61 HAFVMIFFMVMPMMIGGFGNWLVPLMIGAPDMAFPRMNNMSFWLLPPSFLLLLASSMVEA 120 |
|  |
| Query 121 GAGTGWTVYPPLAGNLAHAGASVDLAIFSLHLAGVSSILGAINFITTIINMKPPAITQYQ 180 |
| GAGTGWTVYPPLAGNLAHAGASVDL IFSLHLAGVSSILGAINFITTIINMKPPA+TQYQ |
| Sbjct 121 GAGTGWTVYPPLAGNLAHAGASVDLTIFSLHLAGVSSILGAINFITTIINMKPPAMTQYQ 180 |
|  |
| Query 181 TPLFVWSVLITAVLLLLSLPVLAAGITMLLTDRNLNTTFFDPAGGGDPILYQHLFWFFGH 240 |
| TPLFVWSVLITAVLLLLSLPVLAAGITMLLTDRNLNTTFFDPAGGGDPILYQHLFWFFGH |
| Sbjct 181 TPLFVWSVLITAVLLLLSLPVLAAGITMLLTDRNLNTTFFDPAGGGDPILYQHLFWFFGH 240 |
|  |
| Query 241 PEVYILILPGFGIISHIVTYYSGKKEPFGYMGMVWAMMSIGFLGFIVWAHHMFTVGLDVD 300 |
| PEVYILILPGFGIISH+VTYYSGKKEPFGYMGMVWAMMSIGFLGFIVWAHHMFTVGLDVD |
| Sbjct 241 PEVYILILPGFGIISHVVTYYSGKKEPFGYMGMVWAMMSIGFLGFIVWAHHMFTVGLDVD 300 |
|  |
| Query 301 TRAYFTSATMIIAIPTGVKVFSWLATLHGGNIKWSPAMLWALGFIFLFTVGGLTGIVLSN 360 |
| TRAYFTSATMIIAIPTGVKVFSWLATLHGGNIKWSPAMLWALGFIFLFTVGGLTGIVLSN |
| Sbjct 301 TRAYFTSATMIIAIPTGVKVFSWLATLHGGNIKWSPAMLWALGFIFLFTVGGLTGIVLSN 360 |
|  |
| Query 361 SSLDIVLHDTYYVVAHFHYVLSMGAVFAIMAGFVHWFPLFSGYSLNDMWAKAHFIVMFVG 420 |
| SSLDIVLHDTYYVVAHFHYVLSMGAVFAIMAGFVHWFPLFSG++L+D WAKAHF +MFVG |
| Sbjct 361 SSLDIVLHDTYYVVAHFHYVLSMGAVFAIMAGFVHWFPLFSGFTLDDTWAKAHFAIMFVG 420 |
|  |
| Query 421 VNLTFFPQHFLGLSGMPRRYSDYPDAYTTWNMVSSMGSFISLTAVIIMIFIIWEAFASKR 480 |
| VN+TFFPQHFLGLSGMPRRYSDYPDAYTTWN VSSMGSFISLTAV+IMIF+IWEAFASKR |
| Sbjct 421 VNMTFFPQHFLGLSGMPRRYSDYPDAYTTWNTVSSMGSFISLTAVLIMIFMIWEAFASKR 480 |
|  |
| Query 481 EILSVPYTATNLEWLHGCPPPYHTFEEPTYVKVK 514 |
| E++SV Y +TNLEWLHGCPPPYHTFEEPTYVKVK |
| Sbjct 481 EVMSVSYASTNLEWLHGCPPPYHTFEEPTYVKVK 514 |
|  |
| **Query #34: YP_007626816.1 cytochrome c oxidase subunit III [Acomys cahirinus] Query ID: lcl\|Query_8037 Length: 261** |
| **Sequence ID: P00416.2 Length: 261** |
|  |
| Query 1 MNHQTHAFHMVNPSPWPLTGALSALLLTSGLIMWFHYNSTILLTTGLLTNLLTMYQWWRD 60 |
| M HQTHA+HMVNPSPWPLTGA SALLLTSGL+MWFHYNS LLT GLLTN+LTMYQWWRD |
| Sbjct 1 MTHQTHAYHMVNPSPWPLTGAFSALLLTSGLVMWFHYNSITLLTLGLLTNILTMYQWWRD 60 |
|  |
| Query 61 VIREGTFQGHHTPIVQKGLRYGMILFIVSEIFFFAGFFWAFYHSSLVPTHDLGGFWPPAG 120 |
| VIREGT+QGHHTPIVQKGLRYGMILFIVSE+FFFAGFFWAFYHSSLVPTHDLGG WPP G |
| Sbjct 61 VIREGTYQGHHTPIVQKGLRYGMILFIVSEVFFFAGFFWAFYHSSLVPTHDLGGCWPPTG 120 |
|  |
| Query 121 ISPLNPLEVPLLNTSVLLASGVSITWAHHSLMEGKRMHMNQALSITIALGLYFTILQASE 180 |
| ISPLNPLEVPLLNTSVLLASGVSITWAHHSLMEGKR HMNQAL ITI LGLYFTILQASE |
| Sbjct 121 ISPLNPLEVPLLNTSVLLASGVSITWAHHSLMEGKRNHMNQALLITIMLGLYFTILQASE 180 |
|  |
| Query 181 YFETSFSISDGIYGSTFFMATGFHGLHVIIGTTFLMICLLRQMKFHFTSKHHFGFEAAAW 240 |
| YFETSFSISDGIYGSTFFMATGFHGLHVIIG+TFL++CLLRQ+KFHFTSKHHFGFEAAAW |
| Sbjct 181 YFETSFSISDGIYGSTFFMATGFHGLHVIIGSTFLIVCLLRQLKFHFTSKHHFGFEAAAW 240 |
|  |
| Query 241 YWHFVDVVWLFLYVSIYWWGS 261 |
| YWHFVDVVWLFLYVSIYWWGS |
| Sbjct 241 YWHFVDVVWLFLYVSIYWWGS 261 |
|  |
| **Query #35: YP_007626811.1 NADH dehydrogenase subunit 2 [Acomys cahirinus] Query ID: lcl\|Query_8038 Length: 345** |
| **Sequence ID: P03893.2 Length: 345** |
|  |
| Query 1 MNPLTLLTVSLTILMGPIVTMMSSNLLLMWVGLEMSLLAMIPLLTHKKSPRSTEAATKYF 60 |
| MNP+TL + TI +GP++TM S+NL+LMWVGLE SLLA+IP+L +KK+PRSTEAATKYF |
| Sbjct 1 MNPITLAIIYFTIFLGPVITMSSTNLMLMWVGLEFSLLAIIPMLINKKNPRSTEAATKYF 60 |
|  |
| Query 61 MTQATASMIMLLAIILNHIQLGSWNMHPQTNKLTLTIMFIALVIKLGLAPFHTWLPEVTQ 120 |
| +TQATASMI+LLAI+LN+ QLG+W QTN L L + +AL +KLGLAPFH WLPEVTQ |
| Sbjct 61 VTQATASMIILLAIVLNYKQLGTWMFQQQTNGLILNMTLMALSMKLGLAPFHFWLPEVTQ 120 |
|  |
| Query 121 GVPLSSGLILLTWQKIAPLSILYQIHELLDPLLTTTISIISVFTGAWGGLNQTQMRKIMA 180 |
| G+PL GLILLTWQKIAPLSIL QI+ LL+ + ++I S+F GAWGGLNQTQMRKIMA |
| Sbjct 121 GIPLHMGLILLTWQKIAPLSILIQIYPLLNSTIILMLAITSIFMGAWGGLNQTQMRKIMA 180 |
|  |
| Query 181 YSSIAHMGWMLAILPYNPSLTLLNLTIYILMTISMFLMLMTSSYTTINSISMAWNKHPIM 240 |
| YSSIAHMGWMLAILPYNPSLTLLNL IYI++T MF+ LM ++ TINSIS+ WNK P M |
| Sbjct 181 YSSIAHMGWMLAILPYNPSLTLLNLMIYIILTAPMFMALMLNNSMTINSISLLWNKTPAM 240 |
|  |
| Query 241 IPLMATVLLSLGGLPPLSGFMPKWLIITELLKNNSTPAASLIALMALLNLFFYTRLIYST 300 |
| + +++ +LLSLGGLPPL+GF+PKW+IITEL+KNN A+L+A+MALLNLFFYTRLIYST |
| Sbjct 241 LTMISLMLLSLGGLPPLTGFLPKWIIITELMKNNCLIMATLMAMMALLNLFFYTRLIYST 300 |
|  |
| Query 301 SLTIFPTNNNSKLIPQQLASKHFPMMPLLMTLSTMILPLTPLLI 344 |
| SLT+FPTNNNSK++ Q +K M L +STM LPL P LI |
| Sbjct 301 SLTMFPTNNNSKMMTHQTKTKPNLMFSTLAIMSTMTLPLAPQLI 344 |
|  |
| **Query #36: AEP83312.1 cytochrome b [Acomys cahirinus] Query ID: lcl\|Query_8039 Length: 380** |
| **Sequence ID: P00158.1 Length: 381** |
|  |
| Query 1 MKITRKTHPLLKIINHAFVDLPAPSNISSWWNFGSLLGVCLVMQILTGLFLAMHYTSDTM 60 |
| M RKTHPL KIINH+F+DLPAPSNISSWWNFGSLLGVCL++QI+TGLFLAMHYTSDTM |
| Sbjct 1 MTNMRKTHPLFKIINHSFIDLPAPSNISSWWNFGSLLGVCLMVQIITGLFLAMHYTSDTM 60 |
|  |
| Query 61 TAFSSVTHICRDVNYGWLIRYLHANGASMFFVCLFMHVGRGIYYGSYTFTETWNLGVILL 120 |
| TAFSSVTHICRDVNYGWLIRY+HANGASMFF+CLF+HVGRG+YYGSYTF ETWN+GV+LL |
| Sbjct 61 TAFSSVTHICRDVNYGWLIRYMHANGASMFFICLFLHVGRGLYYGSYTFMETWNIGVLLL 120 |
|  |
| Query 121 FAVMATAFMGYVLPWGQMSFWGATVITNLLSAIPYIGTNLVEWIWGGFSVDKATLTRFFA 180 |
| FAVMATAFMGYVLPWGQMSFWGATVITNLLSAIPYIGT LVEWIWGGFSVDKATLTRFFA |
| Sbjct 121 FAVMATAFMGYVLPWGQMSFWGATVITNLLSAIPYIGTTLVEWIWGGFSVDKATLTRFFA 180 |
|  |
| Query 181 FHFILPFIIAALAMVHLLFLHETGSNNPTGVNSDADKIPFHPYYTIKDLLGVFLMLLILL 240 |
| FHFILPFIIAALA+VHLLFLHETGSNNPTG+NSDADKIPFHPYYTIKD+LG+ +M LIL+ |
| Sbjct 181 FHFILPFIIAALAIVHLLFLHETGSNNPTGLNSDADKIPFHPYYTIKDILGILIMFLILM 240 |
|  |
| Query 241 LLVLFSPDLLGDPDNYTPANPLNTPPHIKPEWYFLFAYAILRSIPNKLGGVLALVLSILI 300 |
| LVLF PD+LGDPDNY PANPLNTPPHIKPEWYFLFAYAILRSIPNKLGGVLAL+LSILI |
| Sbjct 241 TLVLFFPDMLGDPDNYMPANPLNTPPHIKPEWYFLFAYAILRSIPNKLGGVLALILSILI 300 |
|  |
| Query 301 LILMPLIHTSKQRSLMFRPISQAMFWILTANLLILTWIGGQPVEHPFIIIGQLASISYFS 360 |
| L LMP +HTSKQRSLMFRPI+Q ++WIL ANLLILTWIGGQPVEHPFIIIGQLASISYFS |
| Sbjct 301 LALMPFLHTSKQRSLMFRPITQILYWILVANLLILTWIGGQPVEHPFIIIGQLASISYFS 360 |
|  |
| Query 361 IILILMPISGLIENKMLKL 379 |
| IILILMPISG+IE+KMLKL |
| Sbjct 361 IILILMPISGIIEDKMLKL 379 |
|  |
| **Query #37: AEP83361.1 NADH dehydrogenase subunit 4 [Acomys cahirinus] Query ID: lcl\|Query_8040 Length: 457** |
| **Sequence ID: P03911.1 Length: 459** |
|  |
| Query 1 MLKIIMASTMLLPLTWLSKNKYMWINVTSHSFMISLTSLTLLWQ-NENCTTFSISLFSDH 59 |
| MLKII+ S MLLPLTWLS K W NVTS+SF+ISLTSLTLLWQ +EN FS SD |
| Sbjct 1 MLKIILPSLMLLPLTWLSSPKKTWTNVTSYSFLISLTSLTLLWQTDENYKNFSNMFSSDP 60 |
|  |
| Query 60 ISTPLIILTTWLLPLMLLASQNHMKKENPTHKKLYITMLILLQILLILTFSATEMIMFYI 119 |
| +STPLIILT WLLPLML+ASQNH+KK+N +KLYI+MLI LQILLI+TFSATE+IMFYI |
| Sbjct 61 LSTPLIILTAWLLPLMLMASQNHLKKDNNVLQKLYISMLISLQILLIMTFSATELIMFYI 120 |
|  |
| Query 120 MFEATLIPTLIIITRWGNQTERLNAGLYFLFYTLVGSIPLLIALIMTQNSTGTLNFMLMT 179 |
| +FEATLIPTLIIITRWGNQTERLNAG+YFLFYTL+GSIPLLIALI+ QN GTLN M+++ |
| Sbjct 121 LFEATLIPTLIIITRWGNQTERLNAGIYFLFYTLIGSIPLLIALILIQNHVGTLNLMILS 180 |
|  |
| Query 180 LNSQTMNTTWSNNILWLTCMLAFLIKMPLYGVHLWLPKAHVEAPIAGSMVLAAILLKLGG 239 |
| + T++ +WSNN+LWL CM+AFLIKMPLYGVHLWLPKAHVEAPIAGSM+LAAILLKLG |
| Sbjct 181 FTTHTLDASWSNNLLWLACMMAFLIKMPLYGVHLWLPKAHVEAPIAGSMILAAILLKLGS 240 |
|  |
| Query 240 YGMMRISIMLDPMTKSMSYPFIILSLWGMIMTSSICLRQTDLKSLIAYSSVSHMALVIAS 299 |
| YGM+RISI+LDP+TK M+YPFI+LSLWGMIMTSSICLRQTDLKSLIAYSSVSHMALVIAS |
| Sbjct 241 YGMIRISIILDPLTKYMAYPFILLSLWGMIMTSSICLRQTDLKSLIAYSSVSHMALVIAS 300 |
|  |
| Query 300 IMIQTPWSFMGGMALMIAHGLTSSLMFCLANSNYERTHSRTMILARSLQTIFPLMAIWWV 359 |
| IMIQTPWSFMG LMIAHGLTSSL+FCLANSNYER HSRTMI+AR LQ +FPLMA WW+ |
| Sbjct 301 IMIQTPWSFMGATMLMIAHGLTSSLLFCLANSNYERIHSRTMIMARGLQMVFPLMATWWL 360 |
|  |
| Query 360 LASLANLALPPSINLIGELLITMSLFSWSNPSIILLGVNIIITALYSLYMIIMTQRGKLN 419 |
| +ASLANLALPPSINL+GEL ITMSLFSWSN +IIL+G+NIIIT +YS+YMII TQRGKL |
| Sbjct 361 MASLANLALPPSINLMGELFITMSLFSWSNFTIILMGINIIITGMYSMYMIITTQRGKLT 420 |
|  |
| Query 420 YHINNLHPSHTRELTLMSLHIMPLILLTVKPELILG 455 |
| H+ NL PSHTRELTLM+LH++PLILLT P+LI G |
| Sbjct 421 NHMINLQPSHTRELTLMALHMIPLILLTTSPKLITG 456 |
|  |
| **Query #38: AEP83283.1 NADH dehydrogenase subunit 4 [Acomys cahirinus] Query ID: lcl\|Query_8041 Length: 457** |
| **Sequence ID: P03911.1 Length: 459** |
|  |
| Query 1 MLKIIMASTMLLPLTWLSKNKYMWINVTSHSFMISLTSLTLLWQ-NENCTTFSISLFSDH 59 |
| MLKII+ S MLLPLTWLS K W NVTS+SF+ISLTSLTLLWQ +EN FS SD |
| Sbjct 1 MLKIILPSLMLLPLTWLSSPKKTWTNVTSYSFLISLTSLTLLWQTDENYKNFSNMFSSDP 60 |
|  |
| Query 60 ISTPLIILTTWLLPLMLLASQNHMKKENPTHKKLYITMLILLQILLILTFSATEMIMFYI 119 |
| +STPLIILT WLLPLML+ASQNH+KK+N +KLYI+MLI LQILLI+TFSATE+IMFYI |
| Sbjct 61 LSTPLIILTAWLLPLMLMASQNHLKKDNNVLQKLYISMLISLQILLIMTFSATELIMFYI 120 |
|  |
| Query 120 MFEATLIPTLIIITRWGNQTERLNAGLYFLFYTLAGSIPLLIALIMTQNSTGTLNFMLMT 179 |
| +FEATLIPTLIIITRWGNQTERLNAG+YFLFYTL GSIPLLIALI+ QN GTLN M+++ |
| Sbjct 121 LFEATLIPTLIIITRWGNQTERLNAGIYFLFYTLIGSIPLLIALILIQNHVGTLNLMILS 180 |
|  |
| Query 180 LNSQTMNTTWSNNILWLTCMLAFLIKMPLYGVHLWLPKAHVEAPIAGSMVLAAILLKLGG 239 |
| + T++ +WSNN+LWL CM+AFLIKMPLYGVHLWLPKAHVEAPIAGSM+LAAILLKLG |
| Sbjct 181 FTTHTLDASWSNNLLWLACMMAFLIKMPLYGVHLWLPKAHVEAPIAGSMILAAILLKLGS 240 |
|  |
| Query 240 YGMMRISIMLDPMTKSMSYPFIILSLWGMIMTSSICLRQTDLKSLIAYSSVSHMALVIAS 299 |
| YGM+RISI+LDP+TK M+YPFI+LSLWGMIMTSSICLRQTDLKSLIAYSSVSHMALVIAS |
| Sbjct 241 YGMIRISIILDPLTKYMAYPFILLSLWGMIMTSSICLRQTDLKSLIAYSSVSHMALVIAS 300 |
|  |
| Query 300 IMIQTPWSFMGAMALMIAHGLTSSLMFCLANSNYERTHSRTMILARSLQTIFPLMAIWWV 359 |
| IMIQTPWSFMGA LMIAHGLTSSL+FCLANSNYER HSRTMI+AR LQ +FPLMA WW+ |
| Sbjct 301 IMIQTPWSFMGATMLMIAHGLTSSLLFCLANSNYERIHSRTMIMARGLQMVFPLMATWWL 360 |
|  |
| Query 360 LASLANLALPPSINLIGELLITMSLFSWSNPSIILLGVNIIITALYSLYMIIMTQRGKLN 419 |
| +ASLANLALPPSINL+GEL ITMSLFSWSN +IIL+G+NIIIT +YS+YMII TQRGKL |
| Sbjct 361 MASLANLALPPSINLMGELFITMSLFSWSNFTIILMGINIIITGMYSMYMIITTQRGKLT 420 |
|  |
| Query 420 YHINNLHPSHTRELTLMSLHIMPLILLTVKPELILG 455 |
| H+ NL PSHTRELTLM+LH++PLILLT P+LI G |
| Sbjct 421 NHMINLQPSHTRELTLMALHMIPLILLTTSPKLITG 456 |
|  |
| **Query #39: AEP83280.1 cytochrome c oxidase subunit III [Acomys cahirinus] Query ID: lcl\|Query_8042 Length: 261** |
| **Sequence ID: P00416.2 Length: 261** |
|  |
| Query 1 MNHQTHAFHMVNPSPWPLTGALSALLLTSGLVMWFHYNSTILLTTGLLTNLLTMYQWWRD 60 |
| M HQTHA+HMVNPSPWPLTGA SALLLTSGLVMWFHYNS LLT GLLTN+LTMYQWWRD |
| Sbjct 1 MTHQTHAYHMVNPSPWPLTGAFSALLLTSGLVMWFHYNSITLLTLGLLTNILTMYQWWRD 60 |
|  |
| Query 61 VIREGTFQGHHTPIVQKGLRYGMILFIVSEIFFFAGFFWAFYHSSLVPTHDLGGFWPPAG 120 |
| VIREGT+QGHHTPIVQKGLRYGMILFIVSE+FFFAGFFWAFYHSSLVPTHDLGG WPP G |
| Sbjct 61 VIREGTYQGHHTPIVQKGLRYGMILFIVSEVFFFAGFFWAFYHSSLVPTHDLGGCWPPTG 120 |
|  |
| Query 121 ISPLNPLEVPLLNTSVLLASGVSITWAHHSLMEGKRMHMNQALSITIALGLYFTILQASE 180 |
| ISPLNPLEVPLLNTSVLLASGVSITWAHHSLMEGKR HMNQAL ITI LGLYFTILQASE |
| Sbjct 121 ISPLNPLEVPLLNTSVLLASGVSITWAHHSLMEGKRNHMNQALLITIMLGLYFTILQASE 180 |
|  |
| Query 181 YFETSFSISDGIYGSTFFMATGFHGLHVIIGTTFLMICLLRQMKFHFTSKHHFGFEAAAW 240 |
| YFETSFSISDGIYGSTFFMATGFHGLHVIIG+TFL++CLLRQ+KFHFTSKHHFGFEAAAW |
| Sbjct 181 YFETSFSISDGIYGSTFFMATGFHGLHVIIGSTFLIVCLLRQLKFHFTSKHHFGFEAAAW 240 |
|  |
| Query 241 YWHFVDVVWLFLYVSIYWWGS 261 |
| YWHFVDVVWLFLYVSIYWWGS |
| Sbjct 241 YWHFVDVVWLFLYVSIYWWGS 261 |
|  |
| **Query #40: AEP83265.1 ATP synthase F0 subunit 8 [Acomys cahirinus] Query ID: lcl\|Query_8043 Length: 67** |
| **Sequence ID: P03930.1 Length: 67** |
|  |
| Query 1 MPQLDTSTWFITALSSTATLFILLQLKFSNIYYFNAPTPKTLKSLKTENPWEMKWTKIYS 60 |
| MPQLDTSTWFIT +SS TLFIL QLK S+ + AP+PK+L ++K + PWE+KWTKIY |
| Sbjct 1 MPQLDTSTWFITIISSMITLFILFQLKVSSQTFPLAPSPKSLTTMKVKTPWELKWTKIYL 60 |
|  |
| Query 61 PHSLP 65 |
| PHSLP |
| Sbjct 61 PHSLP 65 |
|  |
| **Query #41: AEP83286.1 cytochrome b [Acomys cahirinus] Query ID: lcl\|Query_8044 Length: 380** |
| **Sequence ID: P00158.1 Length: 381** |
|  |
| Query 1 MKIMRKTHPLLKIINHAFVDLPTPSNISSWWNFGSLLGVCLVMQILTGLFLAMHYTSDTM 60 |
| M MRKTHPL KIINH+F+DLP PSNISSWWNFGSLLGVCL++QI+TGLFLAMHYTSDTM |
| Sbjct 1 MTNMRKTHPLFKIINHSFIDLPAPSNISSWWNFGSLLGVCLMVQIITGLFLAMHYTSDTM 60 |
|  |
| Query 61 TAFSSVTHICRDVNYGWLIRYLHANGASMFFVCLFMHVGRGIYYGSYTFTETWNLGVILL 120 |
| TAFSSVTHICRDVNYGWLIRY+HANGASMFF+CLF+HVGRG+YYGSYTF ETWN+GV+LL |
| Sbjct 61 TAFSSVTHICRDVNYGWLIRYMHANGASMFFICLFLHVGRGLYYGSYTFMETWNIGVLLL 120 |
|  |
| Query 121 FAVMATAFMGYVLPWGQMSFWGATVITNLLSAIPYIGTNLVEWIWGGFSVDKATLTRFFA 180 |
| FAVMATAFMGYVLPWGQMSFWGATVITNLLSAIPYIGT LVEWIWGGFSVDKATLTRFFA |
| Sbjct 121 FAVMATAFMGYVLPWGQMSFWGATVITNLLSAIPYIGTTLVEWIWGGFSVDKATLTRFFA 180 |
|  |
| Query 181 FHFILPFIIAALAMVHLLFLHETGSNNPTGVNSDADKIPFHPYYTIKDLLGVFLMLLILL 240 |
| FHFILPFIIAALA+VHLLFLHETGSNNPTG+NSDADKIPFHPYYTIKD+LG+ +M LIL+ |
| Sbjct 181 FHFILPFIIAALAIVHLLFLHETGSNNPTGLNSDADKIPFHPYYTIKDILGILIMFLILM 240 |
|  |
| Query 241 LLVLFSPDLLGDPDNYTPANPLNTPPHIKPEWYFLFAYAILRSIPNKLGGVLALVLSILI 300 |
| LVLF PD+LGDPDNY PANPLNTPPHIKPEWYFLFAYAILRSIPNKLGGVLAL+LSILI |
| Sbjct 241 TLVLFFPDMLGDPDNYMPANPLNTPPHIKPEWYFLFAYAILRSIPNKLGGVLALILSILI 300 |
|  |
| Query 301 LILMPLIHTSKQRSLMFRPISQAMFWILTANLLILTWIGGQPVEHPFIIIGQLASISYFS 360 |
| L LMP +HTSKQRSLMFRPI+Q ++WIL ANLLILTWIGGQPVEHPFIIIGQLASISYFS |
| Sbjct 301 LALMPFLHTSKQRSLMFRPITQILYWILVANLLILTWIGGQPVEHPFIIIGQLASISYFS 360 |
|  |
| Query 361 IILILMPISGLIENKMLKL 379 |
| IILILMPISG+IE+KMLKL |
| Sbjct 361 IILILMPISGIIEDKMLKL 379 |
|  |
| **Query #42: AEP83322.1 NADH dehydrogenase subunit 4 [Acomys cahirinus] Query ID: lcl\|Query_8045 Length: 457** |
| **Sequence ID: P03911.1 Length: 459** |
|  |
| Query 1 MLKIIMASTMLLPLTWLSKNKYMWINVTSHSFMISLTSLTLLWQ-NENCTTFSISLFSDH 59 |
| MLKII+ S MLLPLTWLS K W NVTS+SF+ISLTSLTLLWQ +EN FS SD |
| Sbjct 1 MLKIILPSLMLLPLTWLSSPKKTWTNVTSYSFLISLTSLTLLWQTDENYKNFSNMFSSDP 60 |
|  |
| Query 60 ISTPLIILTTWLLPLMLLASQNHMKKENPTHKKLYITMLILLQILLILTFSATEMIMFYI 119 |
| +STPLIILT WLLPLML+ASQNH+KK+N +KLYI+MLI LQILLI+TFSATE+IMFYI |
| Sbjct 61 LSTPLIILTAWLLPLMLMASQNHLKKDNNVLQKLYISMLISLQILLIMTFSATELIMFYI 120 |
|  |
| Query 120 MFEATLIPTLIIITRWGNQTERLNAGLYFLFYTLMGSIPLLIALIMTQNSTGTLNFMLMT 179 |
| +FEATLIPTLIIITRWGNQTERLNAG+YFLFYTL+GSIPLLIALI+ QN GTLN M+++ |
| Sbjct 121 LFEATLIPTLIIITRWGNQTERLNAGIYFLFYTLIGSIPLLIALILIQNHVGTLNLMILS 180 |
|  |
| Query 180 LNSQTMNTTWSNNILWLTCMLAFLIKMPLYGVHLWLPKAHVEAPIAGSMVLAAILLKLGG 239 |
| + T++ +WSNN+LWL CM+AFLIKMPLYGVHLWLPKAHVEAPIAGSM+LAAILLKLG |
| Sbjct 181 FTTHTLDASWSNNLLWLACMMAFLIKMPLYGVHLWLPKAHVEAPIAGSMILAAILLKLGS 240 |
|  |
| Query 240 YGMMRISIMLDPMTKSMSYPFIILSLWGMIMTSSICLRQTDLKSLIAYSSVSHMALVIAS 299 |
| YGM+RISI+LDP+TK M+YPFI+LSLWGMIMTSSICLRQTDLKSLIAYSSVSHMALVIAS |
| Sbjct 241 YGMIRISIILDPLTKYMAYPFILLSLWGMIMTSSICLRQTDLKSLIAYSSVSHMALVIAS 300 |
|  |
| Query 300 IMIQTPWSFMGAMALMIAHGLTSSLMFCLANSNYERTHSRTMILARSLQTIFPLMAIWWV 359 |
| IMIQTPWSFMGA LMIAHGLTSSL+FCLANSNYER HSRTMI+AR LQ +FPLMA WW+ |
| Sbjct 301 IMIQTPWSFMGATMLMIAHGLTSSLLFCLANSNYERIHSRTMIMARGLQMVFPLMATWWL 360 |
|  |
| Query 360 LASLANLALPPSINLIGELLITMSLFSWSNPSIILLGVNIIITALYSLYMIIMTQRGKLN 419 |
| +ASLANLALPPSINL+GEL ITMSLFSWSN +IIL+G+NIIIT +YS+YMII TQRGKL |
| Sbjct 361 MASLANLALPPSINLMGELFITMSLFSWSNFTIILMGINIIITGMYSMYMIITTQRGKLT 420 |
|  |
| Query 420 YHINNLHPSHTRELTLMSLHIMPLILLTVKPELILG 455 |
| H+ NL PSHTRELTLM+LH++PLILLT P+LI G |
| Sbjct 421 NHMINLQPSHTRELTLMALHMIPLILLTTSPKLITG 456 |
|  |
| **Query #43: AEP83392.1 NADH dehydrogenase subunit 2 [Acomys cahirinus] Query ID: lcl\|Query_8046 Length: 345** |
| **Sequence ID: P03893.2 Length: 345** |
|  |
| Query 1 MNPLTLLTVSLTILMGPIVTMMSSNLLLMWVGLEMSLLAMIPLLTHKKSPRSTEAATKYF 60 |
| MNP+TL + TI +GP++TM S+NL+LMWVGLE SLLA+IP+L +KK+PRSTEAATKYF |
| Sbjct 1 MNPITLAIIYFTIFLGPVITMSSTNLMLMWVGLEFSLLAIIPMLINKKNPRSTEAATKYF 60 |
|  |
| Query 61 MTQTTASMIMLLAIILNHIQLGSWNMHPQTNKLTLTIMFIALVIKLGLAPFHTWLPEVTQ 120 |
| +TQ TASMI+LLAI+LN+ QLG+W QTN L L + +AL +KLGLAPFH WLPEVTQ |
| Sbjct 61 VTQATASMIILLAIVLNYKQLGTWMFQQQTNGLILNMTLMALSMKLGLAPFHFWLPEVTQ 120 |
|  |
| Query 121 GVPLSSGLILLTWQKIAPLSILYQIHELLDPLLTTTISIISVFTGAWGGLNQTQMRKIMA 180 |
| G+PL GLILLTWQKIAPLSIL QI+ LL+ + ++I S+F GAWGGLNQTQMRKIMA |
| Sbjct 121 GIPLHMGLILLTWQKIAPLSILIQIYPLLNSTIILMLAITSIFMGAWGGLNQTQMRKIMA 180 |
|  |
| Query 181 YSSIAHMGWMLAILPYNPSLTLLNLTIYILMTISMFLMLMTSSYTTINSISMAWNKHPIM 240 |
| YSSIAHMGWMLAILPYNPSLTLLNL IYI++T MF+ LM ++ TINSIS+ WNK P M |
| Sbjct 181 YSSIAHMGWMLAILPYNPSLTLLNLMIYIILTAPMFMALMLNNSMTINSISLLWNKTPAM 240 |
|  |
| Query 241 IPLMATVLLSLGGLPPLSGFMPKWLIITELLKNNSTPAASLIALMALLNLFFYTRLIYST 300 |
| + +++ +LLSLGGLPPL+GF+PKW+IITEL+KNN A+L+A+MALLNLFFYTRLIYST |
| Sbjct 241 LTMISLMLLSLGGLPPLTGFLPKWIIITELMKNNCLIMATLMAMMALLNLFFYTRLIYST 300 |
|  |
| Query 301 SLTIFPTNNNSKLIPQQLASKHFPMMPLLMTLSTMILPLTPLLI 344 |
| SLT+FPTNNNSK++ Q +K M L +STM LPL P LI |
| Sbjct 301 SLTMFPTNNNSKMMTHQTKTKPNLMFSTLAIMSTMTLPLAPQLI 344 |
|  |
| **Query #44: AEP83261.1 NADH dehydrogenase subunit 1 [Acomys cahirinus] Query ID: lcl\|Query_8047 Length: 318** |
| **Sequence ID: P03888.3 Length: 318** |
|  |
| Query 4 INTLTLLIPILMAMAFLTLVERKMLGYMQLRKGPNIVGPYGILQPFADAMKLIIKEPLRP 63 |
| IN LTLL+PIL+AMAFLTLVERK+LGYMQLRKGPNIVGPYGILQPFADAMKL +KEP+RP |
| Sbjct 4 INILTLLVPILIAMAFLTLVERKILGYMQLRKGPNIVGPYGILQPFADAMKLFMKEPMRP 63 |
|  |
| Query 64 LSTSVVLFIIAPTLSLTLAFSLWIPMPMPHPLANMNLSTLFILALSSLSVYSILWSGWAS 123 |
| L+TS+ LFIIAPTLSLTLA SLW+P+PMPHPL N+NL LFILA SSLSVYSILWSGWAS |
| Sbjct 64 LTTSMSLFIIAPTLSLTLALSLWVPLPMPHPLINLNLGILFILATSSLSVYSILWSGWAS 123 |
|  |
| Query 124 NSKYSLFGAIRAVAQTISYEVTMAIILLSVLLMNGSFSLQTLIITQEHMWLIIPTWPLAM 183 |
| NSKYSLFGA+RAVAQTISYEVTMAIILLSVLLMNGS+SLQTLI TQEHMWL++P WP+AM |
| Sbjct 124 NSKYSLFGALRAVAQTISYEVTMAIILLSVLLMNGSYSLQTLITTQEHMWLLLPAWPMAM 183 |
|  |
| Query 184 MWYISTLAETNRAPFDLTEGESELVSGFNVEYSAGPFALFFMAEYTNIILMNALSSIMFM 243 |
| MW+ISTLAETNRAPFDLTEGESELVSGFNVEY+AGPFALFFMAEYTNIILMNAL++I+F+ |
| Sbjct 184 MWFISTLAETNRAPFDLTEGESELVSGFNVEYAAGPFALFFMAEYTNIILMNALTTIIFL 243 |
|  |
| Query 244 APLYYADHPETFTTNFMLKTLMLTSLFLWVRASYPRFRYDHLMHLLWKNFLPLTLALCMW 303 |
| PLYY + PE ++TNFM++ L+L+S FLW+RASYPRFRYD LMHLLWKNFLPLTLALCMW |
| Sbjct 244 GPLYYINLPELYSTNFMMEALLLSSTFLWIRASYPRFRYDQLMHLLWKNFLPLTLALCMW 303 |
|  |
| Query 304 YVSLPIFLSSIPPY 317 |
| ++SLPIF + +PPY |
| Sbjct 304 HISLPIFTAGVPPY 317 |
|  |
| **Query #45: CBN72533.1 recombination activating gene 2, partial [Acomys cahirinus] Query ID: lcl\|Query_8048 Length: 309** |
| **Sequence ID: P21784.2 Length: 527** |
|  |
| Query 1 VLFGGRSYMPSTQRTTEKWNSVADCLPHVFLIDFEFGCATSYILPELQDGLSFHVSIAKN 60 |
| VLFGGRSYMPSTQRTTEKWNSVADCLPHVFLIDFEFGCATSYILPELQDGLSFHVSIA+N |
| Sbjct 154 VLFGGRSYMPSTQRTTEKWNSVADCLPHVFLIDFEFGCATSYILPELQDGLSFHVSIARN 213 |
|  |
| Query 61 DTIYILGGHSLTNNIRPANLYRIRVDLPLGSPAVNCTVLPGGISVSSAILTQTNNDEFVI 120 |
| DT+YILGGHSL +NIRPANLYRIRVDLPLG+PAVNCTVLPGGISVSSAILTQTNNDEFVI |
| Sbjct 214 DTVYILGGHSLASNIRPANLYRIRVDLPLGTPAVNCTVLPGGISVSSAILTQTNNDEFVI 273 |
|  |
| Query 121 VGGYQLENQKRMVCSIVSLRDNTIEISEMETPDWTPDIKHSKIWFGSNMGNGTVVLGIPG 180 |
| VGGYQLENQKRMVCS+VSL DNTIEISEMETPDWT DIKHSKIWFGSNMGNGT+ LGIPG |
| Sbjct 274 VGGYQLENQKRMVCSLVSLGDNTIEISEMETPDWTSDIKHSKIWFGSNMGNGTIFLGIPG 333 |
|  |
| Query 181 DNKQAGAEAFYFYMLKCSEDDXSEEQKIFTNSQTSTEDPGDSTPFEDSEEFCFSAEATSF 240 |
| DNKQA +EAFYFY L+CSE+D SE+QKI +NSQTSTEDPGDSTPFEDSEEFCFSAEATSF |
| Sbjct 334 DNKQAMSEAFYFYTLRCSEEDLSEDQKIVSNSQTSTEDPGDSTPFEDSEEFCFSAEATSF 393 |
|  |
| Query 241 DGDDEFDTYNEDDEEDESVTGYWITCCPTCDVDINTWVPFYSTELNKPAMIYCSHGDGHW 300 |
| DGDDEFDTYNEDDE+DESVTGYWITCCPTCDVDINTWVPFYSTELNKPAMIYCSHGDGHW |
| Sbjct 394 DGDDEFDTYNEDDEDDESVTGYWITCCPTCDVDINTWVPFYSTELNKPAMIYCSHGDGHW 453 |
|  |
| Query 301 VHAQCMDL 308 |
| VHAQCMDL |
| Sbjct 454 VHAQCMDL 461 |
|  |
| **Query #46: YP_007626818.1 NADH dehydrogenase subunit 4L [Acomys cahirinus] Query ID: lcl\|Query_8049 Length: 98** |
| **Sequence ID: P03903.2 Length: 98** |
|  |
| Query 1 MSPIYINLTLAFSLSLLGTLMFRSHLMSSLLCLEGMMLSLFIMTSASSLNTNSMTFFPIP 60 |
| M + NLT+AFSLSLLGTLMFRSHLMS+LLCLEGM+LSLFIMTS +SLN+NSM+ PIP |
| Sbjct 1 MPSTFFNLTMAFSLSLLGTLMFRSHLMSTLLCLEGMVLSLFIMTSVTSLNSNSMSSMPIP 60 |
|  |
| Query 61 ITILVFAACEAAVGLALLVKVSSTYGTDYVQNLNALQC 98 |
| ITILVFAACEAAVGLALLVKVS+TYGTDYVQNLN LQC |
| Sbjct 61 ITILVFAACEAAVGLALLVKVSNTYGTDYVQNLNLLQC 98 |
|  |
| **Query #47: AEP83271.1 NADH dehydrogenase subunit 5 [Acomys cahirinus] Query ID: lcl\|Query_8050 Length: 610** |
| **Sequence ID: P03921.3 Length: 607** |
|  |
| Query 23 LTHLMKHIKFPHYATLSIKNAFLISLVPLATLLHYNIESMISSWHWMTINTINLSISFKI 82 |
| +++L+KHI FP Y T SIK +F+ISL+PL H N+E MI++WHW+T+N++ L +SFK |
| Sbjct 23 MSNLIKHINFPLYTTTSIKFSFIISLLPLLMFFHNNMEYMITTWHWVTMNSMELKMSFKT 82 |
|  |
| Query 83 DFFSILFLSVALFVTWSIMEFSSWYMHSDPDLNRFIKYLLLFLITMIILTSANNLFQLFI 142 |
| DFFSILF SVALFVTWSIM+FSSWYMHSDP++NRFIKYL LFLITM+ILTSANN+FQLFI |
| Sbjct 83 DFFSILFTSVALFVTWSIMQFSSWYMHSDPNINRFIKYLTLFLITMLILTSANNMFQLFI 142 |
|  |
| Query 143 GWEGVGIMSFLLIGWWYGRTEANTAALQAILYNRIGDIGLILAMTWFCLNYNSWEMQQIL 202 |
| GWEGVGIMSFLLIGWWYGRT+ANTAALQAILYNRIGDIG ILAM WF LN NSWE+QQI+ |
| Sbjct 143 GWEGVGIMSFLLIGWWYGRTDANTAALQAILYNRIGDIGFILAMVWFSLNMNSWELQQIM 202 |
|  |
| Query 203 MHNNN-NTIPLMGLLLAATGKSAQFGLHPWLPSAMEGPTPVSALLHSSTMVVAGVFLMIR 261 |
| NNN N IPLMGLL+AATGKSAQFGLHPWLPSAMEGPTPVSALLHSSTMVVAG+FL++R |
| Sbjct 203 FSNNNDNLIPLMGLLIAATGKSAQFGLHPWLPSAMEGPTPVSALLHSSTMVVAGIFLLVR 262 |
|  |
| Query 262 FFPLTSNNSTILTLMLCLGSITTLFTAICALTQNDIKKIVAFSTSSQLGLMMVTLGINQP 321 |
| F PLT+NN+ ILT MLCLG++TTLFTAICALTQNDIKKI+AFSTSSQLGLMMVTLG+NQP |
| Sbjct 263 FHPLTTNNNFILTTMLCLGALTTLFTAICALTQNDIKKIIAFSTSSQLGLMMVTLGMNQP 322 |
|  |
| Query 322 YLAFLHICMHAFFKAMLFMCSGSIIHNLNDEQDIRKMGSTTQTLPFTTSCLTIGSLALTG 381 |
| +LAFLHIC HAFFKAMLFMCSGSIIH+L DEQDIRKMG+ T+ +PFT+SCL IGSLALTG |
| Sbjct 323 HLAFLHICTHAFFKAMLFMCSGSIIHSLADEQDIRKMGNITKIMPFTSSCLVIGSLALTG 382 |
|  |
| Query 382 MPFLTGFYSKDLIIEAVNTSNTNAWALLITLIATSLTAAYSMRIIYFVTMTKPRYPPMMS 441 |
| MPFLTGFYSKDLIIEA+NT NTNAWALLITLIATS+TA YSMRIIYFVTMTKPR+PP++S |
| Sbjct 383 MPFLTGFYSKDLIIEAINTCNTNAWALLITLIATSMTAMYSMRIIYFVTMTKPRFPPLIS 442 |
|  |
| Query 442 MSENNPKLINPIKRLALGSIFAGYIISHNIPPINTQTMTMPWQLKMTALFITITGLLVAL 501 |
| ++EN+P L+NPIKRLA GSIFAG++IS+NIPP + +TMPW LK TAL I++ G L+AL |
| Sbjct 443 INENDPDLMNPIKRLAFGSIFAGFVISYNIPPTSIPVLTMPWFLKTTALIISVLGFLIAL 502 |
|  |
| Query 502 ELNNLTSNLTPNKTSPYSTFSALLGYFPTITHRLIPAKTLNLGMKISLNTLDQTWLETAI 561 |
| ELNNLT L+ NK +PYS+FS LLG+FP+I HR+ P K+LNL +K SL LD WLE I |
| Sbjct 503 ELNNLTMKLSMNKANPYSSFSTLLGFFPSIIHRITPMKSLNLSLKTSLTLLDLIWLEKTI 562 |
|  |
| Query 562 PKSTSTLHTMMSKHISNQKGLIKIYFLSF 590 |
| PKSTSTLHT M+ +NQKGLIK+YF+SF |
| Sbjct 563 PKSTSTLHTNMTTLTTNQKGLIKLYFMSF 591 |
|  |
| **Query #48: CBN72534.1 von Willebrand factor, partial [Acomys cahirinus] Query ID: lcl\|Query_8051 Length: 421** |
| **Sequence ID: Q8CIZ8.2 Length: 2813** |
|  |
| Query 1 QEPGGLVVPPTDVPTSSTTPYVENTPEPPLQSFSCSKLLDLVFLLDGSSSLSEAEFEKLK 60 |
| QEPGGLV PPTD P SSTTPYVE+TPEPPL +F CSKLLDLVFLLDGSS LSEAEFE LK |
| Sbjct 1238 QEPGGLVAPPTDAPVSSTTPYVEDTPEPPLHNFYCSKLLDLVFLLDGSSMLSEAEFEVLK 1297 |
|  |
| Query 61 AFVMGTMEKLHISQKRIRVAVVEYHDGSHAYIELKARKRPSELRRITSQVKYAGSEVAST 120 |
| AFV+G ME+LHISQKRIRVAVVEYHDGS AY+ELKARKRPSELRRITSQ+KY GS+VAST |
| Sbjct 1298 AFVVGMMERLHISQKRIRVAVVEYHDGSRAYLELKARKRPSELRRITSQIKYTGSQVAST 1357 |
|  |
| Query 121 SEVLKYTLFQIFGRIDRPEASRIALLLTASQEPSQTARTLTRYVQALKKKKVIVVPVGIG 180 |
| SEVLKYTLFQIFG+IDRPEAS I LLLTASQEP + AR L RYVQ LKKKKVIV+PVGIG |
| Sbjct 1358 SEVLKYTLFQIFGKIDRPEASHITLLLTASQEPPRMARNLVRYVQGLKKKKVIVIPVGIG 1417 |
|  |
| Query 181 PRVSLKQIRLIERQAPENKAFSFSGVDELEQKRSELINYLCDLVPGAPAPTQPPQVAQIT 240 |
| P SLKQIRLIE+QAPENKAF SGVDELEQ+R E+++YLCDL P APAPTQPPQVA +T |
| Sbjct 1418 PHASLKQIRLIEKQAPENKAFLLSGVDELEQRRDEIVSYLCDLAPEAPAPTQPPQVAHVT 1477 |
|  |
| Query 241 VGPEISGVSSPGPKRKSMVLDVVFVLEGSDKVGEANFNKSKEFLEEVIRRMDVGQGGIHI 300 |
| V P I+G+SSPGPKRKSMVLDVVFVLEGSD+VGEANFNKSKEF+EEVI+RMDV I |
| Sbjct 1478 VSPGIAGISSPGPKRKSMVLDVVFVLEGSDEVGEANFNKSKEFVEEVIQRMDVSPDATRI 1537 |
|  |
| Query 301 TVLQYSYTVTVEYTFNEAQAKEDVLRHVREIRYHGGNRTNTGLALQYVSEHSFSPSQGDR 360 |
| +VLQYSYTVT+EY FN AQ+KE+VLRHVREIRY GGNRTNTG ALQY+SEHSFSPSQGDR |
| Sbjct 1538 SVLQYSYTVTMEYAFNGAQSKEEVLRHVREIRYQGGNRTNTGQALQYLSEHSFSPSQGDR 1597 |
|  |
| Query 361 QQAPNLVYMVTGNPASDEIKRLPGDIQIVPIGVGPHANLQELERIGRPNVPIFIQDFETL 420 |
| +APNLVYMVTGNPASDEIKRLPGDIQ+VPIGVGPHAN+QELERI RP PIFI+DFETL |
| Sbjct 1598 VEAPNLVYMVTGNPASDEIKRLPGDIQVVPIGVGPHANMQELERISRPIAPIFIRDFETL 1657 |
|  |
| Query 421 P 421 |
| P |
| Sbjct 1658 P 1658 |
|  |
| **Query #49: CBN72532.1 interphotoreceptor retinoid-binding protein, partial [Acomys cahirinus] Query ID: lcl\|Query_8052 Length: 424** |
| **Sequence ID: P49194.3 Length: 1234** |
|  |
| Query 1 PENLMGMQATIEQAMKSREILGISDPQTLAQVLTAGVQSSLNDPRLLISYEPSTLEAPQQ 60 |
| PENLMGMQA IEQAMKS EILGISDPQTLAQVLTAGVQSSL+DPRL ISYEPSTLEAPQQ |
| Sbjct 42 PENLMGMQAAIEQAMKSHEILGISDPQTLAQVLTAGVQSSLSDPRLFISYEPSTLEAPQQ 101 |
|  |
| Query 61 VPELTNLTREDLLARIQRNIHHEVLEGNVGYLRVDDLPGQEVLSELGEFLVTHVWRQLMS 120 |
| P LTNLTRE+LLA+IQRNI HEVLEGNVGYLRVDDLPGQEVLSELGEFLV+HVWRQLM |
| Sbjct 102 APVLTNLTREELLAQIQRNIRHEVLEGNVGYLRVDDLPGQEVLSELGEFLVSHVWRQLMG 161 |
|  |
| Query 121 TSSLVLDLRHCAGGHVSGIPYVVSYLHPGNTVMHVDTIYDRPSNTTTEIWTLPRVLGERY 180 |
| TSSLVLDLRHC+GGH SGIPYV+SYLHPGNTVMHVDT+YDRPSNTTTEIWTLP VLGERY |
| Sbjct 162 TSSLVLDLRHCSGGHFSGIPYVISYLHPGNTVMHVDTVYDRPSNTTTEIWTLPEVLGERY 221 |
|  |
| Query 181 SADKDVVVLTSGRTGGVAEDIAYILKQMRRAIVVGERTEGGALDLQKLRIGKSNFFLTVP 240 |
| SADKDVVVLTSG TGGVAEDIAYILKQMRRAIVVGERTEGGALDLQKLRIG+SNFFLTVP |
| Sbjct 222 SADKDVVVLTSGHTGGVAEDIAYILKQMRRAIVVGERTEGGALDLQKLRIGQSNFFLTVP 281 |
|  |
| Query 241 VSRSLGPLGGGGQTWEGSGVLPCVGTPAEQALEKALAILTLRRALPGVVLRLQEALQDYY 300 |
| VSRSLGPLGGGGQTWEGSGVLPCVGTPAEQALEKALAILTLRRALPGVVLRLQEALQDYY |
| Sbjct 282 VSRSLGPLGGGGQTWEGSGVLPCVGTPAEQALEKALAILTLRRALPGVVLRLQEALQDYY 341 |
|  |
| Query 301 TLVDRVPGLLHHLASMDYSAVVSEEDLVTKLNAGLQAVSEDPRLLVRATGPRETSCRPET 360 |
| TLVDRVPGLLHHLASMDYSAVVSEEDLVTKLNAGLQAVSEDPRLLVRATGPR++S RPET |
| Sbjct 342 TLVDRVPGLLHHLASMDYSAVVSEEDLVTKLNAGLQAVSEDPRLLVRATGPRDSSSRPET 401 |
|  |
| Query 361 GSNEPPAAVSEVPEEEAARRSLVDSVFQVSVLPGNVGYLRFDRFADAPVLRALGPYVLHQ 420 |
| G NE PAA EVP EE ARR+LVDSVFQVSVLPGNVGYLRFDRFADA VL LGPYVL Q |
| Sbjct 402 GPNESPAATPEVPTEEDARRALVDSVFQVSVLPGNVGYLRFDRFADAAVLETLGPYVLKQ 461 |
|  |
| Query 421 VWEP 424 |
| VWEP |
| Sbjct 462 VWEP 465 |
|  |
| **Query #50: YP_007626817.1 NADH dehydrogenase subunit 3 [Acomys cahirinus] Query ID: lcl\|Query_8053 Length: 115** |
| **Sequence ID: P03899.3 Length: 115** |
|  |
| Query 1 MNMLLAVTINITLSLLLITVAFWMPQTNIYTEKANPYECGFDPINSARLPFSMKFFLVAI 60 |
| MN+ + INI LSL LI VAFW+PQ N+Y+EKANPYECGFDP +SARLPFSMKFFLVAI |
| Sbjct 1 MNLYTVIFINILLSLTLILVAFWLPQMNLYSEKANPYECGFDPTSSARLPFSMKFFLVAI 60 |
|  |
| Query 61 TFLLFDLEIALLLPIPWAIQTPKVHYMMLYAFLLISILSLGLAYEWMHKGLEWTE 115 |
| TFLLFDLEIALLLP+PWAIQT K MM+ AF+L++ILSLGLAYEW KGLEWTE |
| Sbjct 61 TFLLFDLEIALLLPLPWAIQTIKTSTMMIMAFILVTILSLGLAYEWTQKGLEWTE 115 |
|  |
| **Query #51: CBN72530.1 cannabinoid receptor 1, partial [Acomys cahirinus] Query ID: lcl\|Query_8054 Length: 364** |
| **Sequence ID: P47746.1 Length: 473** |
|  |
| Query 1 SKLGYFPQKFPLTSFRGSPFQEKMTAGDNSQLVPAVDATNITEFYNKSLSSFKENEESIQ 60 |
| SKLGYFPQKFPLTSFRGSPFQEKMTAGDNS LVPA D TNITEFYNKSLSSFKENE++IQ |
| Sbjct 39 SKLGYFPQKFPLTSFRGSPFQEKMTAGDNSPLVPAGDTTNITEFYNKSLSSFKENEDNIQ 98 |
|  |
| Query 61 CGENFMDMECFMILNPSQQLAIAVLSLTLGTFTVLENLLVLCVILHSRSLRCRPSYHFIG 120 |
| CGENFMDMECFMILNPSQQLAIAVLSLTLGTFTVLENLLVLCVILHSRSLRCRPSYHFIG |
| Sbjct 99 CGENFMDMECFMILNPSQQLAIAVLSLTLGTFTVLENLLVLCVILHSRSLRCRPSYHFIG 158 |
|  |
| Query 121 SLAVADLLGSVIFVYSFVDFHVFHRKDSPNVFLFKLGGVTASFTASVGSLFLTAIDRYIS 180 |
| SLAVADLLGSVIFVYSFVDFHVFHRKDSPNVFLFKLGGVTASFTASVGSLFLTAIDRYIS |
| Sbjct 159 SLAVADLLGSVIFVYSFVDFHVFHRKDSPNVFLFKLGGVTASFTASVGSLFLTAIDRYIS 218 |
|  |
| Query 181 IHRPLAYKRIVTRPKAVVAFCLMWTIAIVIAVLPLLGWNCKKLQSVCSDIFPLIDETYLM 240 |
| IHRPLAYKRIVTRPKAVVAFCLMWTIAIVIAVLPLLGWNCKKLQSVCSDIFPLIDETYLM |
| Sbjct 219 IHRPLAYKRIVTRPKAVVAFCLMWTIAIVIAVLPLLGWNCKKLQSVCSDIFPLIDETYLM 278 |
|  |
| Query 241 FWIGVTSVLLLFIVYAYMYILWKAHSHAVRMIQRGTQKSIIIHTSEDGKVQVTRPDQARM 300 |
| FWIGVTSVLLLFIVYAYMYILWKAHSHAVRMIQRGTQKSIIIHTSEDGKVQVTRPDQARM |
| Sbjct 279 FWIGVTSVLLLFIVYAYMYILWKAHSHAVRMIQRGTQKSIIIHTSEDGKVQVTRPDQARM 338 |
|  |
| Query 301 DIRLAKTLVLILVVLIICWGPLLAIMVYDVFGKMNKLIKTVFAFCSMLCLLNSTVNPIIY 360 |
| DIRLAKTLVLILVVLIICWGPLLAIMVYDVFGKMNKLIKTVFAFCSMLCLLNSTVNPIIY |
| Sbjct 339 DIRLAKTLVLILVVLIICWGPLLAIMVYDVFGKMNKLIKTVFAFCSMLCLLNSTVNPIIY 398 |
|  |
| Query 361 ALRS 364 |
| ALRS |
| Sbjct 399 ALRS 402 |
|  |
| **Query #52: CBN72529.1 alpha 2B adrenergic receptor, partial [Acomys cahirinus] Query ID: lcl\|Query_8055 Length: 393** |
| **Sequence ID: P30545.2 Length: 450** |
|  |
| Query 8 FLILFTIFGNALVILAVLTSRSLRAPQNLFLVSLAAADILVATLIIPFSLANELLGYWYF 67 |
| FLILFTIFGNALVILAVLTSRSLRAPQNLFLVSLAAADILVATLIIPFSLANELLGYWYF |
| Sbjct 21 FLILFTIFGNALVILAVLTSRSLRAPQNLFLVSLAAADILVATLIIPFSLANELLGYWYF 80 |
|  |
| Query 68 WRTWCEVYLALDVLFCTSSIVHLCAISLDRYWAVSRALEYNSKRTPRRIKCIILTVWLIA 127 |
| WR WCEVYLALDVLFCTSSIVHLCAISLDRYWAVSRALEYNSKRTPRRIKCIILTVWLIA |
| Sbjct 81 WRAWCEVYLALDVLFCTSSIVHLCAISLDRYWAVSRALEYNSKRTPRRIKCIILTVWLIA 140 |
|  |
| Query 128 AAISLPPLVYKGDQRPEPHGLPQCELNQEAWYILASSFGSFFAPCLIMILVYLRIYVIAK 187 |
| A ISLPPL+YKGDQRPEPHGLPQCELNQEAWYILASS GSFFAPCLIMILVYLRIYVIAK |
| Sbjct 141 AVISLPPLIYKGDQRPEPHGLPQCELNQEAWYILASSIGSFFAPCLIMILVYLRIYVIAK 200 |
|  |
| Query 188 RSHCRGSRAKRASGEGESKKPR--PVAGGAPTSAKVPTLASPLSSVGEANGHPKPPSEKE 245 |
| RSHCRG AKR SGEGESKKPR P AGG P SAKVPTL SPLSSVGEANGHPKPP EKE |
| Sbjct 201 RSHCRGLGAKRGSGEGESKKPRPGPAAGGVPASAKVPTLVSPLSSVGEANGHPKPPREKE 260 |
|  |
| Query 246 EGETPEDAESRALPSSWSALPRPGQSQKKDISGAIAEGVEEEDEEGVEEHEPQTLPASPG 305 |
| EGETPED E+RALP +WSALPR Q QKK SGA AE EEDEE VEE EPQTLPASP |
| Sbjct 261 EGETPEDPEARALPPNWSALPRSVQDQKKGTSGATAEKGAEEDEEEVEECEPQTLPASPA 320 |
|  |
| Query 306 SGCSPPLQQPQASQVLATLRGQVLLSRNVGAASGQWWRRRTQLSRERRFTFVLAVVIGVF 365 |
| S +PPLQQPQ S+VLATLRGQVLLS+NVG ASGQWWRRRTQLSRE+RFTFVLAVVIGVF |
| Sbjct 321 SVFNPPLQQPQTSRVLATLRGQVLLSKNVGVASGQWWRRRTQLSREKRFTFVLAVVIGVF 380 |
|  |
| Query 366 VVCWFPFFFSYSLGAICPQHCKVPHGIF 393 |
| VVCWFPFFFSYSLGAICPQHCKVPHG+F |
| Sbjct 381 VVCWFPFFFSYSLGAICPQHCKVPHGLF 408 |
|  |
| **Query #53: AAB87156.1 NADH dehydrogenase subunit 4, partial [Acomys cahirinus] Query ID: lcl\|Query_8056 Length: 207** |
| **Sequence ID: P03911.1 Length: 459** |
|  |
| Query 1 MLKIIMASTMLLPLTWLSKNKYMWINVTSHSFMISLTSLTLLWQ-NENCTTFSISLFSDH 59 |
| MLKII+ S MLLPLTWLS K W NVTS+SF+ISLTSLTLLWQ +EN FS SD |
| Sbjct 1 MLKIILPSLMLLPLTWLSSPKKTWTNVTSYSFLISLTSLTLLWQTDENYKNFSNMFSSDP 60 |
|  |
| Query 60 ISTPLIILTTWLLPLMLLASQNHMKKENPTHKKLYITMLILLQILLILTFSATEMIMFYI 119 |
| +STPLIILT WLLPLML+ASQNH+KK+N +KLYI+MLI LQILLI+TFSATE+IMFYI |
| Sbjct 61 LSTPLIILTAWLLPLMLMASQNHLKKDNNVLQKLYISMLISLQILLIMTFSATELIMFYI 120 |
|  |
| Query 120 MFEATLIPTLIIITRWGNQTERLNAGLYFLFYTLVGSIPLLIALIMTQNSTGTLNFMLMT 179 |
| +FEATLIPTLIIITRWGNQTERLNAG+YFLFYTL+GSIPLLIALI+ QN GTLN M+++ |
| Sbjct 121 LFEATLIPTLIIITRWGNQTERLNAGIYFLFYTLIGSIPLLIALILIQNHVGTLNLMILS 180 |
|  |
| Query 180 LNSQTMNTTWSNNILWLTCMLAFLIKMP 207 |
| + T++ +WSNN+LWL CM+AFLIKMP |
| Sbjct 181 FTTHTLDASWSNNLLWLACMMAFLIKMP 208 |
|  |
| **Query #54: AEF14392.1 NADH dehydrogenase subunit I, partial [Acomys cahirinus] Query ID: lcl\|Query_8057 Length: 315** |
| **Sequence ID: P03888.3 Length: 318** |
|  |
| Query 1 INTLTLLIPILMAMAFLTLVERKMLGYMQLRKGPNIVGPYGILQPFADAMKLIIKEPLRP 60 |
| IN LTLL+PIL+AMAFLTLVERK+LGYMQLRKGPNIVGPYGILQPFADAMKL +KEP+RP |
| Sbjct 4 INILTLLVPILIAMAFLTLVERKILGYMQLRKGPNIVGPYGILQPFADAMKLFMKEPMRP 63 |
|  |
| Query 61 LSTSVVLFIIAPTLSLTLALSLWIPMPMPHPLANMNLSTLFILALSSLSVYSILWSGWAS 120 |
| L+TS+ LFIIAPTLSLTLALSLW+P+PMPHPL N+NL LFILA SSLSVYSILWSGWAS |
| Sbjct 64 LTTSMSLFIIAPTLSLTLALSLWVPLPMPHPLINLNLGILFILATSSLSVYSILWSGWAS 123 |
|  |
| Query 121 NSKYSLFGAIRAVAQTISYEVTMAIILLSVLLMNGSFSLQTLIITQEHMWLIIPTWPLAM 180 |
| NSKYSLFGA+RAVAQTISYEVTMAIILLSVLLMNGS+SLQTLI TQEHMWL++P WP+AM |
| Sbjct 124 NSKYSLFGALRAVAQTISYEVTMAIILLSVLLMNGSYSLQTLITTQEHMWLLLPAWPMAM 183 |
|  |
| Query 181 MWYISTLAETNRAPFDLTEGESELVSGFNVEYSAGPFALFFMAEYTNIILMNALSSIMFM 240 |
| MW+ISTLAETNRAPFDLTEGESELVSGFNVEY+AGPFALFFMAEYTNIILMNAL++I+F+ |
| Sbjct 184 MWFISTLAETNRAPFDLTEGESELVSGFNVEYAAGPFALFFMAEYTNIILMNALTTIIFL 243 |
|  |
| Query 241 APLYYADHPETFTTNFMLKTLMLTSLFLWVRASYPRFRYDHLMHLLWKNFLPLTLALCMW 300 |
| PLYY + PE ++TNFM++ L+L+S FLW+RASYPRFRYD LMHLLWKNFLPLTLALCMW |
| Sbjct 244 GPLYYINLPELYSTNFMMEALLLSSTFLWIRASYPRFRYDQLMHLLWKNFLPLTLALCMW 303 |
|  |
| Query 301 YVSLPIFLSSIPPY 314 |
| ++SLPIF + +PPY |
| Sbjct 304 HISLPIFTAGVPPY 317 |
|  |
| **Query #55: ACS44656.1 pancreas duodenal homeobox-1 [Acomys cahirinus] Query ID: lcl\|Query_8058 Length: 286** |
| **Sequence ID: P52946.1 Length: 284** |
|  |
| Query 1 MNSEEQYYAATQLYKDPCAFQRGPVPEFSANPPACLYMGCQPPPPPPPQFAGALGTLEQG 60 |
| MNSEEQYYAATQLYKDPCAFQRGPVPEFSANPPACLYMG QPPPPPPPQF +LG+LEQG |
| Sbjct 1 MNSEEQYYAATQLYKDPCAFQRGPVPEFSANPPACLYMGRQPPPPPPPQFTSSLGSLEQG 60 |
|  |
| Query 61 SPPDISPYEVPPLA-DDPAGAHLHHHLPAQLGLTQPPSGPFPNGTEPGGLEEPSRVQLPF 119 |
| SPPDISPYEVPPLA DDPAGAHLHHHLPAQLGL PP GPFPNGTEPGGLEEP+RVQLPF |
| Sbjct 61 SPPDISPYEVPPLASDDPAGAHLHHHLPAQLGLAHPPPGPFPNGTEPGGLEEPNRVQLPF 120 |
|  |
| Query 120 PWMKSTKAHAWKGQWAGGAYAAEPEENKRTRTAYTRAQLLELEKEFLFNKYISRPRRVEL 179 |
| PWMKSTKAHAWKGQWAGGAY AEPEENKRTRTAYTRAQLLELEKEFLFNKYISRPRRVEL |
| Sbjct 121 PWMKSTKAHAWKGQWAGGAYTAEPEENKRTRTAYTRAQLLELEKEFLFNKYISRPRRVEL 180 |
|  |
| Query 180 AVMLNLTERHIKIWFQNRRMKWKKEEDKKRSSGTTSGGGGGGGGEEPEQDCAVSSGEELL 239 |
| AVMLNLTERHIKIWFQNRRMKWKKEEDKKRSSGT SGGGGG EQDCAV+SGEELL |
| Sbjct 181 AVMLNLTERHIKIWFQNRRMKWKKEEDKKRSSGTPSGGGGGEEP---EQDCAVTSGEELL 237 |
|  |
| Query 240 ALPPPPPPGGAVPPSVPAAAREGRLPPGLSASPQPSGIAPLRPQEPR 286 |
| A+PP PPPGGAVPP VPAA REG LP GLS SPQPS IAPLRPQEPR |
| Sbjct 238 AVPPLPPPGGAVPPGVPAAVREGLLPSGLSVSPQPSSIAPLRPQEPR 284 |
|  |
| **Query #56: AAB87155.1 NADH dehydrogenase subunit 4L [Acomys cahirinus] Query ID: lcl\|Query_8059 Length: 98** |
| **Sequence ID: P03903.2 Length: 98** |
|  |
| Query 1 MSPIFINLTLAFSLSLLGTLMFRSHLMSSLLCLEGMMLSLFIMTSASSLNTNSMTFFPIP 60 |
| M F NLT+AFSLSLLGTLMFRSHLMS+LLCLEGM+LSLFIMTS +SLN+NSM+ PIP |
| Sbjct 1 MPSTFFNLTMAFSLSLLGTLMFRSHLMSTLLCLEGMVLSLFIMTSVTSLNSNSMSSMPIP 60 |
|  |
| Query 61 ITILVFAACEAAVGLALLVKVSSTYGTDYVQNLNALQC 98 |
| ITILVFAACEAAVGLALLVKVS+TYGTDYVQNLN LQC |
| Sbjct 61 ITILVFAACEAAVGLALLVKVSNTYGTDYVQNLNLLQC 98 |
|  |
| **Query #57: CBN72531.1 growth hormone receptor, partial [Acomys cahirinus] Query ID: lcl\|Query_8060 Length: 303** |
| **Sequence ID: P16882.1 Length: 650** |
|  |
| Query 1 GIHDNYKPDFYNDDSWVEFIELDID--DADEKTEGSDTDRLLSNDHEKSINILGAKDDDS 58 |
| GIHDNYKPDFYNDDSWVEFIELDID D DEKTEGSDTDRLLSNDHEKS ILGAKDDDS |
| Sbjct 336 GIHDNYKPDFYNDDSWVEFIELDIDEADVDEKTEGSDTDRLLSNDHEKSAGILGAKDDDS 395 |
|  |
| Query 59 GRTSCYDPDILDTDFHTNDMCDGTSEFAQSQKLRTEADLLCLDQKNLKNSASDASLGSLH 118 |
| GRTSCYDPDILDTDFHT+DMCDGT +F QSQKL EADLLCLDQKNLKN DASLGSLH |
| Sbjct 396 GRTSCYDPDILDTDFHTSDMCDGTLKFRQSQKLNMEADLLCLDQKNLKNLPYDASLGSLH 455 |
|  |
| Query 119 PCTTPTVEEDKPQALVSSETESTHQLASTPMSNPASLANTDFYAQVSDITPAGSVVLSPG 178 |
| P T TVEE+KPQ L+SSETE+THQLASTPMSNP SLAN DFYAQVSDITPAG VLSPG |
| Sbjct 456 PSITQTVEENKPQPLLSSETEATHQLASTPMSNPTSLANIDFYAQVSDITPAGGDVLSPG 515 |
|  |
| Query 179 QKIKAGIARCDNQQPEVGAPGQENYGINSSYXCESDAKKCIAVAPHMEAMSCLKPSFNQE 238 |
| QKIKAGIA+ N Q EV P QENY +NS+Y CESDAKKCIAVA MEA SC+KPSFNQE |
| Sbjct 516 QKIKAGIAQ-GNTQREVATPCQENYSMNSAYFCESDAKKCIAVARRMEATSCIKPSFNQE 574 |
|  |
| Query 239 DIYITTESLTTTARMSKTAELAPDAEMAVPDYTAVHTVQSPQGLILNAAALPLPDKKKLL 298 |
| DIYITTESLTTTA+MS+TA++APDAEM+VPDYT VHTVQSP+GLILNA ALPLPDKK |
| Sbjct 575 DIYITTESLTTTAQMSETADIAPDAEMSVPDYTTVHTVQSPRGLILNATALPLPDKKNFP 634 |
|  |
| Query 299 SSCGY 303 |
| SSCGY |
| Sbjct 635 SSCGY 639 |
|  |
| All *Acomys cahirinus* proteins available from the NCBI protein database (272 protein belonging to 57 Identical Protein Groups) were searched, using BLASTP, against Mus musculus SwissProt database. Top sequence matches are shown only, the identity of Mus proteins is indicated above each of the sequence alignments. |

**Table S2.** Proteins identified in *Acomys* and *Mus* skin at day 0

|  |  |  |  | ***Acomys*** | ***Mus*** |
| --- | --- | --- | --- | --- | --- |
|  |  |  |  | **0 day** | **0 day** |
| **Location** | **Accession** | **Protein Description** | **Gene** | **Quantitative Value** | |
| **Both of *Acomys* and *Mus*** | **A1L317** | **Keratin, type I cytoskeletal 24** | **Krt24** | **7.70** | **5.77** |
|  | **A2AQ07** | **Tubulin beta-1 chain** | **Tubb1** | **5.05** | **6.02** |
|  | **A2AQP0** | **Myosin-7B** | **Myh7b** | **8.74** | **8.17** |
|  | **A6BLY7** | **Keratin, type I cytoskeletal 28** | **Krt28** | **7.89** | **6.21** |
|  | **B1AQ75** | **Keratin, type I cuticular Ha6** | **Krt36** | **7.78** | **5.80** |
|  | **B2RQC6** | **CAD protein** | **Cad** | **2.04** | **0.38** |
|  | **B2RSH2** | **Guanine nucleotide-binding protein G(i) subunit alpha-1** | **Gnai1** | **1.55** | **3.06** |
|  | **E9PV24** | **Fibrinogen alpha chain** | **Fga** | **3.05** | **5.08** |
|  | **E9PZQ0** | **Ryanodine receptor 1** | **Ryr1** | **5.87** | **3.99** |
|  | **E9Q4Z2** | **Acetyl-CoA carboxylase 2** | **Acacb** | **1.05** | **1.16** |
|  | **O08528** | **Hexokinase-2** | **Hk2** | **3.56** | **4.23** |
|  | **O08529** | **Calpain-2 catalytic subunit** | **Capn2** | **3.71** | **5.30** |
|  | **O08547** | **Vesicle-trafficking protein SEC22b** | **Sec22b** | **2.34** | **3.69** |
|  | **O08788** | **Dynactin subunit 1** | **Dctn1** | **1.16** | **3.75** |
|  | **O08810** | **116 kDa U5 small nuclear ribonucleoprotein component** | **Eftud2** | **1.73** | **3.78** |
|  | **O09061** | **Proteasome subunit beta type-1** | **Psmb1** | **2.34** | **3.13** |
|  | **O35206** | **Collagen alpha-1(XV) chain** | **Col15a1** | **1.55** | **3.63** |
|  | **O35226** | **26S proteasome non-ATPase regulatory subunit 4** | **Psmd4** | **1.05** | **2.49** |
|  | **O35286** | **Putative pre-mRNA-splicing factor ATP-dependent RNA helicase DHX15** | **Dhx15** | **1.05** | **2.95** |
|  | **O35593** | **26S proteasome non-ATPase regulatory subunit 14** | **Psmd14** | **1.27** | **1.77** |
|  | **O35660** | **Glutathione S-transferase Mu 6** | **Gstm6** | **3.55** | **3.36** |
|  | **O35678** | **Monoglyceride lipase** | **Mgll** | **1.05** | **4.24** |
|  | **O54734** | **Dolichyl-diphosphooligosaccharide--protein glycosyltransferase 48 kDa subunit** | **Ddost** | **1.05** | **3.95** |
|  | **O55143** | **Sarcoplasmic/endoplasmic reticulum calcium ATPase 2** | **Atp2a2** | **9.30** | **8.48** |
|  | **O55234** | **Proteasome subunit beta type-5** | **Psmb5** | **1.84** | **2.57** |
|  | **O70250** | **Phosphoglycerate mutase 2** | **Pgam2** | **5.59** | **6.42** |
|  | **O70423** | **Membrane primary amine oxidase** | **Aoc3** | **2.95** | **4.09** |
|  | **O70456** | **14-3-3 protein sigma** | **Sfn** | **5.69** | **6.42** |
|  | **O70503** | **Estradiol 17-beta-dehydrogenase 12** | **Hsd17b12** | **1.05** | **3.25** |
|  | **O88456** | **Calpain small subunit 1** | **Capns1** | **1.05** | **2.35** |
|  | **O88685** | **26S protease regulatory subunit 6A** | **Psmc3** | **1.55** | **3.26** |
|  | **O88712** | **C-terminal-binding protein 1** | **Ctbp1** | **2.63** | **2.04** |
|  | **O88990** | **Alpha-actinin-3** | **Actn3** | **9.04** | **8.40** |
|  | **O89053** | **Coronin-1A** | **Coro1a** | **2.34** | **3.18** |
|  | **P00329** | **Alcohol dehydrogenase 1** | **Adh1** | **2.04** | **2.27** |
|  | **P00405** | **Cytochrome c oxidase subunit 2** | **Mtco2** | **3.21** | **4.35** |
|  | **P00920** | **Carbonic anhydrase 2** | **Ca2** | **2.44** | **1.83** |
|  | **P01027** | **Complement C3** | **C3** | **3.99** | **7.57** |
|  | **P01029** | **Complement C4-B** | **C4b** | **1.04** | **2.85** |
|  | **P01831** | **Thy-1 membrane glycoprotein** | **Thy1** | **2.16** | **3.23** |
|  | **P02535** | **Keratin, type I cytoskeletal 10** | **Krt10** | **9.92** | **9.55** |
|  | **P04104** | **Keratin, type II cytoskeletal 1** | **Krt1** | **9.54** | **9.05** |
|  | **P04117** | **Fatty acid-binding protein, adipocyte** | **Fabp4** | **5.46** | **5.22** |
|  | **P05532** | **Mast/stem cell growth factor receptor Kit** | **Kit** | **1.95** | **1.66** |
|  | **P05784** | **Keratin, type I cytoskeletal 18** | **Krt18** | **7.99** | **6.73** |
|  | **P06801** | **NADP-dependent malic enzyme** | **Me1** | **3.02** | **4.19** |
|  | **P07310** | **Creatine kinase M-type** | **Ckm** | **10.26** | **9.24** |
|  | **P07356** | **Annexin A2** | **Anxa2** | **6.75** | **7.94** |
|  | **P07744** | **Keratin, type II cytoskeletal 4** | **Krt4** | **8.51** | **8.42** |
|  | **P07759** | **Serine protease inhibitor A3K** | **Serpina3k** | **1.04** | **8.03** |
|  | **P08071** | **Lactotransferrin** | **Ltf** | **1.04** | **3.39** |
|  | **P08103** | **Tyrosine-protein kinase HCK** | **Hck** | **1.66** | **0.17** |
|  | **P08121** | **Collagen alpha-1(III) chain** | **Col3a1** | **1.16** | **2.65** |
|  | **P08226** | **Apolipoprotein E** | **Apoe** | **3.61** | **5.90** |
|  | **P08730** | **Keratin, type I cytoskeletal 13** | **Krt13** | **8.76** | **7.88** |
|  | **P08752** | **Guanine nucleotide-binding protein G(i) subunit alpha-2** | **Gnai2** | **2.84** | **3.61** |
|  | **P09055** | **Integrin beta-1** | **Itgb1** | **1.55** | **4.31** |
|  | **P09103** | **Protein disulfide-isomerase** | **P4hb** | **5.24** | **5.88** |
|  | **P09542** | **Myosin light chain 3** | **Myl3** | **6.12** | **4.71** |
|  | **P0C0S6** | **Histone H2A.Z** | **H2afz** | **4.97** | **5.61** |
|  | **P10107** | **Annexin A1** | **Anxa1** | **5.57** | **7.05** |
|  | **P10605** | **Cathepsin B** | **Ctsb** | **1.05** | **5.16** |
|  | **P10649** | **Glutathione S-transferase Mu 1** | **Gstm1** | **4.82** | **5.52** |
|  | **P10854** | **Histone H2B type 1-M** | **Hist1h2bm** | **5.82** | **5.86** |
|  | **P11087** | **Collagen alpha-1(I) chain** | **Col1a1** | **3.49** | **3.56** |
|  | **P11247** | **Myeloperoxidase** | **Mpo** | **1.04** | **3.17** |
|  | **P11679** | **Keratin, type II cytoskeletal 8** | **Krt8** | **8.57** | **8.03** |
|  | **P11688** | **Integrin alpha-5** | **Itga5** | **2.04** | **0.56** |
|  | **P12382** | **ATP-dependent 6-phosphofructokinase, liver type** | **Pfkl** | **4.94** | **5.17** |
|  | **P12970** | **60S ribosomal protein L7a** | **Rpl7a** | **2.71** | **5.24** |
|  | **P13020** | **Gelsolin** | **Gsn** | **3.63** | **6.53** |
|  | **P13541** | **Myosin-3** | **Myh3** | **12.07** | **11.30** |
|  | **P13542** | **Myosin-8** | **Myh8** | **12.51** | **11.96** |
|  | **P14115** | **60S ribosomal protein L27a** | **Rpl27a** | **1.55** | **4.07** |
|  | **P14131** | **40S ribosomal protein S16** | **Rps16** | **3.00** | **4.48** |
|  | **P14206** | **40S ribosomal protein SA** | **Rpsa** | **4.41** | **5.14** |
|  | **P14602** | **Heat shock protein beta-1** | **Hspb1** | **5.81** | **5.54** |
|  | **P15105** | **Glutamine synthetase** | **Glul** | **2.63** | **3.89** |
|  | **P15327** | **Bisphosphoglycerate mutase** | **Bpgm** | **0.94** | **1.17** |
|  | **P15532** | **Nucleoside diphosphate kinase A** | **Nme1** | **2.34** | **4.04** |
|  | **P15626** | **Glutathione S-transferase Mu 2** | **Gstm2** | **4.84** | **5.01** |
|  | **P17426** | **AP-2 complex subunit alpha-1** | **Ap2a1** | **1.94** | **4.47** |
|  | **P17427** | **AP-2 complex subunit alpha-2** | **Ap2a2** | **1.84** | **5.25** |
|  | **P17879** | **Heat shock 70 kDa protein 1B** | **Hspa1b** | **5.50** | **4.77** |
|  | **P18242** | **Cathepsin D** | **Ctsd** | **1.05** | **4.01** |
|  | **P18760** | **Cofilin-1** | **Cfl1** | **3.95** | **4.90** |
|  | **P18872** | **Guanine nucleotide-binding protein G(o) subunit alpha** | **Gnao1** | **1.55** | **2.53** |
|  | **P19001** | **Keratin, type I cytoskeletal 19** | **Krt19** | **8.71** | **7.75** |
|  | **P19096** | **Fatty acid synthase** | **Fasn** | **6.35** | **6.79** |
|  | **P19221** | **Prothrombin** | **F2** | **2.73** | **2.81** |
|  | **P19253** | **60S ribosomal protein L13a** | **Rpl13a** | **1.05** | **3.23** |
|  | **P19324** | **Serpin H1** | **Serpinh1** | **5.12** | **6.26** |
|  | **P19639** | **Glutathione S-transferase Mu 3** | **Gstm3** | **3.00** | **4.45** |
|  | **P19783** | **Cytochrome c oxidase subunit 4 isoform 1, mitochondrial** | **Cox4i1** | **2.34** | **3.02** |
|  | **P20152** | **Vimentin** | **Vim** | **7.84** | **8.75** |
|  | **P20918** | **Plasminogen** | **Plg** | **4.40** | **4.91** |
|  | **P21107** | **Tropomyosin alpha-3 chain** | **Tpm3** | **8.44** | **6.89** |
|  | **P21278** | **Guanine nucleotide-binding protein subunit alpha-11** | **Gna11** | **1.55** | **0.17** |
|  | **P21279** | **Guanine nucleotide-binding protein G(q) subunit alpha** | **Gnaq** | **2.38** | **0.17** |
|  | **P21300** | **Aldose reductase-related protein 1** | **Akr1b7** | **1.04** | **0.44** |
|  | **P23927** | **Alpha-crystallin B chain** | **Cryab** | **4.84** | **5.06** |
|  | **P24270** | **Catalase** | **Cat** | **4.55** | **4.00** |
|  | **P24527** | **Leukotriene A-4 hydrolase** | **Lta4h** | **4.02** | **4.20** |
|  | **P24549** | **Retinal dehydrogenase 1** | **Aldh1a1** | **3.86** | **2.10** |
|  | **P25444** | **40S ribosomal protein S2** | **Rps2** | **5.43** | **5.49** |
|  | **P26039** | **Talin-1** | **Tln1** | **4.67** | **6.37** |
|  | **P26041** | **Moesin** | **Msn** | **6.11** | **6.81** |
|  | **P26231** | **Catenin alpha-1** | **Ctnna1** | **3.72** | **5.33** |
|  | **P26645** | **Myristoylated alanine-rich C-kinase substrate** | **Marcks** | **1.38** | **3.34** |
|  | **P27600** | **Guanine nucleotide-binding protein subunit alpha-12** | **Gna12** | **1.55** | **1.86** |
|  | **P27601** | **Guanine nucleotide-binding protein subunit alpha-13** | **Gna13** | **1.55** | **1.86** |
|  | **P27661** | **Histone H2AX** | **H2afx** | **4.91** | **5.66** |
|  | **P28076** | **Proteasome subunit beta type-9** | **Psmb9** | **1.05** | **1.67** |
|  | **P28271** | **Cytoplasmic aconitate hydratase** | **Aco1** | **4.27** | **4.24** |
|  | **P28474** | **Alcohol dehydrogenase class-3** | **Adh5** | **2.04** | **1.66** |
|  | **P28650** | **Adenylosuccinate synthetase isozyme 1** | **Adssl1** | **4.95** | **4.58** |
|  | **P30677** | **Guanine nucleotide-binding protein subunit alpha-14** | **Gna14** | **1.71** | **0.17** |
|  | **P31001** | **Desmin** | **Des** | **7.37** | **7.56** |
|  | **P32020** | **Non-specific lipid-transfer protein** | **Scp2** | **1.27** | **2.49** |
|  | **P32261** | **Antithrombin-III** | **Serpinc1** | **1.04** | **2.38** |
|  | **P35276** | **Ras-related protein Rab-3D** | **Rab3d** | **1.55** | **3.15** |
|  | **P35278** | **Ras-related protein Rab-5C** | **Rab5c** | **2.05** | **3.95** |
|  | **P35492** | **Histidine ammonia-lyase** | **Hal** | **1.55** | **4.82** |
|  | **P35564** | **Calnexin** | **Canx** | **3.13** | **5.16** |
|  | **P35979** | **60S ribosomal protein L12** | **Rpl12** | **2.55** | **4.14** |
|  | **P35980** | **60S ribosomal protein L18** | **Rpl18** | **3.50** | **4.63** |
|  | **P38060** | **Hydroxymethylglutaryl-CoA lyase, mitochondrial** | **Hmgcl** | **1.44** | **1.97** |
|  | **P39053** | **Dynamin-1** | **Dnm1** | **3.32** | **3.27** |
|  | **P39054** | **Dynamin-2** | **Dnm2** | **1.73** | **3.67** |
|  | **P39061** | **Collagen alpha-1(XVIII) chain** | **Col18a1** | **3.04** | **3.92** |
|  | **P39688** | **Tyrosine-protein kinase Fyn** | **Fyn** | **1.66** | **0.17** |
|  | **P40142** | **Transketolase** | **Tkt** | **3.37** | **5.74** |
|  | **P41105** | **60S ribosomal protein L28** | **Rpl28** | **2.82** | **3.50** |
|  | **P41216** | **Long-chain-fatty-acid--CoA ligase 1** | **Acsl1** | **5.56** | **6.05** |
|  | **P45377** | **Aldose reductase-related protein 2** | **Akr1b8** | **1.04** | **0.97** |
|  | **P45952** | **Medium-chain specific acyl-CoA dehydrogenase, mitochondrial** | **Acadm** | **3.63** | **3.68** |
|  | **P46412** | **Glutathione peroxidase 3** | **Gpx3** | **1.84** | **2.86** |
|  | **P46425** | **Glutathione S-transferase P 2** | **Gstp2** | **2.05** | **3.12** |
|  | **P46460** | **Vesicle-fusing ATPase** | **Nsf** | **1.66** | **2.93** |
|  | **P46638** | **Ras-related protein Rab-11B** | **Rab11b** | **2.84** | **4.00** |
|  | **P46935** | **E3 ubiquitin-protein ligase NEDD4** | **Nedd4** | **3.34** | **4.64** |
|  | **P46978** | **Dolichyl-diphosphooligosaccharide--protein glycosyltransferase subunit STT3A** | **Stt3a** | **1.16** | **1.86** |
|  | **P47740** | **Fatty aldehyde dehydrogenase** | **Aldh3a2** | **2.04** | **1.66** |
|  | **P47791** | **Glutathione reductase, mitochondrial** | **Gsr** | **1.95** | **1.16** |
|  | **P47856** | **Glutamine--fructose-6-phosphate aminotransferase [isomerizing] 1** | **Gfpt1** | **1.04** | **1.10** |
|  | **P47857** | **ATP-dependent 6-phosphofructokinase, muscle type** | **Pfkm** | **6.87** | **6.87** |
|  | **P47915** | **60S ribosomal protein L29** | **Rpl29** | **2.63** | **4.38** |
|  | **P48036** | **Annexin A5** | **Anxa5** | **5.82** | **6.38** |
|  | **P48453** | **Serine/threonine-protein phosphatase 2B catalytic subunit beta isoform** | **Ppp3cb** | **2.74** | **3.76** |
|  | **P48678** | **Prelamin-A/C** | **Lmna** | **7.52** | **8.42** |
|  | **P48722** | **Heat shock 70 kDa protein 4L** | **Hspa4l** | **1.95** | **3.76** |
|  | **P48774** | **Glutathione S-transferase Mu 5** | **Gstm5** | **1.55** | **3.72** |
|  | **P48962** | **ADP/ATP translocase 1** | **Slc25a4** | **7.20** | **7.40** |
|  | **P49182** | **Heparin cofactor 2** | **Serpind1** | **1.04** | **2.27** |
|  | **P49312** | **Heterogeneous nuclear ribonucleoprotein A1** | **Hnrnpa1** | **0.94** | **4.22** |
|  | **P50247** | **Adenosylhomocysteinase** | **Ahcy** | **5.09** | **5.36** |
|  | **P50431** | **Serine hydroxymethyltransferase, cytosolic** | **Shmt1** | **1.04** | **0.77** |
|  | **P50446** | **Keratin, type II cytoskeletal 6A** | **Krt6a** | **8.64** | **9.47** |
|  | **P50516** | **V-type proton ATPase catalytic subunit A** | **Atp6v1a** | **2.84** | **3.35** |
|  | **P50518** | **V-type proton ATPase subunit E 1** | **Atp6v1e1** | **2.04** | **2.69** |
|  | **P50544** | **Very long-chain specific acyl-CoA dehydrogenase, mitochondrial** | **Acadvl** | **1.55** | **4.22** |
|  | **P51660** | **Peroxisomal multifunctional enzyme type 2** | **Hsd17b4** | **1.66** | **4.57** |
|  | **P51667** | **Myosin regulatory light chain 2, ventricular/cardiac muscle isoform** | **Myl2** | **3.21** | **1.44** |
|  | **P51881** | **ADP/ATP translocase 2** | **Slc25a5** | **6.11** | **7.05** |
|  | **P54775** | **26S protease regulatory subunit 6B** | **Psmc4** | **2.34** | **3.25** |
|  | **P55258** | **Ras-related protein Rab-8A** | **Rab8a** | **2.21** | **4.03** |
|  | **P57746** | **V-type proton ATPase subunit D** | **Atp6v1d** | **1.04** | **1.10** |
|  | **P57784** | **U2 small nuclear ribonucleoprotein A~** | **Snrpa1** | **2.04** | **0.44** |
|  | **P58771** | **Tropomyosin alpha-1 chain** | **Tpm1** | **9.30** | **8.38** |
|  | **P58774** | **Tropomyosin beta chain** | **Tpm2** | **9.11** | **8.14** |
|  | **P59999** | **Actin-related protein 2/3 complex subunit 4** | **Arpc4** | **3.21** | **3.73** |
|  | **P60764** | **Ras-related C3 botulinum toxin substrate 3** | **Rac3** | **0.83** | **2.49** |
|  | **P60766** | **Cell division control protein 42 homolog** | **Cdc42** | **2.34** | **3.91** |
|  | **P60867** | **40S ribosomal protein S20** | **Rps20** | **1.27** | **2.07** |
|  | **P61021** | **Ras-related protein Rab-5B** | **Rab5b** | **2.63** | **3.17** |
|  | **P61089** | **Ubiquitin-conjugating enzyme E2 N** | **Ube2n** | **1.84** | **2.60** |
|  | **P61255** | **60S ribosomal protein L26** | **Rpl26** | **2.34** | **3.98** |
|  | **P61358** | **60S ribosomal protein L27** | **Rpl27** | **2.66** | **2.88** |
|  | **P61620** | **Protein transport protein Sec61 subunit alpha isoform 1** | **Sec61a1** | **2.04** | **0.77** |
|  | **P61979** | **Heterogeneous nuclear ribonucleoprotein K** | **Hnrnpk** | **3.29** | **4.72** |
|  | **P62082** | **40S ribosomal protein S7** | **Rps7** | **2.05** | **4.34** |
|  | **P62196** | **26S protease regulatory subunit 8** | **Psmc5** | **2.32** | **2.90** |
|  | **P62242** | **40S ribosomal protein S8** | **Rps8** | **3.34** | **4.63** |
|  | **P62264** | **40S ribosomal protein S14** | **Rps14** | **1.84** | **3.25** |
|  | **P62270** | **40S ribosomal protein S18** | **Rps18** | **2.77** | **4.40** |
|  | **P62281** | **40S ribosomal protein S11** | **Rps11** | **2.58** | **3.15** |
|  | **P62301** | **40S ribosomal protein S13** | **Rps13** | **1.55** | **3.35** |
|  | **P62315** | **Small nuclear ribonucleoprotein Sm D1** | **Snrpd1** | **0.83** | **2.76** |
|  | **P62317** | **Small nuclear ribonucleoprotein Sm D2** | **Snrpd2** | **1.27** | **2.30** |
|  | **P62715** | **Serine/threonine-protein phosphatase 2A catalytic subunit beta isoform** | **Ppp2cb** | **1.55** | **4.83** |
|  | **P62717** | **60S ribosomal protein L18a** | **Rpl18a** | **3.54** | **3.31** |
|  | **P62754** | **40S ribosomal protein S6** | **Rps6** | **4.98** | **4.85** |
|  | **P62814** | **V-type proton ATPase subunit B, brain isoform** | **Atp6v1b2** | **2.23** | **1.77** |
|  | **P62830** | **60S ribosomal protein L23** | **Rpl23** | **1.55** | **3.76** |
|  | **P62835** | **Ras-related protein Rap-1A** | **Rap1a** | **2.27** | **4.63** |
|  | **P62855** | **40S ribosomal protein S26** | **Rps26** | **0.94** | **3.77** |
|  | **P62900** | **60S ribosomal protein L31** | **Rpl31** | **2.05** | **2.83** |
|  | **P62908** | **40S ribosomal protein S3** | **Rps3** | **5.00** | **6.31** |
|  | **P62911** | **60S ribosomal protein L32** | **Rpl32** | **3.85** | **0.77** |
|  | **P62918** | **60S ribosomal protein L8** | **Rpl8** | **2.34** | **4.64** |
|  | **P62962** | **Profilin-1** | **Pfn1** | **3.93** | **5.55** |
|  | **P62983** | **Ubiquitin-40S ribosomal protein S27a** | **Rps27a** | **2.23** | **4.70** |
|  | **P62984** | **Ubiquitin-60S ribosomal protein L40** | **Uba52** | **2.23** | **5.02** |
|  | **P63001** | **Ras-related C3 botulinum toxin substrate 1** | **Rac1** | **2.34** | **2.80** |
|  | **P63024** | **Vesicle-associated membrane protein 3** | **Vamp3** | **1.05** | **1.63** |
|  | **P63044** | **Vesicle-associated membrane protein 2** | **Vamp2** | **1.05** | **1.10** |
|  | **P63085** | **Mitogen-activated protein kinase 1** | **Mapk1** | **3.95** | **3.61** |
|  | **P63094** | **Guanine nucleotide-binding protein G(s) subunit alpha isoforms short** | **Gnas** | **1.55** | **2.82** |
|  | **P63101** | **14-3-3 protein zeta/delta** | **Ywhaz** | **5.88** | **6.81** |
|  | **P63276** | **40S ribosomal protein S17** | **Rps17** | **1.55** | **3.32** |
|  | **P63325** | **40S ribosomal protein S10** | **Rps10** | **1.83** | **4.53** |
|  | **P63328** | **Serine/threonine-protein phosphatase 2B catalytic subunit alpha isoform** | **Ppp3ca** | **3.32** | **3.87** |
|  | **P63330** | **Serine/threonine-protein phosphatase 2A catalytic subunit alpha isoform** | **Ppp2ca** | **2.05** | **4.09** |
|  | **P67984** | **60S ribosomal protein L22** | **Rpl22** | **1.55** | **3.22** |
|  | **P68033** | **Actin, alpha cardiac muscle 1** | **Actc1** | **10.84** | **10.45** |
|  | **P70124** | **Serpin B5** | **Serpinb5** | **4.55** | **4.26** |
|  | **P70195** | **Proteasome subunit beta type-7** | **Psmb7** | **0.94** | **2.76** |
|  | **P70335** | **Rho-associated protein kinase 1** | **Rock1** | **1.04** | **1.44** |
|  | **P70336** | **Rho-associated protein kinase 2** | **Rock2** | **0.83** | **1.97** |
|  | **P70460** | **Vasodilator-stimulated phosphoprotein** | **Vasp** | **1.04** | **1.44** |
|  | **P70691** | **UDP-glucuronosyltransferase 1-2** | **Ugt1a2** | **3.85** | **2.16** |
|  | **P70696** | **Histone H2B type 1-A** | **Hist1h2ba** | **4.21** | **5.59** |
|  | **P70698** | **CTP synthase 1** | **Ctps1** | **2.27** | **2.69** |
|  | **P84091** | **AP-2 complex subunit mu** | **Ap2m1** | **1.05** | **3.08** |
|  | **P84104** | **Serine/arginine-rich splicing factor 3** | **Srsf3** | **1.44** | **2.53** |
|  | **P97290** | **Plasma protease C1 inhibitor** | **Serping1** | **1.66** | **3.27** |
|  | **P97298** | **Pigment epithelium-derived factor** | **Serpinf1** | **2.32** | **3.53** |
|  | **P97324** | **Glucose-6-phosphate 1-dehydrogenase 2** | **G6pd2** | **2.38** | **2.69** |
|  | **P97351** | **40S ribosomal protein S3a** | **Rps3a** | **4.89** | **5.47** |
|  | **P97372** | **Proteasome activator complex subunit 2** | **Psme2** | **1.16** | **2.81** |
|  | **P97379** | **Ras GTPase-activating protein-binding protein 2** | **G3bp2** | **1.27** | **1.30** |
|  | **P97429** | **Annexin A4** | **Anxa4** | **4.31** | **5.33** |
|  | **P97449** | **Aminopeptidase N** | **Anpep** | **4.37** | **5.29** |
|  | **P97457** | **Myosin regulatory light chain 2, skeletal muscle isoform** | **Mylpf** | **7.48** | **7.28** |
|  | **P97461** | **40S ribosomal protein S5** | **Rps5** | **1.16** | **3.04** |
|  | **P97742** | **Carnitine O-palmitoyltransferase 1, liver isoform** | **Cpt1a** | **1.95** | **2.44** |
|  | **P97861** | **Keratin, type II cuticular Hb6** | **Krt86** | **6.03** | **5.82** |
|  | **P97927** | **Laminin subunit alpha-4** | **Lama4** | **4.37** | **1.97** |
|  | **P99026** | **Proteasome subunit beta type-4** | **Psmb4** | **1.55** | **2.81** |
|  | **P99027** | **60S acidic ribosomal protein P2** | **Rplp2** | **0.83** | **1.54** |
|  | **Q00519** | **Xanthine dehydrogenase/oxidase** | **Xdh** | **4.32** | **5.65** |
|  | **Q00612** | **Glucose-6-phosphate 1-dehydrogenase X** | **G6pdx** | **3.30** | **3.51** |
|  | **Q01149** | **Collagen alpha-2(I) chain** | **Col1a2** | **3.28** | **4.07** |
|  | **Q01405** | **Protein transport protein Sec23A** | **Sec23a** | **0.94** | **3.53** |
|  | **Q01768** | **Nucleoside diphosphate kinase B** | **Nme2** | **3.21** | **4.45** |
|  | **Q02053** | **Ubiquitin-like modifier-activating enzyme 1** | **Uba1** | **4.53** | **5.93** |
|  | **Q02248** | **Catenin beta-1** | **Ctnnb1** | **4.88** | **4.99** |
|  | **Q02566** | **Myosin-6** | **Myh6** | **11.51** | **10.83** |
|  | **Q02788** | **Collagen alpha-2(VI) chain** | **Col6a2** | **4.92** | **2.75** |
|  | **Q04447** | **Creatine kinase B-type** | **Ckb** | **4.56** | **4.72** |
|  | **Q04736** | **Tyrosine-protein kinase Yes** | **Yes1** | **1.66** | **0.17** |
|  | **Q04857** | **Collagen alpha-1(VI) chain** | **Col6a1** | **2.34** | **4.03** |
|  | **Q05144** | **Ras-related C3 botulinum toxin substrate 2** | **Rac2** | **1.55** | **2.70** |
|  | **Q05793** | **Basement membrane-specific heparan sulfate proteoglycan core protein** | **Hspg2** | **3.00** | **5.82** |
|  | **Q07076** | **Annexin A7** | **Anxa7** | **2.05** | **4.32** |
|  | **Q07417** | **Short-chain specific acyl-CoA dehydrogenase, mitochondrial** | **Acads** | **2.44** | **3.11** |
|  | **Q0VBK2** | **Keratin, type II cytoskeletal 80** | **Krt80** | **1.44** | **4.05** |
|  | **Q3THE2** | **Myosin regulatory light chain 12B** | **Myl12b** | **3.45** | **4.27** |
|  | **Q3THW5** | **Histone H2A.V** | **H2afv** | **5.08** | **2.86** |
|  | **Q3TRM8** | **Hexokinase-3** | **Hk3** | **0.83** | **1.98** |
|  | **Q3TTY5** | **Keratin, type II cytoskeletal 2 epidermal** | **Krt2** | **8.73** | **8.26** |
|  | **Q3TXS7** | **26S proteasome non-ATPase regulatory subunit 1** | **Psmd1** | **2.45** | **4.29** |
|  | **Q3TZ89** | **Protein transport protein Sec31B** | **Sec31b** | **1.73** | **0.56** |
|  | **Q3UPL0** | **Protein transport protein Sec31A** | **Sec31a** | **3.37** | **5.01** |
|  | **Q3UQ44** | **Ras GTPase-activating-like protein IQGAP2** | **Iqgap2** | **1.95** | **3.13** |
|  | **Q3UV17** | **Keratin, type II cytoskeletal 2 oral** | **Krt76** | **8.97** | **8.74** |
|  | **Q497I4** | **Keratin, type I cuticular Ha5** | **Krt35** | **7.89** | **6.00** |
|  | **Q5SQX6** | **Cytoplasmic FMR1-interacting protein 2** | **Cyfip2** | **3.63** | **2.17** |
|  | **Q5SUR0** | **Phosphoribosylformylglycinamidine synthase** | **Pfas** | **1.04** | **0.97** |
|  | **Q5SWU9** | **Acetyl-CoA carboxylase 1** | **Acaca** | **2.66** | **2.04** |
|  | **Q5SX39** | **Myosin-4** | **Myh4** | **13.49** | **12.77** |
|  | **Q5SX40** | **Myosin-1** | **Myh1** | **13.03** | **12.57** |
|  | **Q60597** | **2-oxoglutarate dehydrogenase, mitochondrial** | **Ogdh** | **6.47** | **5.66** |
|  | **Q60692** | **Proteasome subunit beta type-6** | **Psmb6** | **1.27** | **2.07** |
|  | **Q60847** | **Collagen alpha-1(XII) chain** | **Col12a1** | **3.00** | **2.54** |
|  | **Q60854** | **Serpin B6** | **Serpinb6** | **3.48** | **5.91** |
|  | **Q60930** | **Voltage-dependent anion-selective channel protein 2** | **Vdac2** | **3.02** | **4.16** |
|  | **Q60931** | **Voltage-dependent anion-selective channel protein 3** | **Vdac3** | **3.82** | **5.01** |
|  | **Q60932** | **Voltage-dependent anion-selective channel protein 1** | **Vdac1** | **4.59** | **5.90** |
|  | **Q61035** | **Histidine--tRNA ligase, cytoplasmic** | **Hars** | **2.86** | **1.30** |
|  | **Q61129** | **Complement factor I** | **Cfi** | **1.05** | **0.17** |
|  | **Q61205** | **Platelet-activating factor acetylhydrolase IB subunit gamma** | **Pafah1b3** | **1.44** | **1.17** |
|  | **Q61292** | **Laminin subunit beta-2** | **Lamb2** | **3.35** | **2.85** |
|  | **Q61301** | **Catenin alpha-2** | **Ctnna2** | **2.82** | **3.59** |
|  | **Q61414** | **Keratin, type I cytoskeletal 15** | **Krt15** | **9.68** | **9.43** |
|  | **Q61425** | **Hydroxyacyl-coenzyme A dehydrogenase, mitochondrial** | **Hadh** | **2.71** | **4.43** |
|  | **Q61495** | **Desmoglein-1-alpha** | **Dsg1a** | **1.84** | **2.97** |
|  | **Q61543** | **Golgi apparatus protein 1** | **Glg1** | **1.04** | **3.16** |
|  | **Q61595** | **Kinectin** | **Ktn1** | **0.94** | **3.12** |
|  | **Q61696** | **Heat shock 70 kDa protein 1A** | **Hspa1a** | **5.60** | **6.83** |
|  | **Q61699** | **Heat shock protein 105 kDa** | **Hsph1** | **5.74** | **4.20** |
|  | **Q61753** | **D-3-phosphoglycerate dehydrogenase** | **Phgdh** | **1.16** | **3.01** |
|  | **Q61754** | **Kallikrein 1-related peptidase b24** | **Klk1b24** | **3.37** | **1.56** |
|  | **Q61765** | **Keratin, type I cuticular Ha1** | **Krt31** | **8.16** | **6.16** |
|  | **Q61781** | **Keratin, type I cytoskeletal 14** | **Krt14** | **9.79** | **9.54** |
|  | **Q61830** | **Macrophage mannose receptor 1** | **Mrc1** | **1.55** | **5.04** |
|  | **Q61838** | **Alpha-2-macroglobulin** | **A2m** | **2.63** | **6.93** |
|  | **Q61897** | **Keratin, type I cuticular Ha3-II** | **Krt33b** | **7.93** | **6.12** |
|  | **Q62093** | **Serine/arginine-rich splicing factor 2** | **Srsf2** | **1.66** | **2.41** |
|  | **Q62167** | **ATP-dependent RNA helicase DDX3X** | **Ddx3x** | **1.55** | **4.92** |
|  | **Q62168** | **Keratin, type I cuticular Ha2** | **Krt32** | **7.74** | **5.84** |
|  | **Q62186** | **Translocon-associated protein subunit delta** | **Ssr4** | **1.05** | **2.07** |
|  | **Q62189** | **U1 small nuclear ribonucleoprotein A** | **Snrpa** | **1.16** | **2.49** |
|  | **Q62452** | **UDP-glucuronosyltransferase 1-9** | **Ugt1a9** | **3.85** | **2.56** |
|  | **Q62523** | **Zyxin** | **Zyx** | **1.71** | **3.23** |
|  | **Q63886** | **UDP-glucuronosyltransferase 1-1** | **Ugt1a1** | **3.85** | **2.53** |
|  | **Q64133** | **Amine oxidase [flavin-containing] A** | **Maoa** | **2.04** | **2.16** |
|  | **Q64442** | **Sorbitol dehydrogenase** | **Sord** | **1.66** | **0.38** |
|  | **Q64449** | **C-type mannose receptor 2** | **Mrc2** | **1.27** | **2.00** |
|  | **Q64475** | **Histone H2B type 1-B** | **Hist1h2bb** | **5.60** | **7.80** |
|  | **Q64518** | **Sarcoplasmic/endoplasmic reticulum calcium ATPase 3** | **Atp2a3** | **7.82** | **6.99** |
|  | **Q64522** | **Histone H2A type 2-B** | **Hist2h2ab** | **4.22** | **5.87** |
|  | **Q64525** | **Histone H2B type 2-B** | **Hist2h2bb** | **5.79** | **7.65** |
|  | **Q64737** | **Trifunctional purine biosynthetic protein adenosine-3** | **Gart** | **1.04** | **2.74** |
|  | **Q68FD5** | **Clathrin heavy chain 1** | **Cltc** | **5.66** | **7.28** |
|  | **Q6GQT1** | **Alpha-2-macroglobulin-P** | **A2m** | **1.04** | **2.71** |
|  | **Q6IFX2** | **Keratin, type I cytoskeletal 42** | **Krt42** | **8.95** | **8.16** |
|  | **Q6IFX3** | **Keratin, type I cytoskeletal 40** | **Krt40** | **7.79** | **5.84** |
|  | **Q6IFZ6** | **Keratin, type II cytoskeletal 1b** | **Krt77** | **8.72** | **9.39** |
|  | **Q6IFZ9** | **Keratin, type II cytoskeletal 74** | **Krt74** | **8.19** | **7.51** |
|  | **Q6IME9** | **Keratin, type II cytoskeletal 72** | **Krt72** | **8.15** | **7.81** |
|  | **Q6IMF0** | **Keratin, type II cuticular Hb3** | **Krt83** | **5.92** | **5.61** |
|  | **Q6IRU2** | **Tropomyosin alpha-4 chain** | **Tpm4** | **5.57** | **4.83** |
|  | **Q6NXH9** | **Keratin, type II cytoskeletal 73** | **Krt73** | **8.49** | **8.14** |
|  | **Q6P4P1** | **Serine protease inhibitor A3A** | **Serpina3a** | **1.04** | **0.36** |
|  | **Q6P5E4** | **UDP-glucose:glycoprotein glucosyltransferase 1** | **Uggt1** | **1.04** | **3.56** |
|  | **Q6P8J7** | **Creatine kinase S-type, mitochondrial** | **Ckmt2** | **3.35** | **3.08** |
|  | **Q6PDL0** | **Cytoplasmic dynein 1 light intermediate chain 2** | **Dync1li2** | **1.16** | **1.44** |
|  | **Q6PDM2** | **Serine/arginine-rich splicing factor 1** | **Srsf1** | **3.89** | **3.78** |
|  | **Q6PIE5** | **Sodium/potassium-transporting ATPase subunit alpha-2** | **Atp1a2** | **5.30** | **5.82** |
|  | **Q6R0H7** | **Guanine nucleotide-binding protein G(s) subunit alpha isoforms XLas** | **Gnas** | **1.55** | **2.07** |
|  | **Q6ZQM8** | **UDP-glucuronosyltransferase 1-7C** | **Ugt1a7c** | **3.85** | **2.32** |
|  | **Q6ZWV3** | **60S ribosomal protein L10** | **Rpl10** | **3.32** | **3.63** |
|  | **Q6ZWV7** | **60S ribosomal protein L35** | **Rpl35** | **2.45** | **2.81** |
|  | **Q6ZWX6** | **Eukaryotic translation initiation factor 2 subunit 1** | **Eif2s1** | **2.66** | **2.74** |
|  | **Q71LX4** | **Talin-2** | **Tln2** | **4.48** | **3.46** |
|  | **Q76MZ3** | **Serine/threonine-protein phosphatase 2A 65 kDa regulatory subunit A alpha isoform** | **Ppp2r1a** | **4.81** | **5.07** |
|  | **Q7TMB8** | **Cytoplasmic FMR1-interacting protein 1** | **Cyfip1** | **4.04** | **2.74** |
|  | **Q7TSF0** | **Desmoglein-1-gamma** | **Dsg1c** | **1.27** | **2.63** |
|  | **Q7TSF1** | **Desmoglein-1-beta** | **Dsg1b** | **1.84** | **2.97** |
|  | **Q80UM7** | **Mannosyl-oligosaccharide glucosidase** | **Mogs** | **1.16** | **1.16** |
|  | **Q80VQ0** | **Aldehyde dehydrogenase family 3 member B1** | **Aldh3b1** | **4.21** | **1.54** |
|  | **Q80W21** | **Glutathione S-transferase Mu 7** | **Gstm7** | **4.84** | **4.27** |
|  | **Q80X19** | **Collagen alpha-1(XIV) chain** | **Col14a1** | **5.39** | **6.63** |
|  | **Q8BG32** | **26S proteasome non-ATPase regulatory subunit 11** | **Psmd11** | **3.63** | **2.30** |
|  | **Q8BGZ7** | **Keratin, type II cytoskeletal 75** | **Krt75** | **9.30** | **9.45** |
|  | **Q8BH43** | **Wiskott-Aldrich syndrome protein family member 2** | **Wasf2** | **1.27** | **1.49** |
|  | **Q8BH61** | **Coagulation factor XIII A chain** | **F13a1** | **2.38** | **6.31** |
|  | **Q8BH64** | **EH domain-containing protein 2** | **Ehd2** | **5.48** | **4.38** |
|  | **Q8BH95** | **Enoyl-CoA hydratase, mitochondrial** | **Echs1** | **0.83** | **3.42** |
|  | **Q8BKZ9** | **Pyruvate dehydrogenase protein X component, mitochondrial** | **Pdhx** | **2.16** | **2.54** |
|  | **Q8BL97** | **Serine/arginine-rich splicing factor 7** | **Srsf7** | **1.94** | **3.23** |
|  | **Q8BMF4** | **Dihydrolipoyllysine-residue acetyltransferase component of pyruvate dehydrogenase complex, mitochondrial** | **Dlat** | **3.80** | **4.72** |
|  | **Q8BMJ2** | **Leucine--tRNA ligase, cytoplasmic** | **Lars** | **1.04** | **3.27** |
|  | **Q8BMK4** | **Cytoskeleton-associated protein 4** | **Ckap4** | **5.17** | **5.09** |
|  | **Q8BMS1** | **Trifunctional enzyme subunit alpha, mitochondrial** | **Hadha** | **6.33** | **6.74** |
|  | **Q8BVE3** | **V-type proton ATPase subunit H** | **Atp6v1h** | **1.16** | **0.38** |
|  | **Q8BWT1** | **3-ketoacyl-CoA thiolase, mitochondrial** | **Acaa2** | **4.04** | **5.39** |
|  | **Q8CCX5** | **Keratin-like protein KRT222** | **Krt222** | **5.02** | **4.67** |
|  | **Q8CFI7** | **DNA-directed RNA polymerase II subunit RPB2** | **Polr2b** | **1.27** | **2.17** |
|  | **Q8CGC7** | **Bifunctional glutamate/proline--tRNA ligase** | **Eprs** | **2.10** | **5.24** |
|  | **Q8CGP0** | **Histone H2B type 3-B** | **Hist3h2bb** | **5.56** | **7.79** |
|  | **Q8CGP1** | **Histone H2B type 1-K** | **Hist1h2bk** | **6.14** | **7.72** |
|  | **Q8CGP2** | **Histone H2B type 1-P** | **Hist1h2bp** | **5.82** | **7.80** |
|  | **Q8CHP8** | **Phosphoglycolate phosphatase** | **Pgp** | **1.55** | **2.04** |
|  | **Q8CI94** | **Glycogen phosphorylase, brain form** | **Pygb** | **6.13** | **6.48** |
|  | **Q8JZR0** | **Long-chain-fatty-acid--CoA ligase 5** | **Acsl5** | **3.13** | **4.19** |
|  | **Q8K0E8** | **Fibrinogen beta chain** | **Fgb** | **3.97** | **5.91** |
|  | **Q8K0Y2** | **Keratin, type I cuticular Ha3-I** | **Krt33a** | **4.87** | **3.87** |
|  | **Q8K1M6** | **Dynamin-1-like protein** | **Dnm1l** | **2.34** | **2.87** |
|  | **Q8K2B3** | **Succinate dehydrogenase [ubiquinone] flavoprotein subunit, mitochondrial** | **Sdha** | **4.08** | **5.05** |
|  | **Q8K4Z5** | **Splicing factor 3A subunit 1** | **Sf3a1** | **1.16** | **2.16** |
|  | **Q8R429** | **Sarcoplasmic/endoplasmic reticulum calcium ATPase 1** | **Atp2a1** | **10.61** | **9.91** |
|  | **Q8VCM7** | **Fibrinogen gamma chain** | **Fgg** | **5.51** | **5.97** |
|  | **Q8VCR8** | **Myosin light chain kinase 2, skeletal/cardiac muscle** | **Mylk2** | **3.46** | **3.99** |
|  | **Q8VCW2** | **Keratin, type I cytoskeletal 25** | **Krt25** | **8.62** | **8.25** |
|  | **Q8VDD5** | **Myosin-9** | **Myh9** | **7.49** | **8.48** |
|  | **Q8VDM4** | **26S proteasome non-ATPase regulatory subunit 2** | **Psmd2** | **4.47** | **4.70** |
|  | **Q8VDN2** | **Sodium/potassium-transporting ATPase subunit alpha-1** | **Atp1a1** | **4.83** | **6.04** |
|  | **Q8VED5** | **Keratin, type II cytoskeletal 79** | **Krt79** | **8.58** | **8.78** |
|  | **Q8VHP7** | **Leukocyte elastase inhibitor B** | **Serpinb1b** | **2.66** | **1.49** |
|  | **Q91V92** | **ATP-citrate synthase** | **Acly** | **4.82** | **5.76** |
|  | **Q91VC3** | **Eukaryotic initiation factor 4A-III** | **Eif4a3** | **4.37** | **2.21** |
|  | **Q91VD9** | **NADH-ubiquinone oxidoreductase 75 kDa subunit, mitochondrial** | **Ndufs1** | **3.91** | **5.69** |
|  | **Q91VR2** | **ATP synthase subunit gamma, mitochondrial** | **Atp5c1** | **4.04** | **4.71** |
|  | **Q91W43** | **Glycine dehydrogenase (decarboxylating), mitochondrial** | **Gldc** | **2.63** | **0.77** |
|  | **Q91W90** | **Thioredoxin domain-containing protein 5** | **Txndc5** | **1.94** | **2.76** |
|  | **Q91WD5** | **NADH dehydrogenase [ubiquinone] iron-sulfur protein 2, mitochondrial** | **Ndufs2** | **3.24** | **2.74** |
|  | **Q91WP6** | **Serine protease inhibitor A3N** | **Serpina3n** | **1.04** | **5.15** |
|  | **Q91YQ5** | **Dolichyl-diphosphooligosaccharide--protein glycosyltransferase subunit 1** | **Rpn1** | **2.55** | **4.20** |
|  | **Q91YT0** | **NADH dehydrogenase [ubiquinone] flavoprotein 1, mitochondrial** | **Ndufv1** | **4.13** | **3.85** |
|  | **Q91Z83** | **Myosin-7** | **Myh7** | **11.63** | **10.88** |
|  | **Q91ZJ5** | **UTP--glucose-1-phosphate uridylyltransferase** | **Ugp2** | **3.92** | **3.61** |
|  | **Q91ZX7** | **Prolow-density lipoprotein receptor-related protein 1** | **Lrp1** | **3.21** | **5.94** |
|  | **Q921M3** | **Splicing factor 3B subunit 3** | **Sf3b3** | **1.94** | **4.01** |
|  | **Q922B2** | **Aspartate--tRNA ligase, cytoplasmic** | **Dars** | **1.55** | **3.80** |
|  | **Q922D8** | **C-1-tetrahydrofolate synthase, cytoplasmic** | **Mthfd1** | **2.23** | **3.75** |
|  | **Q922R8** | **Protein disulfide-isomerase A6** | **Pdia6** | **2.55** | **5.05** |
|  | **Q922U2** | **Keratin, type II cytoskeletal 5** | **Krt5** | **9.52** | **10.19** |
|  | **Q93092** | **Transaldolase** | **Taldo1** | **4.45** | **4.77** |
|  | **Q99JI4** | **26S proteasome non-ATPase regulatory subunit 6** | **Psmd6** | **1.27** | **1.66** |
|  | **Q99JI6** | **Ras-related protein Rap-1b** | **Rap1b** | **1.55** | **4.56** |
|  | **Q99JY0** | **Trifunctional enzyme subunit beta, mitochondrial** | **Hadhb** | **5.92** | **5.91** |
|  | **Q99KI0** | **Aconitate hydratase, mitochondrial** | **Aco2** | **6.65** | **7.00** |
|  | **Q99KK9** | **Probable histidine--tRNA ligase, mitochondrial** | **Hars2** | **2.86** | **1.07** |
|  | **Q99KP6** | **Pre-mRNA-processing factor 19** | **Prpf19** | **1.04** | **1.30** |
|  | **Q99L13** | **3-hydroxyisobutyrate dehydrogenase, mitochondrial** | **Hibadh** | **2.58** | **1.92** |
|  | **Q99LC3** | **NADH dehydrogenase [ubiquinone] 1 alpha subcomplex subunit 10, mitochondrial** | **Ndufa10** | **1.05** | **3.20** |
|  | **Q99LX0** | **Protein DJ-1** | **Park7** | **2.84** | **3.63** |
|  | **Q99M73** | **Keratin, type II cuticular Hb4** | **Krt84** | **7.38** | **6.66** |
|  | **Q99M74** | **Keratin, type II cuticular Hb2** | **Krt82** | **5.53** | **4.27** |
|  | **Q99MN1** | **Lysine--tRNA ligase** | **Kars** | **1.05** | **2.57** |
|  | **Q99PL5** | **Ribosome-binding protein 1** | **Rrbp1** | **2.45** | **5.51** |
|  | **Q99PS0** | **Keratin, type I cytoskeletal 23** | **Krt23** | **2.04** | **3.86** |
|  | **Q99PT1** | **Rho GDP-dissociation inhibitor 1** | **Arhgdia** | **2.34** | **3.89** |
|  | **Q9CPW4** | **Actin-related protein 2/3 complex subunit 5** | **Arpc5** | **0.83** | **2.16** |
|  | **Q9CPY7** | **Cytosol aminopeptidase** | **Lap3** | **3.98** | **5.00** |
|  | **Q9CQ19** | **Myosin regulatory light polypeptide 9** | **Myl9** | **2.84** | **3.88** |
|  | **Q9CQ65** | **S-methyl-5~-thioadenosine phosphorylase** | **Mtap** | **2.16** | **2.57** |
|  | **Q9CQA3** | **Succinate dehydrogenase [ubiquinone] iron-sulfur subunit, mitochondrial** | **Sdhb** | **1.84** | **3.35** |
|  | **Q9CQC9** | **GTP-binding protein SAR1b** | **Sar1b** | **1.84** | **3.35** |
|  | **Q9CQD1** | **Ras-related protein Rab-5A** | **Rab5a** | **1.55** | **3.39** |
|  | **Q9CQI7** | **U2 small nuclear ribonucleoprotein B~~** | **Snrpb2** | **1.66** | **1.44** |
|  | **Q9CQQ7** | **ATP synthase F(0) complex subunit B1, mitochondrial** | **Atp5f1** | **3.00** | **3.61** |
|  | **Q9CR57** | **60S ribosomal protein L14** | **Rpl14** | **2.05** | **4.54** |
|  | **Q9CR68** | **Cytochrome b-c1 complex subunit Rieske, mitochondrial** | **Uqcrfs1** | **1.55** | **3.10** |
|  | **Q9CUN6** | **E3 ubiquitin-protein ligase SMURF1** | **Smurf1** | **2.95** | **1.10** |
|  | **Q9CVB6** | **Actin-related protein 2/3 complex subunit 2** | **Arpc2** | **3.41** | **3.78** |
|  | **Q9CWJ9** | **Bifunctional purine biosynthesis protein PURH** | **Atic** | **1.16** | **3.63** |
|  | **Q9CXW4** | **60S ribosomal protein L11** | **Rpl11** | **2.05** | **3.01** |
|  | **Q9CY50** | **Translocon-associated protein subunit alpha** | **Ssr1** | **2.32** | **3.97** |
|  | **Q9CZ13** | **Cytochrome b-c1 complex subunit 1, mitochondrial** | **Uqcrc1** | **2.45** | **4.51** |
|  | **Q9CZM2** | **60S ribosomal protein L15** | **Rpl15** | **4.50** | **4.29** |
|  | **Q9CZT8** | **Ras-related protein Rab-3B** | **Rab3b** | **1.55** | **2.98** |
|  | **Q9CZU6** | **Citrate synthase, mitochondrial** | **Cs** | **4.20** | **4.93** |
|  | **Q9D051** | **Pyruvate dehydrogenase E1 component subunit beta, mitochondrial** | **Pdhb** | **4.04** | **5.02** |
|  | **Q9D0E1** | **Heterogeneous nuclear ribonucleoprotein M** | **Hnrnpm** | **2.05** | **5.53** |
|  | **Q9D0F9** | **Phosphoglucomutase-1** | **Pgm1** | **6.51** | **5.95** |
|  | **Q9D0I9** | **Arginine--tRNA ligase, cytoplasmic** | **Rars** | **3.24** | **2.69** |
|  | **Q9D0K2** | **Succinyl-CoA:3-ketoacid coenzyme A transferase 1, mitochondrial** | **Oxct1** | **4.13** | **4.43** |
|  | **Q9D0M3** | **Cytochrome c1, heme protein, mitochondrial** | **Cyc1** | **3.32** | **3.41** |
|  | **Q9D154** | **Leukocyte elastase inhibitor A** | **Serpinb1a** | **1.04** | **3.20** |
|  | **Q9D1D6** | **Collagen triple helix repeat-containing protein 1** | **Cthrc1** | **0.83** | **2.14** |
|  | **Q9D2U9** | **Histone H2B type 3-A** | **Hist3h2ba** | **5.56** | **6.52** |
|  | **Q9D312** | **Keratin, type I cytoskeletal 20** | **Krt20** | **6.26** | **6.05** |
|  | **Q9D646** | **Keratin, type I cuticular Ha4** | **Krt34** | **5.09** | **4.47** |
|  | **Q9D662** | **Protein transport protein Sec23B** | **Sec23b** | **1.04** | **2.36** |
|  | **Q9D6R2** | **Isocitrate dehydrogenase [NAD] subunit alpha, mitochondrial** | **Idh3a** | **4.44** | **4.68** |
|  | **Q9D819** | **Inorganic pyrophosphatase** | **Ppa1** | **2.66** | **2.78** |
|  | **Q9D8E6** | **60S ribosomal protein L4** | **Rpl4** | **3.00** | **5.47** |
|  | **Q9D8W5** | **26S proteasome non-ATPase regulatory subunit 12** | **Psmd12** | **3.04** | **3.82** |
|  | **Q9DB20** | **ATP synthase subunit O, mitochondrial** | **Atp5o** | **3.00** | **4.52** |
|  | **Q9DB34** | **Charged multivesicular body protein 2a** | **Chmp2a** | **1.27** | **1.10** |
|  | **Q9DBF1** | **Alpha-aminoadipic semialdehyde dehydrogenase** | **Aldh7a1** | **3.04** | **2.36** |
|  | **Q9DBG3** | **AP-2 complex subunit beta** | **Ap2b1** | **1.84** | **4.88** |
|  | **Q9DC51** | **Guanine nucleotide-binding protein G(k) subunit alpha** | **Gnai3** | **1.55** | **3.35** |
|  | **Q9DC70** | **NADH dehydrogenase [ubiquinone] iron-sulfur protein 7, mitochondrial** | **Ndufs7** | **2.34** | **2.41** |
|  | **Q9DCD0** | **6-phosphogluconate dehydrogenase, decarboxylating** | **Pgd** | **4.76** | **4.79** |
|  | **Q9DCT2** | **NADH dehydrogenase [ubiquinone] iron-sulfur protein 3, mitochondrial** | **Ndufs3** | **3.02** | **3.62** |
|  | **Q9DCV7** | **Keratin, type II cytoskeletal 7** | **Krt7** | **7.21** | **7.04** |
|  | **Q9EQ20** | **Methylmalonate-semialdehyde dehydrogenase [acylating], mitochondrial** | **Aldh6a1** | **4.21** | **4.19** |
|  | **Q9ER00** | **Syntaxin-12** | **Stx12** | **3.37** | **3.27** |
|  | **Q9ERE2** | **Keratin, type II cuticular Hb1** | **Krt81** | **6.08** | **5.82** |
|  | **Q9ET01** | **Glycogen phosphorylase, liver form** | **Pygl** | **5.75** | **5.59** |
|  | **Q9JHU4** | **Cytoplasmic dynein 1 heavy chain 1** | **Dync1h1** | **4.96** | **5.24** |
|  | **Q9JHW9** | **Aldehyde dehydrogenase family 1 member A3** | **Aldh1a3** | **1.16** | **0.38** |
|  | **Q9JI91** | **Alpha-actinin-2** | **Actn2** | **8.89** | **9.03** |
|  | **Q9JK48** | **Endophilin-B1** | **Sh3glb1** | **1.66** | **1.66** |
|  | **Q9JK88** | **Serpin I2** | **Serpini2** | **3.63** | **1.36** |
|  | **Q9JKB3** | **Y-box-binding protein 3** | **Ybx3** | **3.11** | **2.41** |
|  | **Q9JKR6** | **Hypoxia up-regulated protein 1** | **Hyou1** | **3.61** | **4.57** |
|  | **Q9JLJ2** | **4-trimethylaminobutyraldehyde dehydrogenase** | **Aldh9a1** | **4.02** | **2.53** |
|  | **Q9JM76** | **Actin-related protein 2/3 complex subunit 3** | **Arpc3** | **1.05** | **3.73** |
|  | **Q9QUI0** | **Transforming protein RhoA** | **Rhoa** | **1.55** | **3.93** |
|  | **Q9QWL7** | **Keratin, type I cytoskeletal 17** | **Krt17** | **9.12** | **8.93** |
|  | **Q9QXS1** | **Plectin** | **Plec** | **8.05** | **9.14** |
|  | **Q9R0H5** | **Keratin, type II cytoskeletal 71** | **Krt71** | **8.52** | **7.99** |
|  | **Q9R0Q6** | **Actin-related protein 2/3 complex subunit 1A** | **Arpc1a** | **1.66** | **0.36** |
|  | **Q9R0Y5** | **Adenylate kinase isoenzyme 1** | **Ak1** | **4.85** | **5.48** |
|  | **Q9R111** | **Guanine deaminase** | **Gda** | **4.71** | **3.44** |
|  | **Q9R1P0** | **Proteasome subunit alpha type-4** | **Psma4** | **2.05** | **3.61** |
|  | **Q9R1P1** | **Proteasome subunit beta type-3** | **Psmb3** | **2.34** | **2.93** |
|  | **Q9R1P4** | **Proteasome subunit alpha type-1** | **Psma1** | **3.90** | **3.47** |
|  | **Q9WTX6** | **Cullin-1** | **Cul1** | **1.16** | **2.17** |
|  | **Q9WU78** | **Programmed cell death 6-interacting protein** | **Pdcd6ip** | **2.84** | **4.75** |
|  | **Q9WUA2** | **Phenylalanine--tRNA ligase beta subunit** | **Farsb** | **1.44** | **2.67** |
|  | **Q9WUA3** | **ATP-dependent 6-phosphofructokinase, platelet type** | **Pfkp** | **3.71** | **5.12** |
|  | **Q9WUB3** | **Glycogen phosphorylase, muscle form** | **Pygm** | **8.26** | **8.49** |
|  | **Q9WUU7** | **Cathepsin Z** | **Ctsz** | **1.04** | **3.15** |
|  | **Q9WV27** | **Sodium/potassium-transporting ATPase subunit alpha-4** | **Atp1a4** | **3.54** | **3.95** |
|  | **Q9WV32** | **Actin-related protein 2/3 complex subunit 1B** | **Arpc1b** | **2.16** | **2.30** |
|  | **Q9WVJ2** | **26S proteasome non-ATPase regulatory subunit 13** | **Psmd13** | **1.16** | **3.07** |
|  | **Q9WVK4** | **EH domain-containing protein 1** | **Ehd1** | **1.44** | **3.30** |
|  | **Q9Z1B7** | **Mitogen-activated protein kinase 13** | **Mapk13** | **2.05** | **0.77** |
|  | **Q9Z1E4** | **Glycogen [starch] synthase, muscle** | **Gys1** | **1.73** | **4.23** |
|  | **Q9Z1N5** | **Spliceosome RNA helicase Ddx39b** | **Ddx39b** | **2.55** | **4.40** |
|  | **Q9Z1Q9** | **Valine--tRNA ligase** | **Vars** | **3.04** | **3.37** |
|  | **Q9Z1T2** | **Thrombospondin-4** | **Thbs4** | **1.04** | **1.17** |
|  | **Q9Z204** | **Heterogeneous nuclear ribonucleoproteins C1/C2** | **Hnrnpc** | **4.45** | **4.03** |
|  | **Q9Z2I8** | **Succinyl-CoA ligase [GDP-forming] subunit beta, mitochondrial** | **Suclg2** | **2.66** | **3.34** |
|  | **Q9Z2K1** | **Keratin, type I cytoskeletal 16** | **Krt16** | **9.44** | **8.82** |
|  | **Q9Z2T6** | **Keratin, type II cuticular Hb5** | **Krt85** | **5.85** | **6.12** |
|  | **Q9Z2U1** | **Proteasome subunit alpha type-5** | **Psma5** | **2.05** | **2.98** |
|  | **Q9Z320** | **Keratin, type I cytoskeletal 27** | **Krt27** | **8.54** | **8.27** |
|  | **Q9Z331** | **Keratin, type II cytoskeletal 6B** | **Krt6b** | **8.58** | **9.33** |
| ***Acomys* Only** | **G5E829** | **Plasma membrane calcium-transporting ATPase 1** | **Atp2b1** | **1.44** |  |
|  | **O35468** | **Protein Wnt-9b** | **Wnt9b** | **1.04** |  |
|  | **O55226** | **Chondroadherin** | **Chad** | **2.05** |  |
|  | **O70145** | **Neutrophil cytosol factor 2** | **Ncf2** | **0.94** |  |
|  | **P10833** | **Ras-related protein R-Ras** | **Rras** | **1.55** |  |
|  | **P11531** | **Dystrophin** | **Dmd** | **3.68** |  |
|  | **P21180** | **Complement C2** | **C2** | **1.27** |  |
|  | **P21812** | **Mast cell protease 4** | **Mcpt4** | **3.04** |  |
|  | **P21845** | **Tryptase beta-2** | **Tpsb2** | **1.04** |  |
|  | **P25911** | **Tyrosine-protein kinase Lyn** | **Lyn** | **1.66** |  |
|  | **P30115** | **Glutathione S-transferase A3** | **Gsta3** | **1.55** |  |
|  | **P30999** | **Catenin delta-1** | **Ctnnd1** | **2.45** |  |
|  | **P31938** | **Dual specificity mitogen-activated protein kinase kinase 1** | **Map2k1** | **1.95** |  |
|  | **P46467** | **Vacuolar protein sorting-associated protein 4B** | **Vps4b** | **1.27** |  |
|  | **P62492** | **Ras-related protein Rab-11A** | **Rab11a** | **2.27** |  |
|  | **P70695** | **Fructose-1,6-bisphosphatase isozyme 2** | **Fbp2** | **4.15** |  |
|  | **P82347** | **Delta-sarcoglycan** | **Sgcd** | **1.55** |  |
|  | **Q05895** | **Thrombospondin-3** | **Thbs3** | **2.04** |  |
|  | **Q2HXL6** | **ER degradation-enhancing alpha-mannosidase-like protein 3** | **Edem3** | **1.27** |  |
|  | **Q2VIS4** | **Filaggrin-2** | **Flg2** | **2.04** |  |
|  | **Q3TWW8** | **Serine/arginine-rich splicing factor 6** | **Srsf6** | **2.63** |  |
|  | **Q3UP87** | **Neutrophil elastase** | **Elane** | **1.27** |  |
|  | **Q61093** | **Cytochrome b-245 heavy chain** | **Cybb** | **1.04** |  |
|  | **Q64291** | **Keratin, type I cytoskeletal 12** | **Krt12** | **3.35** |  |
|  | **Q6PHZ2** | **Calcium/calmodulin-dependent protein kinase type II subunit delta** | **Camk2d** | **2.10** |  |
|  | **Q7SIG6** | **Arf-GAP with SH3 domain, ANK repeat and PH domain-containing protein 2** | **Asap2** | **3.34** |  |
|  | **Q8BPB5** | **EGF-containing fibulin-like extracellular matrix protein 1** | **Efemp1** | **1.04** |  |
|  | **Q8BVI4** | **Dihydropteridine reductase** | **Qdpr** | **0.83** |  |
|  | **Q8CGP5** | **Histone H2A type 1-F** | **Hist1h2af** | **5.40** |  |
|  | **Q91UZ5** | **Inositol monophosphatase 2** | **Impa2** | **1.95** |  |
|  | **Q91Z69** | **SLIT-ROBO Rho GTPase-activating protein 1** | **Srgap1** | **2.27** |  |
|  | **Q9CZ44** | **NSFL1 cofactor p47** | **Nsfl1c** | **1.44** |  |
|  | **Q9CZS1** | **Aldehyde dehydrogenase X, mitochondrial** | **Aldh1b1** | **2.16** |  |
|  | **Q9D7X8** | **Gamma-glutamylcyclotransferase** | **Ggct** | **1.84** |  |
|  | **Q9D883** | **Splicing factor U2AF 35 kDa subunit** | **U2af1** | **1.04** |  |
|  | **Q9DB73** | **NADH-cytochrome b5 reductase 1** | **Cyb5r1** | **1.94** |  |
|  | **Q9QYF1** | **Retinol dehydrogenase 11** | **Rdh11** | **2.66** |  |
|  | **Q9R0K7** | **Plasma membrane calcium-transporting ATPase 2** | **Atp2b2** | **1.04** |  |
|  | **Q9WUD1** | **STIP1 homology and U box-containing protein 1** | **Stub1** | **4.04** |  |
|  | **Q9WV02** | **RNA-binding motif protein, X chromosome** | **Rbmx** | **0.94** |  |
|  | **Q9WVH9** | **Fibulin-5** | **Fbln5** | **2.84** |  |
| ***Mus* Only** | **O08677** | **Kininogen-1** | **Kng1** |  | **2.65** |
|  | **O08808** | **Protein diaphanous homolog 1** | **Diaph1** |  | **2.76** |
|  | **O54950** | **5~-AMP-activated protein kinase subunit gamma-1** | **Prkag1** |  | **0.77** |
|  | **O70475** | **UDP-glucose 6-dehydrogenase** | **Ugdh** |  | **3.06** |
|  | **O88531** | **Palmitoyl-protein thioesterase 1** | **Ppt1** |  | **1.36** |
|  | **O88543** | **COP9 signalosome complex subunit 3** | **Cops3** |  | **1.54** |
|  | **O88947** | **Coagulation factor X** | **F10** |  | **1.56** |
|  | **P00687** | **Alpha-amylase 1** | **Amy1** |  | **1.57** |
|  | **P01898** | **H-2 class I histocompatibility antigen, Q10 alpha chain** | **H2-Q10** |  | **2.16** |
|  | **P03953** | **Complement factor D** | **Cfd** |  | **0.36** |
|  | **P03958** | **Adenosine deaminase** | **Ada** |  | **0.56** |
|  | **P06683** | **Complement component C9** | **C9** |  | **0.38** |
|  | **P06684** | **Complement C5** | **C5** |  | **1.95** |
|  | **P06797** | **Cathepsin L1** | **Ctsl** |  | **1.53** |
|  | **P07758** | **Alpha-1-antitrypsin 1-1** | **Serpina1a** |  | **5.00** |
|  | **P08074** | **Carbonyl reductase [NADPH] 2** | **Cbr2** |  | **4.46** |
|  | **P09470** | **Angiotensin-converting enzyme** | **Ace** |  | **1.88** |
|  | **P09581** | **Macrophage colony-stimulating factor 1 receptor** | **Csf1r** |  | **0.38** |
|  | **P09813** | **Apolipoprotein A-II** | **Apoa2** |  | **0.88** |
|  | **P11404** | **Fatty acid-binding protein, heart** | **Fabp3** |  | **3.07** |
|  | **P11835** | **Integrin beta-2** | **Itgb2** |  | **2.36** |
|  | **P12032** | **Metalloproteinase inhibitor 1** | **Timp1** |  | **0.58** |
|  | **P12388** | **Plasminogen activator inhibitor 2, macrophage** | **Serpinb2** |  | **2.98** |
|  | **P14069** | **Protein S100-A6** | **S100a6** |  | **2.10** |
|  | **P14106** | **Complement C1q subcomponent subunit B** | **C1qb** |  | **0.77** |
|  | **P15379** | **CD44 antigen** | **Cd44** |  | **1.95** |
|  | **P16460** | **Argininosuccinate synthase** | **Ass1** |  | **1.17** |
|  | **P22599** | **Alpha-1-antitrypsin 1-2** | **Serpina1b** |  | **7.10** |
|  | **P24547** | **Inosine-5~-monophosphate dehydrogenase 2** | **Impdh2** |  | **0.97** |
|  | **P24668** | **Cation-dependent mannose-6-phosphate receptor** | **M6pr** |  | **0.17** |
|  | **P26262** | **Plasma kallikrein** | **Klkb1** |  | **2.56** |
|  | **P27046** | **Alpha-mannosidase 2** | **Man2a1** |  | **0.17** |
|  | **P27612** | **Phospholipase A-2-activating protein** | **Plaa** |  | **1.77** |
|  | **P28867** | **Protein kinase C delta type** | **Prkcd** |  | **0.36** |
|  | **P29351** | **Tyrosine-protein phosphatase non-receptor type 6** | **Ptpn6** |  | **0.17** |
|  | **P29621** | **Serine protease inhibitor A3C** | **Serpina3c** |  | **4.79** |
|  | **P29788** | **Vitronectin** | **Vtn** |  | **2.76** |
|  | **P30275** | **Creatine kinase U-type, mitochondrial** | **Ckmt1** |  | **3.99** |
|  | **P35285** | **Ras-related protein Rab-22A** | **Rab22a** |  | **1.10** |
|  | **P37889** | **Fibulin-2** | **Fbln2** |  | **2.69** |
|  | **P39447** | **Tight junction protein ZO-1** | **Tjp1** |  | **0.44** |
|  | **P41317** | **Mannose-binding protein C** | **Mbl2** |  | **0.58** |
|  | **P43406** | **Integrin alpha-V** | **Itgav** |  | **1.67** |
|  | **P48758** | **Carbonyl reductase [NADPH] 1** | **Cbr1** |  | **3.98** |
|  | **P48999** | **Arachidonate 5-lipoxygenase** | **Alox5** |  | **0.17** |
|  | **P49935** | **Pro-cathepsin H** | **Ctsh** |  | **2.90** |
|  | **P50172** | **Corticosteroid 11-beta-dehydrogenase isozyme 1** | **Hsd11b1** |  | **1.17** |
|  | **P52196** | **Thiosulfate sulfurtransferase** | **Tst** |  | **2.02** |
|  | **P52825** | **Carnitine O-palmitoyltransferase 2, mitochondrial** | **Cpt2** |  | **2.10** |
|  | **P56135** | **ATP synthase subunit f, mitochondrial** | **Atp5j2** |  | **2.97** |
|  | **P56391** | **Cytochrome c oxidase subunit 6B1** | **Cox6b1** |  | **3.75** |
|  | **P61290** | **Proteasome activator complex subunit 3** | **Psme3** |  | **1.38** |
|  | **P62305** | **Small nuclear ribonucleoprotein E** | **Snrpe** |  | **1.83** |
|  | **P62320** | **Small nuclear ribonucleoprotein Sm D3** | **Snrpd3** |  | **2.04** |
|  | **P62331** | **ADP-ribosylation factor 6** | **Arf6** |  | **3.12** |
|  | **P62843** | **40S ribosomal protein S15** | **Rps15** |  | **2.90** |
|  | **P62858** | **40S ribosomal protein S28** | **Rps28** |  | **2.51** |
|  | **P62889** | **60S ribosomal protein L30** | **Rpl30** |  | **3.95** |
|  | **P63037** | **DnaJ homolog subfamily A member 1** | **Dnaja1** |  | **0.86** |
|  | **P70303** | **CTP synthase 2** | **Ctps2** |  | **0.77** |
|  | **P70404** | **Isocitrate dehydrogenase [NAD] subunit gamma 1, mitochondrial** | **Idh3g** |  | **2.86** |
|  | **P70424** | **Receptor tyrosine-protein kinase erbB-2** | **Erbb2** |  | **0.36** |
|  | **P84096** | **Rho-related GTP-binding protein RhoG** | **Rhog** |  | **1.88** |
|  | **P98078** | **Disabled homolog 2** | **Dab2** |  | **2.88** |
|  | **Q00896** | **Alpha-1-antitrypsin 1-3** | **Serpina1c** |  | **7.25** |
|  | **Q00897** | **Alpha-1-antitrypsin 1-4** | **Serpina1d** |  | **6.51** |
|  | **Q00898** | **Alpha-1-antitrypsin 1-5** | **Serpina1e** |  | **5.65** |
|  | **Q01279** | **Epidermal growth factor receptor** | **Egfr** |  | **3.10** |
|  | **Q03173** | **Protein enabled homolog** | **Enah** |  | **2.44** |
|  | **Q03734** | **Serine protease inhibitor A3M** | **Serpina3m** |  | **6.16** |
|  | **Q06185** | **ATP synthase subunit e, mitochondrial** | **Atp5i** |  | **3.36** |
|  | **Q06770** | **Corticosteroid-binding globulin** | **Serpina6** |  | **1.30** |
|  | **Q07563** | **Collagen alpha-1(XVII) chain** | **Col17a1** |  | **0.17** |
|  | **Q08857** | **Platelet glycoprotein 4** | **Cd36** |  | **3.85** |
|  | **Q3V3R1** | **Monofunctional C1-tetrahydrofolate synthase, mitochondrial** | **Mthfd1l** |  | **0.58** |
|  | **Q5I2A0** | **Serine protease inhibitor A3G** | **Serpina3g** |  | **4.89** |
|  | **Q5SV42** | **Leukocyte elastase inhibitor C** | **Serpinb1c** |  | **0.57** |
|  | **Q60604** | **Adseverin** | **Scin** |  | **4.23** |
|  | **Q60631** | **Growth factor receptor-bound protein 2** | **Grb2** |  | **3.45** |
|  | **Q60715** | **Prolyl 4-hydroxylase subunit alpha-1** | **P4ha1** |  | **2.30** |
|  | **Q60737** | **Casein kinase II subunit alpha** | **Csnk2a1** |  | **1.36** |
|  | **Q61176** | **Arginase-1** | **Arg1** |  | **1.38** |
|  | **Q61206** | **Platelet-activating factor acetylhydrolase IB subunit beta** | **Pafah1b2** |  | **0.38** |
|  | **Q61247** | **Alpha-2-antiplasmin** | **Serpinf2** |  | **2.17** |
|  | **Q62425** | **Cytochrome c oxidase subunit NDUFA4** | **Ndufa4** |  | **2.97** |
|  | **Q62426** | **Cystatin-B** | **Cstb** |  | **1.10** |
|  | **Q68FL6** | **Methionine--tRNA ligase, cytoplasmic** | **Mars** |  | **2.33** |
|  | **Q7TT37** | **Elongator complex protein 1** | **Ikbkap** |  | **0.97** |
|  | **Q80X76** | **Serine protease inhibitor A3F** | **Serpina3f** |  | **4.80** |
|  | **Q80YX1** | **Tenascin** | **Tnc** |  | **2.07** |
|  | **Q812C9** | **Retina-specific copper amine oxidase** | **Aoc2** |  | **1.44** |
|  | **Q8BH35** | **Complement component C8 beta chain** | **C8b** |  | **0.56** |
|  | **Q8BL66** | **Early endosome antigen 1** | **Eea1** |  | **2.57** |
|  | **Q8BTZ7** | **Mannose-1-phosphate guanyltransferase beta** | **Gmppb** |  | **1.86** |
|  | **Q8BU30** | **Isoleucine--tRNA ligase, cytoplasmic** | **Iars** |  | **1.88** |
|  | **Q8BUR4** | **Dedicator of cytokinesis protein 1** | **Dock1** |  | **0.17** |
|  | **Q8BYY9** | **Serine protease inhibitor A3B** | **Serpina3b** |  | **1.07** |
|  | **Q8C0C7** | **Phenylalanine--tRNA ligase alpha subunit** | **Farsa** |  | **0.86** |
|  | **Q8CGF7** | **Transcription elongation regulator 1** | **Tcerg1** |  | **2.36** |
|  | **Q8CGP7** | **Histone H2A type 1-K** | **Hist1h2ak** |  | **6.26** |
|  | **Q8CIE0** | **Serpin A11** | **Serpina11** |  | **0.77** |
|  | **Q8CIH5** | **1-phosphatidylinositol 4,5-bisphosphate phosphodiesterase gamma-2** | **Plcg2** |  | **0.17** |
|  | **Q8CIN4** | **Serine/threonine-protein kinase PAK 2** | **Pak2** |  | **1.10** |
|  | **Q8K183** | **Pyridoxal kinase** | **Pdxk** |  | **1.44** |
|  | **Q8K354** | **Carbonyl reductase [NADPH] 3** | **Cbr3** |  | **4.15** |
|  | **Q8K3J1** | **NADH dehydrogenase [ubiquinone] iron-sulfur protein 8, mitochondrial** | **Ndufs8** |  | **2.76** |
|  | **Q8K4Q8** | **Collectin-12** | **Colec12** |  | **0.58** |
|  | **Q8QZS1** | **3-hydroxyisobutyryl-CoA hydrolase, mitochondrial** | **Hibch** |  | **2.89** |
|  | **Q8R121** | **Protein Z-dependent protease inhibitor** | **Serpina10** |  | **1.17** |
|  | **Q8R180** | **ERO1-like protein alpha** | **Ero1l** |  | **0.17** |
|  | **Q8R1I1** | **Cytochrome b-c1 complex subunit 9** | **Uqcr10** |  | **1.49** |
|  | **Q8R1M2** | **Histone H2A.J** | **H2afj** |  | **6.60** |
|  | **Q8VCG4** | **Complement component C8 gamma chain** | **C8g** |  | **0.58** |
|  | **Q8VDL4** | **ADP-dependent glucokinase** | **Adpgk** |  | **0.17** |
|  | **Q91WG5** | **5~-AMP-activated protein kinase subunit gamma-2** | **Prkag2** |  | **0.44** |
|  | **Q91X52** | **L-xylulose reductase** | **Dcxr** |  | **1.07** |
|  | **Q91YN5** | **UDP-N-acetylhexosamine pyrophosphorylase** | **Uap1** |  | **0.56** |
|  | **Q91YP3** | **Putative deoxyribose-phosphate aldolase** | **Dera** |  | **0.57** |
|  | **Q91YW3** | **DnaJ homolog subfamily C member 3** | **Dnajc3** |  | **0.56** |
|  | **Q91Z53** | **Glyoxylate reductase/hydroxypyruvate reductase** | **Grhpr** |  | **1.63** |
|  | **Q920E5** | **Farnesyl pyrophosphate synthase** | **Fdps** |  | **1.66** |
|  | **Q924X2** | **Carnitine O-palmitoyltransferase 1, muscle isoform** | **Cpt1b** |  | **3.20** |
|  | **Q99KV1** | **DnaJ homolog subfamily B member 11** | **Dnajb11** |  | **2.14** |
|  | **Q99MR8** | **Methylcrotonoyl-CoA carboxylase subunit alpha, mitochondrial** | **Mccc1** |  | **3.53** |
|  | **Q9CPQ1** | **Cytochrome c oxidase subunit 6C** | **Cox6c** |  | **2.33** |
|  | **Q9CPQ8** | **ATP synthase subunit g, mitochondrial** | **Atp5l** |  | **2.16** |
|  | **Q9CQS8** | **Protein transport protein Sec61 subunit beta** | **Sec61b** |  | **2.41** |
|  | **Q9CR16** | **Peptidyl-prolyl cis-trans isomerase D** | **Ppid** |  | **2.74** |
|  | **Q9CRB3** | **5-hydroxyisourate hydrolase** | **Urah** |  | **1.96** |
|  | **Q9CWF2** | **Tubulin beta-2B chain** | **Tubb2b** |  | **4.89** |
|  | **Q9CX56** | **26S proteasome non-ATPase regulatory subunit 8** | **Psmd8** |  | **2.44** |
|  | **Q9D0F3** | **Protein ERGIC-53** | **Lman1** |  | **1.30** |
|  | **Q9D593** | **V-type proton ATPase subunit E 2** | **Atp6v1e2** |  | **0.17** |
|  | **Q9D8B3** | **Charged multivesicular body protein 4b** | **Chmp4b** |  | **1.17** |
|  | **Q9DBE0** | **Cysteine sulfinic acid decarboxylase** | **Csad** |  | **1.36** |
|  | **Q9DBM2** | **Peroxisomal bifunctional enzyme** | **Ehhadh** |  | **0.36** |
|  | **Q9DC69** | **NADH dehydrogenase [ubiquinone] 1 alpha subcomplex subunit 9, mitochondrial** | **Ndufa9** |  | **3.73** |
|  | **Q9DCS9** | **NADH dehydrogenase [ubiquinone] 1 beta subcomplex subunit 10** | **Ndufb10** |  | **3.53** |
|  | **Q9DCX2** | **ATP synthase subunit d, mitochondrial** | **Atp5h** |  | **3.82** |
|  | **Q9ER72** | **Cysteine--tRNA ligase, cytoplasmic** | **Cars** |  | **0.36** |
|  | **Q9ERS2** | **NADH dehydrogenase [ubiquinone] 1 alpha subcomplex subunit 13** | **Ndufa13** |  | **2.83** |
|  | **Q9JHH6** | **Carboxypeptidase B2** | **Cpb2** |  | **1.17** |
|  | **Q9QXY6** | **EH domain-containing protein 3** | **Ehd3** |  | **2.32** |
|  | **Q9WUR2** | **Enoyl-CoA delta isomerase 2, mitochondrial** | **Eci2** |  | **1.66** |
|  | **Q9WV54** | **Acid ceramidase** | **Asah1** |  | **1.97** |
|  | **Q9Z110** | **Delta-1-pyrroline-5-carboxylate synthase** | **Aldh18a1** |  | **2.49** |
|  | **Q9Z1G3** | **V-type proton ATPase subunit C 1** | **Atp6v1c1** |  | **1.36** |
|  | **Q9Z2M7** | **Phosphomannomutase 2** | **Pmm2** |  | **0.38** |

Normalized protein quantitative values were calculated as log2(Protein area/ Total protein area) X 10^6^

**Table S3.** The proteins involved in various biological pathways in *Acomys* and *Mus*

|  |  |  | ***Acomys*** | ***Mus*** |
| --- | --- | --- | --- | --- |
| **Functions** | **Pathway** | **Proteins** | **p-value** | **p-value** |
| **Inflammation** | **Complement and coagulation cascades** | ***Fga ,C3 ,C4b ,F2 ,Plg ,Serpinc1 ,Serpind1 ,Serping1 ,Cfi ,A2mp ,Fgb ,Fgg* , F13a1 ,F10 ,C5 ,C1qb ,C2 ,Serpinf2 ,C8b ,Cpb2 ,Kng1 ,Cfd ,C9 ,Serpina1a , C4bpa ,Klkb1 ,Mbl2 ,Serpina1c ,F12 ,C1sb ,C1sa ,C8a ,C8g** | **8E-08** | **5.2E-33** |
|  | **Regulation of actin cytoskeleton** | ***Pdgfrb ,Gsn ,F2 ,Gna13 ,Rac1 ,Rock2 ,Mylpf ,Rac2 ,Wasf2 ,Arpc2 ,Arpc1b* , Actn3 ,Itgb1 ,Cfl1 ,Fgfr2 ,Ezr ,Msn ,Gna12 ,Cfl2 ,Arpc4 ,Actb ,Rac3 ,Cdc42 , Pfn1 ,Myl12b ,Iqgap2 ,Myh9 ,Arpc5 ,Myl9 ,Arpc3 ,Rhoa ,Itgb5 ,Itga5 ,Itgb2 , Itgb7 ,Myl2 ,Rock1 ,Egfr ,Cyfip2 ,Cyfip1 ,Mylk2 ,Arpc1a ,Fgfr1,Map2k2, Fgfr4,Scin ,Arhgef1 ,Dock1 ,Vav1 ,Pak2** | **2E-16** | **6.6E-06** |
|  | **Leukocyte transendothelial migration** | ***Ctnna1 ,Rac1 ,Rock2 ,Vasp ,Mylpf ,Ctnnb1 ,Rac2 ,Cybb ,Ctnna2* ,Actb , Cdc42 ,Rap1a ,Myl12b ,Rap1b ,Myl9 ,Gnai3 ,Rhoa ,Mapk14 ,Myl2 ,Rock1 , Mapk13 ,Itgb2 ,Gnai1 ,Actn3 ,Thy1 ,**Gnai2 ,Itgb1 ,Ezr ,Msn **,Ncf2,Icam1 , Vav1 ,Mmp9 ,Ncf4 ,Pecam1 ,Plcg2** | **7E-12** | **5E-07** |
| **Cellular Junction** | **Focal adhesion** | ***Pdgfrb ,Col3a1 ,Col1a1 ,Tln1 ,Fyn ,Rac1 ,Rock2 ,Vasp ,Mylpf ,Col1a2 ,Ctnnb1 , Col6a1 ,Rac2 ,Thbs3 ,Thbs4* ,Itgb1 ,Actb ,Rac3 ,Cdc42 ,Rap1a ,Myl12b ,Zyx , Flna ,Flnc ,Rap1b ,Myl9 ,Rhoa ,Actn3 ,Capn2 ,Itgb5 ,Itga5 ,Itgb7 ,Vtn ,Myl2 , Rock1 ,Egfr ,Col6a2 ,Thbs2 ,Grb2 ,Lama5 ,Tln2 ,Tnc ,Mylk2 ,Chad ,Map2k1 , Dock1 ,Pak2 ,Vav1** | **5E-11** | **7.6E-10** |
|  | **Tight junction** | **Myh7b ,Gnai1 ,Actn3 ,Gnai2 ,Myh3 ,Myh8 ,Ctnna1 ,Actb ,Cdc42 ,Ppp2cb , Ppp2ca ,Mylpf ,Ctnnb1 ,Myh6 ,Yes1 ,Myl12b ,Myh4 ,Myh1 ,Ctnna2 ,Ppp2r1b , Myh9 ,Myh7 ,Myl9 ,Rab3b ,Gnai3 ,Rhoa ,Cdk4** | **4E-13** | **NA** |
|  | **Adherens junction** | ***Ctnna1 ,Fyn ,Rac1 ,Ctnnb1 ,Yes1 ,Rac2 ,Ctnna2* ,Actn3 ,Actb ,Rac3 ,Cdc42 , Wasf2 ,Rhoa ,Ptpn6 ,Egfr ,Fgfr1** | **9E-07** | **NA** |
| **Endocytosis** | **Phagosome** | ***Coro1a ,C3 ,Mpo ,Atp6v0d1 ,Sec61a1 ,Atp6v1b2 ,Rac1 ,Thbs3 ,Cd36 ,Mrc2 , Atp6v1h ,Dync1h1 ,Thbs4* ,Tubb1 ,Sec22b ,Dync1i2 ,Tuba1b ,Itgb1 ,Calr ,Rab5c , Canx ,Atp6v1e2,Rab7a ,Atp6v1d ,Actb ,Rab5b ,Tuba4a ,Tubb5 ,Mrc1 ,Tubb2a , Tubb6 ,Rab5a ,Tubb4a ,Tubb3 ,Tuba8 ,Itgb5 ,Itga5 ,Itgb2 ,Eea1 ,Stx12 ,Thbs2 , Ncf2,H2-Q10 ,M6pr ,Mbl2 ,Ncf4 ,Cybb ,Tfrc ,Colec12 ,Tubb2b** | **6E-19** | **1E-10** |
|  | **Endocytosis** | **Kit ,Ap2a1 ,Ap2a2 ,Hspa1b ,Fgfr2 ,Rab5c ,Dnm2 ,Vps4b ,Rab11b ,Nedd4 , Rab7a ,Cdc42 ,Rab5b ,Ap2m1 ,Hspa1a ,Cltc ,Ehd2 ,Rab5a ,Chmp2a ,Ap2b1 , Ehd4 ,Sh3glb1 ,Rhoa ,Pdcd6ip ,Ehd1 ,Fgfr4 ,Rab11a ,Sh3gl1 ,Vps4a** | **2E-07** | **NA** |
|  | **Fc gamma R-mediated phagocytosis** | ***Hck ,Gsn ,Lyn ,Rac1 ,Vasp ,Rac2 ,Arpc2 ,Arpc1b* ,Marcks ,Dnm2 ,Cfl2 ,Arpc4 , Cdc42 ,Wasf2 ,Arpc5 ,Arpc3 ,Cfl1 ,Scin ,Map2k2,Vav1 ,Prkcd ,Dock2 ,Plcg2** | **1E-08** | **1.8E-06** |
| **Protein Synthetic/ Catabolic Process** | **Ribosome** | **Rpl7a, Rpl27a, Rps16 Rpsa, Rpl13a, Rpl3, Rpl12, Rpl18, Rpl28, Rps20, Rpl26, Rpl27, Rps7, Rps8, Rps15a, Rps14, Rps23, Rps18, Rps11, Rps13, Rpl23, Rps26, Rpl31, Rps3,  Rpl8, Rps27a, Uba52, Rps17, Rps10, Rpl22, Rps5, Rplp2, Rpl35, Rpl14, Rpl11, Rps19, Rpl4** | **2E-27** | **NA** |
|  | **Spliceosome** | ***Hspa1b ,Hnrnpa1 ,Hnrnpa3 ,Sf3a1 ,Snrpb2* ,Eftud2 ,Dhx15 ,Snrpb ,Snrpa1 ,Pcbp1 , Hnrnpk ,Snrpd1 ,Snrpd2 ,Srsf3 ,Hspa1a ,Srsf2 ,Snrpa ,Srsf1 ,Srsf7 ,Sf3b3 ,Prpf19 , Hnrnpm ,Ddx39b ,Tra2b ,Ncbp1 ,Hnrnpu ,Eif4a3 ,Hnrnpc ,U2af1 ,Rbmx ,U2af2 , Cdc5l ,Snrnp200** | **7E-09** | **1.9E-05** |
|  | **Proteasome** | **Psmb1, Psmd4, Psmd14, Psmb5, Psma3, Psmc3, Psmb9, Psmc4, Psmc5, Psmb7, Psme2,  Psmb4, Psmd1, Psmb6, Psmd6, Psma6, Psma4, Psmb3, Psmd13, Psma5** | **4E-14** | **NA** |
|  | **Protein processing in endoplasmic reticulum** | ***Hspa1b ,Sec61a1 ,Sec23a ,Txndc5 ,Rrbp1 ,Hyou1* ,Prkcsh ,Ddost ,Pdia4 ,P4hb ,Calr , Hspa5 ,Pdia3 ,Canx ,Stt3a ,Hspa4l ,Erp29 ,Sec31a ,Hspa1a ,Hsph1 ,Ssr4 ,Uggt1 ,Eif2s1 , Ganab ,Ckap4 ,Pdia6 ,Sar1b ,Ssr1 ,Sec23b ,Lman2 ,Ssr3 ,Cul1 ,Rpn1 ,Cryab ,Dnaja1 ,Edem3 , Sec31b ,Mogs ,Sec13 ,Plaa ,Dad1 ,Ufd1l ,Ero1l ,Dnajc3 ,Lman1** | **3E-14** | **1.8E-08** |
| **Neural development/ degeneration** | **Axon guidance** | **Gnai1, Gnai2, Itgb, Cfl1, Fyn, Cfl2, Rac3, Cdc4, Rac1, Rock2, Rac2, Gnai3, Rhoa** | **1E-05** | **NA** |
| **Metabolic process** | **Metabolic pathways** | ***Acacb ,Mgll ,Hsd17b12 ,Gnpda1 ,Pfkl ,Bpgm ,Akr1b7 ,Tsta3 ,Lta4h ,Aco1 ,Tkt , Acsl1 ,Akr1b8 ,Acadm ,Gfpt1 ,Pfkm ,Shmt1 ,Acadvl ,Atp6v0d1 ,Atp6v1b2 , G6pd2 ,G6pdx ,Hk3 ,Aldh3b1 ,Echs1 ,Hadha ,Atp6v1h ,Polr2b ,Eprs ,Sdha , Ces1d ,Impa2 ,Ndufa10 ,Sdhb ,Pgm1 ,Idh3a ,Pgd*  ,Ddost ,Hprt2,Got3,Ckm , Alad ,Nme2,Ldhb ,Cox4i2,Pnp ,Adh6,Adssl2,Oat ,Fah ,Stt3a ,Aldh3,Atp6v1e2, Idh3,Atp6v1d ,Pafah1b2,Ugt1a3,Xdh ,Nme3,Ogdh ,Phgdh ,Ugt1a9 ,Ugt1a1 , Ces1e ,Gart ,Ugt1a7c ,Ganab ,Pgp ,Rpn2,Ndufv2,Mthfd2,Taldo2,Hadhb ,Aco3, Gnpda3,Atic ,Cs ,Pdhb ,Uqcrc3,Pgam2,Cmpk2,Aldh1a4,Aldh9a2,Acox2,Cad , Pgam2 ,Aoc3 ,Mtco2 ,Amy1 ,Gpi ,Me1 ,Glul ,Fasn ,Man2a1 ,Hal ,Aldh3a2 , Ahcy ,Hsd17b4 ,Pon1 ,Ctps1 ,Anpep ,Ckb ,Pfas ,Acaca ,Sord ,Mogs ,Dlat , Acaa2 ,Acsl5 ,Acaa1b ,Ndufs1 ,Atp5c1 ,Gldc ,Uap1 ,Ugp2 ,Mat2b ,Lap3 , Atp5f1 ,Cyc1 ,Tktl2 ,Ehhadh ,Atp5h ,Cyp4f14 ,Aldh6a1 ,Gda ,Pfkp ,Pmm2 , Ndufs ,Dbt ,Fbp2,Aox2,Qdpr ,Synj2 ,Ada ,Cbr2 ,Gusb ,Umps ,Ass1 ,Ckmt1 , Cbr1 ,Alox5 ,Inpp1 ,Hsd11b1 ,Cox6b1 ,Dad1 ,Ctps2 ,Atp5i ,Uap1l1 ,Mthfd1l , Arg1 ,Pafah1b2 ,Plcg2 ,Cbr3 ,Adpgk ,Fdps ,Mccc1 ,Ndufa9 ,Ndufb10 ,Aldh18a1** | **6E-07** | **1.8E-13** |
|  | **Glutathione metabolism** | ***Gpx1 ,Gpx3 ,Gstm5 ,G6pd2 ,G6pdx ,Gstm7 ,Pgd ,Gsr* ,Gstp2 ,Idh2 ,Gstm1 , Gstm2 ,Anpep ,Lap3 ,Ggct ,Gsto1** | **9E-05** | **1.7E-08** |
|  | **Oxidative phosphorylation** | ***Atp6v0d1 ,Atp6v1h ,Ndufa10 ,Sdhb* ,Atp6v1e1 ,Atp6v1d ,Uqcrc2 ,Mtco2 ,Ndufs1 , Atp5c1 ,Atp5f1 ,Ndufs3 ,Cox6b1 ,Atp6v1b2 ,Atp5i ,Sdha ,Ndufa9 ,Ndufb1** | **NA** | **0.0006** |
| **Carbohydtrate metabolic  process** | **Glycolysis /Gluconeogenesis** | ***Pfkl ,Bpgm ,Pfkm ,Hk3 ,Aldh3b1 ,Pgm1* ,Ldhb ,Aldh2 ,Pgam1 ,Aldh1a3 ,Pgam2 , Gpi ,Aldh3a2 ,Dlat ,Pfkp , Adpgk** | **0.0007** | **0.0001** |
|  | **Amino sugar and nucleotide sugar metabolism** | ***Tsta3 ,Gfpt1 ,Hk3 ,Pgm1 ,Cyb5r3* ,Gnpda1 ,Gpi ,Chi3l1 ,Uap1 ,Ugp2 ,Pmm2 , Uap1l1 ,Chit1** | **3E-07** | **0.0033** |
| **Fatty acid metabolism** | **Fatty acid metabolism** | ***Acsl1 ,Acadm ,Echs1 ,*Hadhb ,Acox1,Aldh2*,*Aldh3a2 ,Acadvl ,Hadha , Acaa2 ,Acsl5 ,Ehhadh *,*Cpt1b** | **NA** | **5.6E-05** |
| **signaling pathway** | **Wnt signaling pathway** | **Ruvbl1, Rac3 ,Ppp2cb ,Rac1 ,Ppp2ca ,Rock2 ,Ctnnb1 ,Rac2 ,Ppp2r1b ,Cacybp ,Rhoa** | **0.0024** | **NA** |
|  | **PPAR signaling pathway** | **Fabp4 ,Me1 ,Acsl1 ,Adipoq ,Acsl5 ,Ehhadh ,Apoa2 ,Cd36 ,Cpt1b** | **NA** | **4E-06** |
|  | **MAPK Signalling** | **Pdgfrb ,Fgfr2 ,Gna12 ,Rac3 ,Cdc42 ,Rap1a ,Rac1 ,Rac2 ,Flna ,Flnc ,Rap1b ,Fgfr1 ,Fgfr4 , Rps6ka3 ,Map2k1 ,Ppp5c** | **0.0054** | **NA** |
| **Others** | **ECM-receptor interaction** | ***Col3a1 ,Col1a1 ,Col1a2 ,Col6a1 ,Hspg2 ,Thbs3 ,Thbs4* ,Itgb2,Itgb5 ,Itga5 ,Itgb7 , Vtn ,Col6a2 ,Thbs2 ,Lama5 ,Tnc ,Cd44 ,Cd36 ,Chad** | **0.0004** | **4.6E-08** |
|  | **Vasopressin-regulated water reabsorption** | **Dctn1 ,Dync1i2 ,Rab5c ,Nsf ,Rab11b ,Rab5b ,Gnas ,Gnas ,Arhgdia ,Rab5a ,Dync1h1 ,Rab11a** | **2E-08** | **NA** |
|  |  | **Legend : *Black Bold Italic* *: High FC in Acomys, High FC in Mus* Black Bold : High FC in *Acomys*, Low FC in *Mus*  Red Bold : Low FC in *Acomys*, High FC in *Mus* Blue Bold : Only in *Acomys* and High FC  Violet Bold : Only in *Mus* and High FC** |  |  |

**Table S4**. Identified cytoskeletal and cuticular keratins from *Acomys* and *Mus* associated with

wound healing over 14 days*.*

|  |  |  | ***Acomys*** | | | | | ***Mus*** | | | | |
| --- | --- | --- | --- | --- | --- | --- | --- | --- | --- | --- | --- | --- |
| **Accession** | **Protein Description** | **Gene** | **0d** | **3d** | **5d** | **7d** | **14d** | **0d** | **3d** | **5d** | **7d** | **14d** |
| **Q8CCX5** | **Keratin-like protein KRT222** | **Krt222** | **5.02** | **4.89** | **5.20** | **5.19** | **4.99** | **4.67** | **4.07** | **4.08** | **4.33** | **4.71** |
| **Q8VCW2** | **Keratin, type I cytoskeletal 25** | **Krt25** | **8.62** | **9.29** | **8.66** | **9.02** | **8.58** | **8.25** | **7.27** | **8.55** | **8.72** | **8.14** |
| **Q9Z320** | **Keratin, type I cytoskeletal 27** | **Krt27** | **8.54** | **9.29** | **8.62** | **9.02** | **8.55** | **8.27** | **7.27** | **8.55** | **8.71** | **8.20** |
| **A6BLY7** | **Keratin, type I cytoskeletal 28** | **Krt28** | **7.89** | **6.99** | **5.60** | **7.01** | **7.70** | **6.21** | **5.89** | **6.08** | **5.96** | **6.60** |
| **Q8K0Y2** | **Keratin, type I cuticular Ha3-I** | **Krt33a** | **4.87** | **2.93** | **6.03** | **1.44** | **3.41** | **3.87** | **6.73** | **4.71** | **3.24** | **6.89** |
| **Q9D646** | **Keratin, type I cuticular Ha4** | **Krt34** | **5.09** | **4.18** | **6.19** | **2.59** | **4.16** | **4.47** | **1.84** | **4.82** | **4.35** | **7.01** |
| **Q6IFX3** | **Keratin, type I cytoskeletal 40** | **Krt40** | **7.79** | **7.03** | **6.06** | **7.14** | **7.70** | **5.84** | **6.15** | **6.08** | **6.14** | **6.90** |
| **Q6IFX2** | **Keratin, type I cytoskeletal 42** | **Krt42** | **8.95** | **8.55** | **8.36** | **8.93** | **8.94** | **8.16** | **7.65** | **7.91** | **7.92** | **8.61** |
| **Q9Z331** | **Keratin, type II cytoskeletal 6B** | **Krt6b** | **8.58** | **9.32** | **8.99** | **9.52** | **9.24** | **9.33** | **8.05** | **8.78** | **8.23** | **9.25** |
| **Q9R0H5** | **Keratin, type II cytoskeletal 71** | **Krt71** | **8.52** | **8.53** | **7.83** | **8.16** | **8.16** | **7.99** | **6.86** | **7.37** | **7.32** | **8.23** |
| **Q6IME9** | **Keratin, type II cytoskeletal 72** | **Krt72** | **8.15** | **8.49** | **7.89** | **8.32** | **8.02** | **7.81** | **7.04** | **7.62** | **7.45** | **8.30** |
| **Q6NXH9** | **Keratin, type II cytoskeletal 73** | **Krt73** | **8.49** | **9.08** | **8.39** | **8.93** | **8.48** | **8.14** | **7.70** | **8.14** | **8.03** | **8.64** |
| **Q6IFZ9** | **Keratin, type II cytoskeletal 74** | **Krt74** | **8.19** | **8.52** | **7.82** | **8.07** | **8.06** | **7.51** | **6.80** | **7.33** | **7.28** | **8.01** |
| **Q8BGZ7** | **Keratin, type II cytoskeletal 75** | **Krt75** | **9.30** | **9.72** | **9.33** | **9.82** | **9.63** | **9.45** | **8.38** | **9.03** | **8.66** | **9.47** |
| **Q3UV17** | **Keratin, type II cytoskeletal 2** | **Krt76** | **8.97** | **9.60** | **8.98** | **9.59** | **9.12** | **8.74** | **8.06** | **8.56** | **8.38** | **8.91** |
| **Q6IFZ6** | **Keratin, type II cytoskeletal 1b** | **Krt77** | **8.72** | **8.71** | **8.03** | **8.50** | **8.48** | **9.39** | **7.05** | **7.66** | **7.60** | **8.66** |
| **Q8VED5** | **Keratin, type II cytoskeletal 79** | **Krt79** | **8.58** | **9.00** | **8.57** | **9.07** | **8.77** | **8.78** | **7.71** | **8.49** | **8.04** | **9.05** |
| **Q9ERE2** | **Keratin, type II cuticular Hb1** | **Krt81** | **6.08** | **2.84** | **6.35** | **6.70** | **5.66** | **5.82** | **3.13** | **4.38** | **3.05** | **7.77** |
| **Q99M74** | **Keratin, type II cuticular Hb2** | **Krt82** | **5.53** | **4.59** | **5.32** | **5.49** | **4.92** | **4.27** | **2.84** | **2.77** | **3.67** | **5.10** |
| **Q6IMF0** | **Keratin, type II cuticular Hb3** | **Krt83** | **5.92** | **4.76** | **6.23** | **5.02** | **5.56** | **5.61** | **2.54** | **4.41** | **3.05** | **7.51** |
| **Q99M73** | **Keratin, type II cuticular Hb4** | **Krt84** | **7.38** | **7.07** | **6.77** | **7.11** | **6.90** | **6.66** | **5.51** | **6.04** | **5.94** | **6.68** |
| **Q9Z2T6** | **Keratin, type II cuticular Hb5** | **Krt85** | **5.85** | **5.16** | **6.56** | **6.94** | **5.23** | **6.12** | **6.02** | **3.77** | **4.36** | **7.85** |
| **P97861** | **Keratin, type II cuticular Hb6** | **Krt86** | **6.03** | **4.56** | **5.36** | **5.09** | **5.52** | **5.82** | **3.13** | **4.38** | **3.05** | **7.66** |

**Normalized protein quantitative values were calculated as log2(Protein area/ Total protein area) X 10^6^**

**Table S5.** Identified serine protease inhibitors (SERPINs) from *Acomys* and *Mus* associated with

wound healing over 14 days*.*

|  |  |  | ***Acomys*** | | | | | ***Mus*** | | | | |
| --- | --- | --- | --- | --- | --- | --- | --- | --- | --- | --- | --- | --- |
|  |  |  | **0 day** | **3 days** | **5 days** | **7 days** | **14days** | **0 day** | **3 days** | **5 days** | **7 days** | **14days** |
| **Accession** | **Protein Description** | **Gene** | **Quantitative Value** | | | | | | | | | |
| **Q6P4P1** | **Serine protease inhibitor A3A** | **Serpina3a** | **1.04** | **2.50** | **2.04** | **2.79** | **2.62** | **0.36** | **4.22** | **2.59** | **3.73** | **1.17** |
| **Q80X76** | **Serine protease inhibitor A3F** | **Serpina3f** |  |  |  |  |  | **4.80** | **7.66** | **6.68** | **6.99** | **5.87** |
| **Q8BYY9** | **Serine protease inhibitor A3B** | **Serpina3b** |  |  |  |  |  | **1.07** | **1.17** |  | **1.58** | **1.39** |
| **Q8VHP7** | **Leukocyte elastase inhibitor B** | **Serpinb1b** | **2.66** | **5.07** | **5.25** | **4.55** | **3.33** | **1.49** | **4.68** | **4.31** | **4.51** | **3.17** |
| **P12388** | **Plasminogen activator inhibitor 2, macrophage** | **Serpinb2** |  |  |  |  |  | **2.98** | **2.04** | **3.21** | **3.65** | **2.29** |
| **P22599** | **Alpha-1-antitrypsin 1-2** | **Serpina1b** |  |  |  |  |  | **7.10** | **8.39** | **8.20** | **8.42** | **6.69** |
| **P29621** | **Serine protease inhibitor A3C** | **Serpina3c** |  |  |  |  |  | **4.79** | **7.50** | **6.68** | **7.00** | **5.85** |
| **Q00896** | **Alpha-1-antitrypsin 1-3** | **Serpina1c** |  |  |  |  |  | **7.25** | **8.92** | **8.22** | **8.69** | **6.79** |
| **Q00897** | **Alpha-1-antitrypsin 1-4** | **Serpina1d** |  |  |  |  |  | **6.51** | **8.00** | **7.65** | **7.92** | **6.11** |
| **Q00898** | **Alpha-1-antitrypsin 1-5** | **Serpina1e** |  |  |  |  |  | **5.65** | **6.93** | **6.88** | **6.37** | **4.81** |
| **Q03734** | **Serine protease inhibitor A3M** | **Serpina3m** |  |  |  |  |  | **6.16** | **8.59** | **7.98** | **8.28** | **6.97** |
| **Q06770** | **Corticosteroid-binding globulin** | **Serpina6** |  |  |  |  |  | **1.30** | **4.08** | **3.95** | **4.86** | **2.30** |
| **Q5I2A0** | **Serine protease inhibitor A3G** | **Serpina3g** |  |  |  |  |  | **4.89** | **7.43** | **6.67** | **6.81** | **5.78** |
